# Standardization and harmonization of distributed multi-center proteotype analysis supporting precision medicine studies

**DOI:** 10.1038/s41467-020-18904-9

**Published:** 2020-10-16

**Authors:** Yue Xuan, Nicholas W. Bateman, Sebastien Gallien, Sandra Goetze, Yue Zhou, Pedro Navarro, Mo Hu, Niyati Parikh, Brian L. Hood, Kelly A. Conrads, Christina Loosse, Reta Birhanu Kitata, Sander R. Piersma, Davide Chiasserini, Hongwen Zhu, Guixue Hou, Muhammad Tahir, Andrew Macklin, Amanda Khoo, Xiuxuan Sun, Ben Crossett, Albert Sickmann, Yu-Ju Chen, Connie R. Jimenez, Hu Zhou, Siqi Liu, Martin R. Larsen, Thomas Kislinger, Zhinan Chen, Benjamin L. Parker, Stuart J. Cordwell, Bernd Wollscheid, Thomas P. Conrads

**Affiliations:** 1 Thermo Fisher Scientific GmbH, Hanna-Kunath Str. 11, Bremen, 28199 Germany; 2 Gynecologic Cancer Center of Excellence, Henry M. Jackson Foundation for the Advancement of Military Medicine, Inc., Uniformed Services University and Walter Reed National Military Medical Center, 8901 Wisconsin Avenue, Bethesda, 20889 MD USA; 3 Thermo Fisher Scientific, Paris, France; 4 Thermo Fisher Scientific, Precision Medicine Science Center, Cambridge, MA USA; 5 Institute of Translational Medicine, Department of Health Sciences and Technology, ETH Zurich, Switzerland; 6 Swiss Institute of Bioinformatics, Lausanne, Switzerland; 7 Thermo Fisher Scientific Co. Ltd, Shanghai, China; 8 Leibniz-Institut für Analytische Wissenschaften—ISAS—e.V., Bunsen-Kirchhoff-Straße 11, 44139 Dortmund, Germany; 9 Institute of Chemistry, Academia Sinica, 128 Academia Road, Section 2, Nankang Taipei, 11529 Taiwan; 10 Department of Medical Oncology, Cancer Center Amsterdam, Amsterdam UMC De Boelelaan 1117, 1081 HV Amsterdam, the Netherlands; 11 Stoller Biomarker Discovery Centre, Institute of Cancer Sciences, Faculty of Medical and Human Sciences, University of Manchester, Manchester, M13 9PL United Kingdom; 12 Shanghai Institute of Materia Medica, Chinese Academy of Sciences, 555 Zuchongzhi Road, Shanghai, 201203 China; 13 BGI-SHENZHEN, Beishan Road, Yantian District, Shenzhen, 518083 Guangdong China; 14 Department of Biochemistry and Molecular Biology, University of Southern Denmark, Campusvej 55, Odense M, DK-5230 Denmark; 15 Princess Margaret Cancer Centre, 101 College Street PMCRT 9-807, Toronto, ON M5G 1L7 Canada; 16 National Translational Science Center for Molecular Medicine, Xi’an, 710032 China; 17 Department of Cell Biology, School of Basic Medicine, Air Force Medical University, Xi’an, 710032 China; 18 Sydney Mass Spectrometry, The University of Sydney, NSW 2006 Sydney, Australia; 19 Medizinische Fakultät, Medizinisches Proteom-Center (MPC), Ruhr-Universität Bochum, 44801 Bochum, Germany; 20 Department of Chemistry, College of Physical Sciences, University of Aberdeen, Aberdeen AB243FX Scotland, UK; 21 School of Life and Environmental Science, The University of Sydney, NSW 2006 Sydney, Australia; 22 Women’s Health Integrated Research Center, Women’s Service Line, Inova Health System, 3289 Woodburn Bldg, Annandale, VA 22003 USA

**Keywords:** Proteomics, Cancer, Systems biology, Medical research

## Abstract

Cancer has no borders: Generation and analysis of molecular data across multiple centers worldwide is necessary to gain statistically significant clinical insights for the benefit of patients. Here we conceived and standardized a proteotype data generation and analysis workflow enabling distributed data generation and evaluated the quantitative data generated across laboratories of the international Cancer Moonshot consortium. Using harmonized mass spectrometry (MS) instrument platforms and standardized data acquisition procedures, we demonstrate robust, sensitive, and reproducible data generation across eleven international sites on seven consecutive days in a 24/7 operation mode. The data presented from the high-resolution MS1-based quantitative data-independent acquisition (HRMS1-DIA) workflow shows that coordinated proteotype data acquisition is feasible from clinical specimens using such standardized strategies. This work paves the way for the distributed multi-omic digitization of large clinical specimen cohorts across multiple sites as a prerequisite for turning molecular precision medicine into reality.

## Abstract (web summary)

Distributed multi-omic digitization of clinical specimen across multiple sites is a prerequisite for turning molecular precision medicine into reality. Here, the authors show that coordinated proteotype data acquisition is feasible using standardized MS data acquisition and analysis strategies.

## Introduction

Many precision medicine projects have emerged following the launch of the Cancer Moonshot initiative, which aims to accelerate cancer research by enhancing cancer patient management through improved early detection, patient stratification, and monitoring for therapeutic efficacy, outcome, and recurrence. Genomic applications have undoubtedly been the major driving force for current precision medicine approaches, especially in the field of oncology, as genomic studies have revealed important and targetable cancer driver genes and mutations. However, the genotype of a patient alone is often not sufficient to support clinical decision making^1–3^. Additional data types are necessary to bridge the gap in predicting (clinical) phenotype from genotype. Apart from clinical, lifestyle, and mobile health data, molecular data either alone or in combination with other available health data are anticipated to support improved clinical decisions for cancer patient management including those related to quality, safety, efficiency, and effectiveness of health care.

Information about the proteotype, defined as the actual state of the proteome, the identities of proteins/proteoforms, their quantities, organization in time and space, and relatedness to the genotype (so-called proteogenomics), represents a major scientific pursuit the results of which are anticipated to substantially add to our understanding of a broad range of human maladies, including cancer. Proteogenomics has emerged as a promising approach to advance basic, translational, and clinical research^4^. The promise of proteogenomics has prompted assembly of several networks and consortiums as the Applied Proteogenomics Organizational Learning and Outcomes network (APOLLO) and the International Cancer Proteogenome Consortium (ICPC), which aim to demonstrate the critical role of proteogenomics in precision medicine and, ultimately, to incorporate proteogenomic-derived insights into patient care^5–7^. These initiatives rely on collaboration between various centers, often in a highly distributed fashion, and have committed to open data sharing, which can uniquely provide information at an unprecedented population scale, representative of global patient diversity. Distributed data generation has been achieved in the field of genomics, but not yet in the field of proteogenomics. There is thus a strong need for analytical mass spectrometry (MS) strategies that support proteotype analysis to deliver reliable and reproducible quantitative data that can be assembled and evaluated in a consistent and harmonized fashion. Such a capability has been demonstrated for MS-based proteotyping applications using targeted data acquisition methods and internal standards^8^, typically in clinical and late-stage translational research. However, development and assessment of standardized proteotyping workflows with acceptable quantitative performance at early stages of translational research remain a largely unmet need.

The proteome is enormously complex; it has recently been estimated to include over six million proteoforms^9^. Discovery-driven clinical proteomic workflows have focused on improving coverage and reproducible quantitation of the proteome. Historic discovery-driven methods employing data-dependent acquisition (DDA) provide both peptide identification information and relative measures of peptide abundance^10–13^, however suffer from variable quantitative performance across samples^10,11^. These shortcomings have led to the development of methods focused on improved stability and quantitative reproducibility such as by data-independent acquisition (DIA) techniques^14–18^. DIA-based strategies enable unbiased measurement of peptide precursor ions (MS1 spectra) as well as peptide fragment ions (MS2 spectra) and leverage mass spectrometry technologies to generate accurate and reproducible peptide measurements towards maximizing proteome quantitation. DIA-based strategies focusing on quantitation of intact peptide ion abundances extracted from retention-time aligned MS1 spectra, including accurate mass and time tag (AMT), the hybrid data acquisition and processing strategy pSMART, and hyper-reaction monitoring techniques, have demonstrated excellent analytical reproducibility^14,19^ and quantitative accuracy^17^. As these techniques afford reliable peptide quantitation and increased proteome coverage compared to DDA techniques, they are well-positioned to support high-throughput clinical proteomic analyses for precision medicine applications/workflows that are often challenged by limited amounts of input material and large numbers of samples per study cohort.

To achieve reproducible and stable quantitative data sets and to facilitate harmonized implementation, standardization of DIA methods will be necessary. Toward this goal, a recent publication described the application of DIA methods to establish digital proteome maps of human tissue samples with the goal of creating prospective, digital proteome biobanks of clinical biospecimens supporting real-time and retrospective data analyses^20^. Moreover, optimized synthetic^21^ and internal^22^ peptide standards have been developed to facilitate peptide retention-time alignment procedures and support facile comparison of DIA datasets generated at different analytical sites. Further, efforts to benchmark software platforms^23^ and statistical methods^24^ for DIA data analysis have been described, as has the generation of comprehensive peptide spectral libraries^25^. Recently, performance benchmarks for DIA data acquisition, specifically the application of the so-called SWATH DIA-MS approach, were used in analyses of a complex cell line standard in 11 laboratories^26^. This study revealed consistent quantitation of more than 4000 proteins from HEK293 cells across all laboratories.

To continue to expand the implementation of DIA workflows and integrate them into routine clinical sample analyses, the incorporation of benchmarked standards and standardized QC routines are necessary to maximize the accessibility of downstream data and empower team-driven science initiatives. Here we report on the performance of a high-throughput (e.g. 100 proteins quantified/min of analysis time), streamlined, and QC-benchmarked HRMS1-DIA workflow implemented in a continuous operational mode for seven consecutive days in eleven internationally-distributed labs followed by centralized data processing. A quality control (QC) system was developed to monitor the entire workflow performance, promptly identify decrements in instrument performance, and guide to trigger troubleshooting when necessary, ensuring high levels of data quality are maintained to achieve the throughput necessary for large clinical cohort studies. Quantitative performance was evaluated with a well-established label-free quantitation sample set at each laboratory through the entire study, enabling distributed and longitudinal acquisition of data that can be compared and normalized during big data analysis. Controlled samples^23^ included *E. coli*, yeast, and human cell line peptide digests were combined at fixed ratios to mimic biological samples and provided proof-of-concept feasibility and performance of this streamlined workflow. Experiments were then extended to actual clinical tissue samples from well-defined ovarian cancer histotypes, namely high-grade serous and clear cell ovarian cancers, to further demonstrate the utility of the standardized HRMS1-DIA strategy for routine clinical proteotype analyses.

## Results

### Implementation of a QC-benchmarked, HRMS1-DIA workflow

This report details the analytical performance and reproducibility of a standardized and QC-benchmarked HRMS1-DIA workflow intended to achieve quantitative proteotype analysis studies of large cohorts across several centers and in turn to support precision medicine projects. To address the needs of a robust and high-throughput workflow compatible with large-cohort studies, a 60-minute capillary flow LC gradient using 1.2 µL min^−1^ analytical flow rate was applied in all the analyses. Primary DIA data acquisition was performed on either the Easy-nLC 1200 or the Ultimate 3000 RSLC liquid chromatography systems coupled online with Q Exactive HF mass spectrometers (Thermo Fisher).

**Fig. 1:**
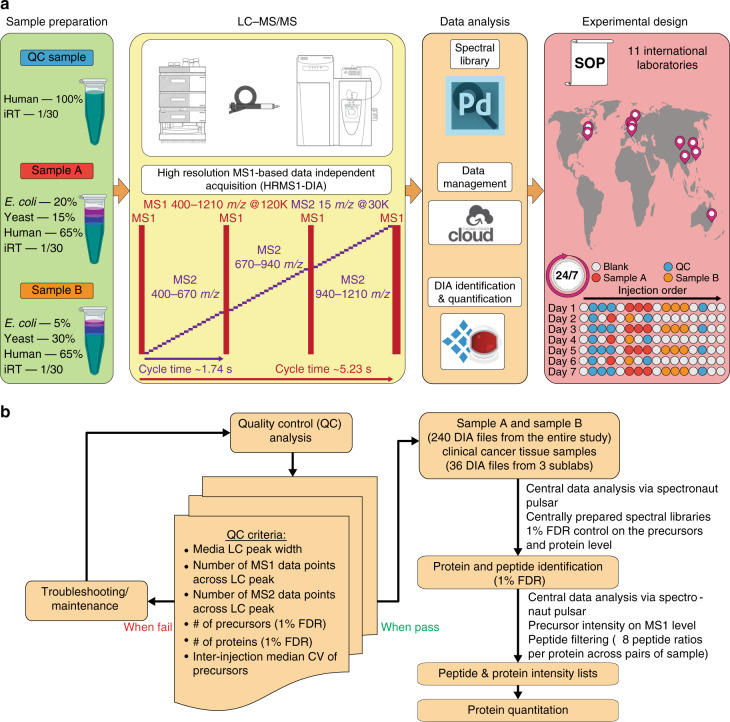
Data acquisition and analysis using a streamlined HRMS1-DIA workflow. **a** The quality control (QC) sample was a HeLa lysate digest. The controlled samples A and B were mixtures of HeLa, yeast, and *E. coli* lysates. The samples were analyzed by capillary LC-HRMS1-DIA on a Q Exactive HF system at eleven sites in a 24/7 mode for seven consecutive days. On days 1, 3, 5, and 7, all samples were run in three technical replicates; on days 2, 4, and 6, samples A and B were each run once, and the QC standard was run once before and after samples A and B. The rest of the time, the instruments were running blank injections. White circles are blank injections, blue circles are QC injections, red circles are Sample A injections, and orange circles are Sample B injections. **b** Quality control criteria were based on the evaluation of the performance of the capillary LC-HRMS1-DIA workflow before the 11 labs began the study (Supplementary Table 1). During the study, a QC standard was analyzed in three technical replicates on days 1, 3, 5, and 7. If the QC criteria were met, sample A and sample B data sets acquired on the same day were analyzed. If the QC standard analysis did not pass QC criteria, either instrument setup maintenance or troubleshooting were undertaken. In total, 240 DIA files from both sample A and sample B were centrally analyzed using Spectronaut (v11) with centrally generated spectral libraries. A criterion of 1% FDR was applied for identification at precursor and protein levels. The intensity of each identified peptide was exported to an.xls file, which was further processed via an R-script with the peptide-to-protein rollup pairwise ratio quantification strategy (see Methods section).

The HRMS1-DIA method used here features an original structure, involving multiple MS1 scans interspersed with 18 DIA MS/MS scans per scan cycle (in total 54 DIA MS/MS scans) (Fig. 1a). Quantification was based on precursor ion signals measured through high-resolution full MS scans with 120k resolution setting; the MS2 scans with 30k resolution setting was utilized for peptide identification only. High-resolution MS1-based peptide quantitation strategies, such as in conventional DDA or pSMART implementation of DIA, have demonstrated excellent quantitative performance^17,27^ and improved quantitation precision and dynamic range. In the HRMS1-DIA method employed here, the MS1 scan cycle rate (approximately every 1.7 s) was set independently of the MS2 cycle time. This decoupled scan event strategy assured that a sufficient number of MS1 scan events could be acquired over the median peptide chromatographic elution time to enable their precise quantification. By contrast, the MS2 acquisition parameters were set in a way to maximize peptide detection efficiency, through highly sensitive and selective measurement, rather than optimizing MS2 extracted ion chromatogram quantitation. Therefore, the overall MS2 cycle time was constrained by the need that each parent ion was sampled approximately three times within the duration of a typical chromatographic peak for identification purposes. The associated overall DIA MS2 cycle time of ~5.2 s resulted from constraints related to scan acquisition requirements at the given Orbitrap resolution setting, the maximum precursor ion injection time, and the precursor isolation window width. Briefly, the relatively high Orbitrap resolving power of 30 k together with the moderate precursor isolation window width of 15 Da directly enhance measurement selectivity while the maximum ion injection time, synchronized with the Orbitrap transient time of 64 ms such as to allow fully parallel ion collection and detection, maintains high measurement sensitivity. To systematically evaluate the reproducibility of the QC-benchmarked HRMS1-DIA workflow, spectral libraries were centrally constructed from the DDA analysis of high pH reverse phase fractions. The same capillary LC configuration and mobile phase gradient conditions were utilized for the HRMS1-DIA analysis and the DDA analysis. Spectronaut software (Biognosys) was applied for both individual onsite data analysis and central data analysis with the centrally prepared spectral libraries.

Our workflow represents a two-step procedure. First, the performance of the LC-MS platform operated with the HRMS1-DIA acquisition method was assessed to detect drifts from the predefined performance baseline and to trigger corrective measures to be taken if needed. A QC standard was analyzed using rigorous metrics to support system suitability testing; the QC standard was a commercially available peptide digest derived from the HeLa human cervical cancer cell line. Second, following successful platform qualification, the actual quantitative measurements of samples of interest were performed using the same acquisition method.

The baseline for the system suitability test was generated from the analyses of the QC standard performed by four reference laboratories in continuous operation mode over several days with LC-MS platforms operating at different levels of performance (Supplementary Table 1). These data enabled establishment of reference metrics and associated acceptance criteria for platform qualification from three replicate analyses of the QC standard. Reference metrics included median LC elution peak width, number of MS1 and MS2 data points across the LC elution peak, total precursor ions and protein groups identified, and inter-injection median CV on the precursor ion signals (Table 1). These metrics enabled real-time monitoring of platform status, covering both chromatographic and mass spectrometric performance characteristics. These QC acceptance criteria were also applied to identify possible issues decrementing analytical performance and credentialing the return to operational status upon completion of interventional maintenance.

**Table 1:** Reference metrics and associated acceptance criteria from the system suitability QC tests.

	Acceptance criteria
Median LC peak width	17 ± 3 s
Number of MS1 data points across LC peak	8–11
Number of MS2 data points across LC peak	3–4
Precursor IDs (1% FDR)	>48000
Protein IDs (1% FDR)	>5000
Inter-injection median CV of precursors	≤15%

### Performance evaluation of QC-benchmarked HRMS1-DIA workflow

The QC-benchmarked HRMS1-DIA method was used to analyze peptide digest mixtures of defined composition, referred to as controlled samples, by each of the 11 research laboratories in a 24/7 operation mode for seven consecutive days (Fig. 1a). The controlled samples were mixtures of digests prepared from diverse organisms: Sample A was 65% HeLa, 15% yeast, and 20% *E. coli* and Sample B was 65% HeLa, 30% yeast, and 5% *E. coli*. Each laboratory prepared their own controlled sample mixtures following standard operating procedures (Supplementary Notes 1–3). Each day, system suitability tests were performed using the QC standard, and no action was taken as long as QC acceptance criteria were satisfied (Fig. 1b). On days 1, 3, 5, and 7, the QC standard was analyzed in triplicate and then the controlled samples were analyzed in triplicate. At the end of each day, the QC standard was run. On days 2, 4, and 6, the QC standard was run once at the beginning and end of the day and the controlled samples were also analyzed once (Fig. 1b). At each laboratory, the files of data obtained on the QC standard were processed daily with Spectronaut Pulsar, enabling the extraction of QC metrics evaluated in system suitability tests (Supplementary Data 1). The QC files were searched against a spectral library constructed from DDA analyses of a peptide digest derived from human cell line KG1a.

**Fig. 2:**
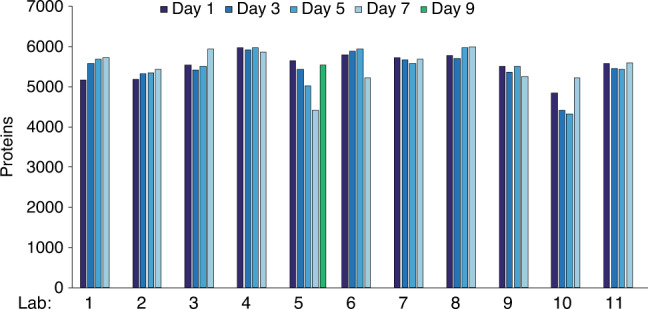
Quality control performance. The number of proteins identified (with a 1% FDR) from the HRMS1-DIA analyses of QC sample (2 µg of Hela digest on column) prior to analysis of the controlled samples at each laboratory was plotted over the 7-day evaluation period. Different days are indicated by shades of blue. The evaluation period was expanded to 9 days (green bar) for laboratory 5, which experienced technical issues on day 7 that were subsequently resolved. Source data are provided as a Source Data file.

The QC acceptance criteria were systematically satisfied for analyses performed by nine of the eleven laboratories, translating into the identification of 5028 to 5993 protein groups with 1% FDR (Fig. 2). One laboratory (Lab 10) faced significant analytical challenges, primarily due to poor chromatographic separations. Another participating laboratory (Lab 5) experienced technical issues on Day 7, translating into lower overall performance; specifically, only 4423 protein groups were identified. As the median LC peak elution width, the number of data points across the median LC peak elution at both MS1 and MS2 levels, and the inter-injection median CV on precursor ion signals were within the established criteria, the performance issues were not related to the chromatographic separation. Further investigation revealed the need for maintenance of the higher-energy collision dissociation cell of the mass spectrometer. After necessary maintenance on Day 8, the operation performance was validated on Day 9 and Lab 5 resumed analytical production. These challenges at Lab 5 and Lab 10 represent the only technical challenges encountered in the study. This demonstrates that the QC analysis employed here enables real-time monitoring of instrument status, identifies performance gaps, and provides a guide to root causes of performance issues, establishing a paradigm for both high-throughput and strict adherence to analytical performance needed for large cohort studies. Of the 11 participating laboratories, 10 were able to perform sample analyses on days 1, 3, 5, and 7, although Lab 5 continued on Day 9 instead of Day 7. Lab 10 did not participate in controlled sample analyses because they could not return to operational status within the time constraints of the study.

**Fig. 3:**
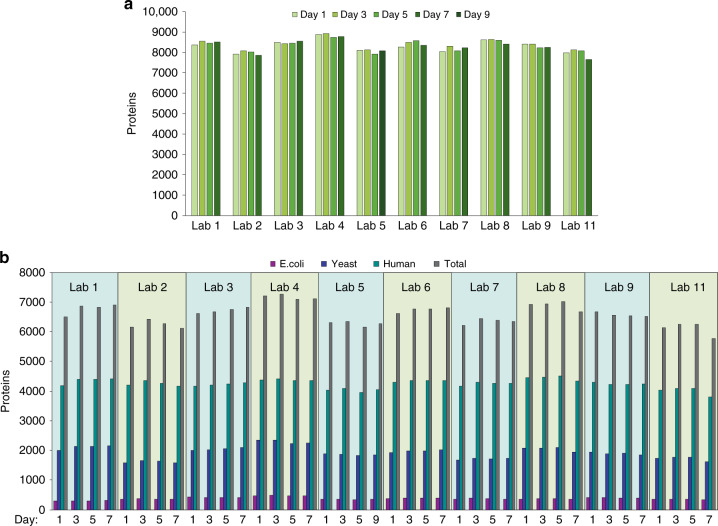
Overall performance of QC-benchmarked HRMS1-DIA workflow. **a** The number of proteins identified in both samples A and B (with a 1% FDR) at each laboratory was plotted over the 7-day evaluation period (indicated by shades of green). b The number of proteins quantified in total and for each individual organism in controlled samples (based on criteria described in the Methods section) at each laboratory is plotted over the 7-day evaluation period. Quantified proteins are shown in purple for E.coli, in blue for yeast, in green for human and in gray for total. For laboratory 5, which had an extended evaluation period of 9 days, due to technical issues detected, addressed, and resolved during day 7, the identification (**a**) and quantification (**b**) results obtained for day 9 substitutes those of day 7. Source data are provided as a Source Data file.

All data generated from Samples A and B were centrally processed in Spectronaut Pulsar. For peptide/protein identifications, the human spectral library used for QC standard data processing was supplemented with spectral libraries similarly constructed from yeast and *E. coli*. Peptide precursors were quantified using mass chromatogram areas extracted from MS1 data, and protein abundance changes were determined using a strategy in which a minimum of eight pairwise peptide ratios were combined across technical replicate injections for a given protein group (Supplementary Data 2). A total of 240 DIA injections of the controlled samples A and B were acquired at 10 research sites that met QC criteria, and more than 7600 protein groups were identified with 1% FDR (Fig. 3a). Approximately 4000 human proteins, 2000 yeast proteins, and 400 *E. coli* proteins (Fig. 3b) were quantified across the three injections per day over the four data acquisition days.

**Fig. 4:**
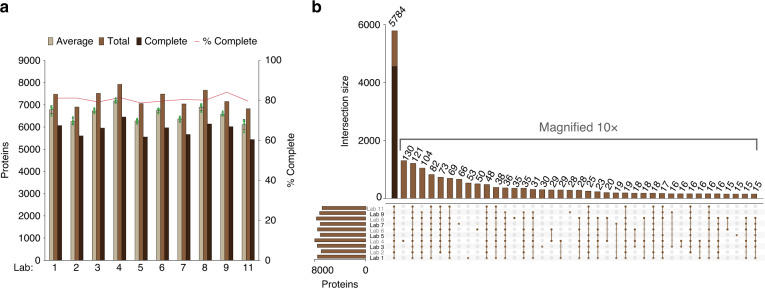
Reproducibility of quantitative proteome coverage achieved by HRMS-DIA analyses. **a** The bars depict the number of proteins quantified in controlled samples at each lab in total (brown), on average (light brown) and consistently (complete, dark brown) across the 7 days evaluation; bars representing average values are plotted together with individual data points in green (*n* = 4), corresponding to the four evaluation points sampled every 2 days, and error bars representing^+/−^ SD. The red line shows the percentage of complete profiles (Complete/Total). **b** The numbers of proteins co-quantified across various combinations of laboratories were plotted (vertical brown bars). The vertical dark brown bar (4565 proteins) overlapping the brown bar (5784 proteins) for the combination including all the laboratories reflects the number of proteins co-quantified by all contributing laboratories at every evaluation day. The horizontal brown bars reflect the number of proteins quantified in total at each laboratory. Source data are provided as a Source Data file.

The inter-day reproducibility was excellent at each site as more than 80% of the total number of locally quantified protein groups were quantified on each of the four data acquisition days. On average, more than 6500 protein groups were quantified with a relative standard deviation (RSD) <4% at each site (Fig. 4a). Furthermore, the inter-lab reproducibility was comparable with a total of 5784 protein groups quantified across the labs, representing ~80% of the proteins quantified locally. Notably, 4565 of these protein groups were not only quantified by all sites but were also quantified on each acquisition day (Fig. 4b).

**Fig. 5:**
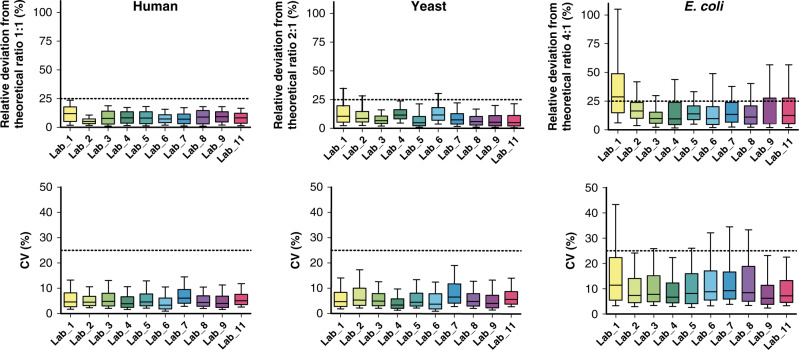
Quantitative performance metrics from analyses of the mixed proteome samples. The evaluation was based on the experimentally determined abundance changes of the systematically quantified proteins (every day by every laboratory) between controlled samples A and B (*n* = 4565) using three technical replicates. In the upper panels, the distribution of deviation from theoretical protein abundance changes for the three organisms (in %) was plotted for each laboratory through boxplots with center line, box bounds, and whiskers representing the median, 1st/3rd quartile, and 10th/90th percentile, respectively. Horizontal dashed lines were added at 25% relative deviation, as a reference value. In the lower panels, the distribution of the coefficients of variation obtained on the determined protein abundance changes for the three organisms across the various evaluation days (CV in %) was plotted for each laboratory through boxplots with center line, box bounds, and whiskers representing the median, 1st/3rd quartile, and 10th/90th percentile, respectively. Horizontal dashed lines were added at 25% CV, as a reference value. Source data are provided as a Source Data file.

In-depth evaluation of quantitative performance relied on the experimentally determined abundance differences between controlled samples A and B. The results demonstrated high quantification accuracy compared to theoretical abundance differences as reflected by the low deviation of experimental values from the theoretical values; the median values were generally lower than 10% deviation for human and yeast proteins (for a theoretical 1:1 and 2:1 abundance ratio, respectively) and typically lower than 20% deviation for *E. coli* proteins (for 4:1 abundance ratio, Fig. 5, upper panel). Deviations from theoretical values can arise from multiple factors associated either with variations in sample preparation or LC-MS performance (including LC retention time drift, platform-to-platform variation, or MS ionization efficiency). Due to the low relative stoichiometry of *E. coli* protein digest (i.e., 5% in sample B) added in the highly complex matrix (human and yeast proteome digests), a higher relative deviation is likely to be associated with the *E. coli* protein digest quantitative data than with the human or yeast. Indeed, one of the primary objectives of this study was to determine the efficacy of deploying standardized protocols to minimize these deviations among different laboratories. The low deviations from the theoretical values in this study demonstrates the high efficacy of the standardization approach. The highest deviation from theoretical values was observed in data collected by Lab 1, where the QC analysis passed the acceptance criteria and showed no evidence of reduced chromatographic or mass spectrometric performance. Therefore, the relatively higher deviation observed in data collected by Lab 1 compared to the other labs may have resulted from the samples themselves, and therefore from process error related to sample preparation at this lab. The analytical quantitative precision was excellent for the 10 participating laboratories, as illustrated by median CV values that were typically below 5% for the human and yeast proteins and below 10% for the *E. coli* proteins (Fig. 5, lower panel). Again, the slightly higher value obtained for the *E. coli* proteins likely resulted from the overall lower abundance of the *E. coli* proteome in the sample mixtures.

### Analyses of tumor tissue by the QC-benchmarked HRMS1-DIA workflow

**Fig. 6:**
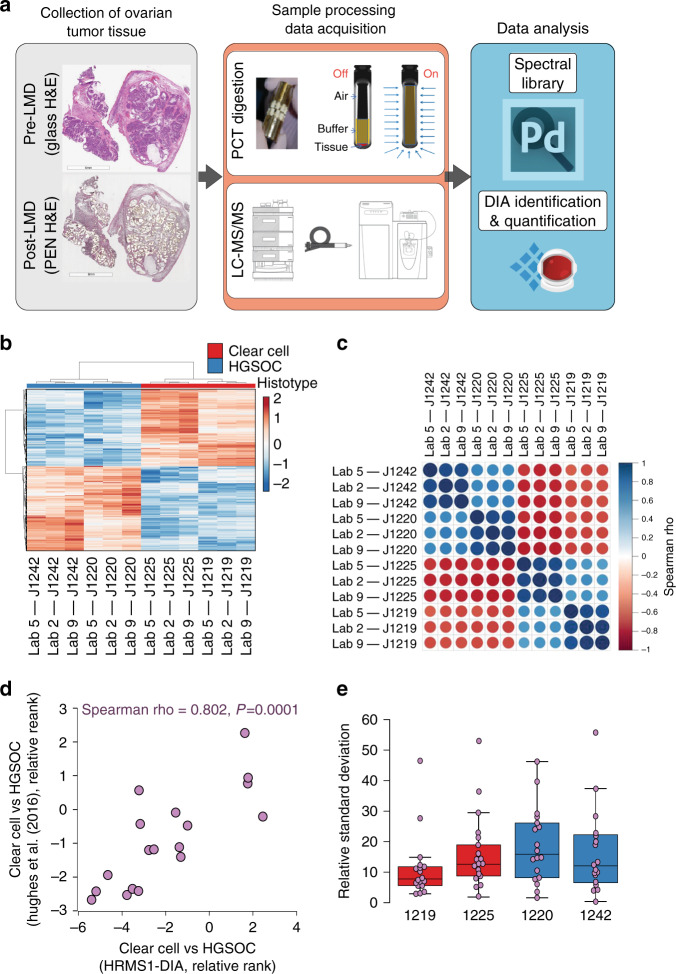
Archival ovarian cancer tissue sample analysis using HRMS1-DIA at three analytical sites. **a** Epithelial cancer cells were laser microdissected from formalin-fixed, paraffin-embedded (FFPE) archival ovarian clear cell carcinoma (OCCC, *n* = 2) and high-grade serous ovarian carcinoma (HGSOC, *n* = 2) tumor, digested by pressure-cycle technology, and evaluated by three international labs using the standardized HRMS1-DIA analytical workflow. Each biological sample was analyzed by HRMS1-DIA as three technical replicates. Quality control standards were evaluated before, between, and after the archival cancer tissue samples. The data acquisition was performed in a 24/7 operation mode. **b** Unsupervised cluster analysis of 394 proteins that were significantly altered (LIMMA adjusted *p* value < 0.01) between OCCC (red) and HGSOC (blue) tissues. **c** Correlation plot of proteins significantly altered across analytical sites and disease histotypes. **d** Correlation of proteins identified as altered in OCCC and HGSOC tissues in this study with a gene signature identified by Hughes et al.^32^. We co-quantified 18 significant protein alterations (LIMMA *p* value < 0.05) with this historic 112 gene signature stratifying OCCC and HGSOC ovarian cancers. Protein alterations were significantly correlated with this feature subset (Spearman = 0.802, *p* = 0.0001). **e** Quantitative reproducibility of 18 OCCC and HGSOC signature proteins across three analytical sites by patient tissue sample (*n* = 2 unique OCCC and *n* = 2 unique HGSOC patients). The figure shows the relative standard deviation between protein abundances obtained for the 18 signature proteins quantified at the three analytical sites. Boxplots reflect median RSD at midpoints as well as upper and lower limits of interquartile RSD range.

The standardized HRMS1-DIA analytical methodology was also used in a pilot-scale analysis of complex tissue digests prepared from ovarian cancer tumor tissue in three of the participating laboratories. Formalin-fixed, paraffin-embedded (FFPE) tissues, derived from two high-grade serous ovarian cancer (HGSOC) and two clear-cell ovarian cancer (OCCC) tumors, were thin sectioned onto polyethylene napthalate (PEN) membrane slides, and tumor cell populations were harvested by laser microdissection (LMD) (Fig. 6a). LMD-enriched epithelial cancer cell populations of interest were digested with trypsin using a pressure cycle technology workflow, and resulting peptides were analyzed in three technical replicates at each of three independent analytical laboratories using the QC-benchmarked, HRMS1-DIA method described above. Data were centrally processed and protein-level abundances were determined across disease histotypes as described above.

A total of 5712 unique protein groups were identified from all tumor samples and sites (Supplementary Data 3) and 3808 ± 343 (9.0% RSD) were co-quantified in individual patient tumor samples that further exhibited high quantitative correlation across analytical sites (Spearman Rho = 0.62 ± 0.08, *p* < 0.0001). Differential analyses revealed 394 significantly altered proteins (LIMMA adjusted *P* value < 0.01) between OCCC and HGSOC patient tissues (Supplementary Data 4; Fig. 6b) and these features exhibited high quantitative correlation in individual patient samples across analytical sites (Spearman Rho = 0.92 ± 0.02) and disease histotypes (Spearman Rho = 0.63 ± 0.04) (Fig. 6c). Pathway analyses of these alterations revealed activation of peroxisome proliferator-activated receptor alpha (PPAR-alpha)/ Retinoid X receptor alpha (RXR-alpha) as well as cyclin-dependent kinase 5 (CDK5) signaling, but inhibition of metabolic pathways, namely Oxidative Phosphorylation, in OCCC vs HGSOC patient tumor tissues (Supplementary Data 5). Notably, PPAR-alpha has been identified as a possible therapeutic target for the treatment of clear cell renal cell carcinoma (ccRCC)^28^, a cancer type that is molecularly and morphologically similar to OCCC^29^ and one in which CDK5 signaling has also been identified as an important regulatory pathway^29^. The activation of oxidative phosphorylation in HGSOC vs OCCC cancers is consistent with previous reports of the dependency of HGSOC tumor cells on this mode of energy metabolism^30^. We further compared the protein alteration quantified here with a protein signature previously reported to stratify HGSOC and OCCC^31,32^, and 18 co-quantified proteins were found to exhibit concordant protein abundances (*R* = 0.802, *P* = 0.0001) (Fig. 6d). Quantitative reproducibility of these 18 HGSOC and OCCC signature proteins across three analytical sites for each patient tissue sample revealed a mean RSD of 15.45% with a standard error of ± 1.43 (Supplementary Data 5; Fig. 6e).

## Discussion

A standardized analytical approach to support the creation of digital proteogenomic biobanks of clinical biospecimens for prospective and retrospective data analyses has been designed. To realize this goal, developing a standardized and QC-benchmarked workflow was necessary. The approach developed here for generation of harmonized datasets will maximize the consumption and (re)usability of downstream data sets and empower team-driven science initiatives and population-level insights between the proteome and human disease. Our multi-site, international study defined a QC-driven, HRMS1-DIA workflow with excellent analytical reproducibility and label-free quantitative performance providing deep global proteotype profiling across diverse sample types, including a pilot analyses of clinically-derived, archival cancer tissue samples. The workflow resulted in the consistent and confident identification of more than 5000 proteins in the peptide digest derived from a human cell line that served as our QC-benchmark standard. Applied to the analysis of the complex mixture of digests from human, yeast, and bacterial cells, it allowed identification, on average, of more than 7600 proteins and the quantitation of more than 6500 proteins from ten of the eleven laboratories that participated.

We provided further proof of concept by applying this workflow to tryptic digests established from real-world archival FFPE cancer tissue specimens. Highly reliable protein quantitation enabled the detection of disease histotype-specific protein alterations in each of the three laboratories that took part in this analysis. The consistent depth of proteome coverage (>80% of total proteins quantified across partnering sites) and analytical performance achieved across diverse sample types and sites using a 1-h, capillary LC-MS method operating in a 24/7 mode demonstrates that this high-throughput and robust workflow, enabling the quantification of more than 100 proteins per minute, is ready for application to large cohort tissue proteomic studies distributed across international centers. This workflow defines and implements QC benchmark expectations that can be monitored in real-time during primary DIA data production to ensure that data quality standards are achieved and that can be leveraged during downstream data processing to assess and track analytical variability and bias in cohort-level data. The presented HRMS1-DIA workflow is a standardized, quantitative method that is driven by defined QC expectations and that exhibits stable, highly reproducible, and scalable performance to support both basic discovery proteomics research and population-scale clinical sample analyses in a high-throughput manner.

## Methods

### Quality control standard sample preparation

The QC standard was a commercially available peptide digest derived from the HeLa human cervical cancer cell line, the Pierce HeLa Protein Digest Standard (Thermo Fisher Scientific), resuspended at a concentration of 1 µg µL^−1^ in HPLC-grade water containing 0.1% (v/v) formic acid and supplemented with eleven non-naturally occurring synthetic peptides from the iRT kit (Biognosys) at a ratio 1:30 v/v. The detailed preparation instructions are described in Supplementary Note 1.

### Mixed proteome sample preparation

The controlled samples A and B were prepared from the Pierce HeLa Protein Digest Standard (Thermo Fisher Scientific), the Mass Spec-Compatible Yeast Digest (Promega), and the MassPREP *E. coli* Digest Standard (Waters). Each digest was resuspended at a concentration of 1 µg µL^−1^ with HPLC-grade water containing 0.1% (v/v) formic acid. Sample A was prepared by mixing human, yeast, and *E. coli* protein digests at 65%, 15%, and 20% w/w, respectively. Sample B was prepared by mixing human, yeast, and *E. coli* protein digests at 65%, 30%, and 5% w/w, respectively. The iRT kit (Biognosys) was added to each of the controlled samples at a ratio 1:30 v/v. For analysis, 2 µl of each sample was injected. The detailed preparation instructions are described in Supplementary Note 1.

### Cancer tissue specimen sample preparation

Four FFPE ovarian cancer surgical tissue specimens (two OCCC and two HGSOC) [with patient consent and approved for use under Western IRB-approved protocol “An Integrated Molecular Analysis of Endometrial and Ovarian Cancer to Identify and Validate Clinically Informative Biomarkers” deemed exempt under US Federal regulation 45 CFR 46.102(f)] were selected for laser microdissection. Thin (8 µm) tissue sections were cut using a microtome and placed on polyethylene naphthalate membrane slides (Leica Microsystems). After staining with aqueous hematoxylin and eosin, laser capture microdissection (Leica LMD7) was used to harvest tumor cells from thin sections, which were collected by gravity into microcentrifuge tubes containing 45 µL of LC-MS grade water (mean tumor cell area captured(87.03 ± 1.93 mm^2^)). Tissue samples were vacuum dried and transferred into MicroTubes (Pressure BioSciences, Inc., Medford, MA) containing 20 microliters of 100 mM TEAB/10% acetonitrile (ACN) and incubated at 99 °C for 30 min. The temperature was lowered to 50 °C, and SMART trypsin (ThermoFisher Scientific Inc.) was added at a ratio of 1 mg per 30,000,000 µm^2^ tissue. Tubes were capped with MicroPestles (Pressure BioSciences, Inc.) and pressure-assisted digestion was performed in a 2320EXT barocycler (Pressure BioSciences, Inc.) by sequentially cycling between 45 kpsi (50 s dwell time) and atmospheric pressure (10 s dwell time) for 60 cycles at 50 °C. The peptide digests were transferred to 0.5-mL microcentrifuge tubes, vacuum dried, and purified using Pierce C_18_ Spin Columns. Resulting peptides were resuspended in 25 mM ammonium bicarbonate, pH 8.0 and the peptide concentration of each digest was determined using the bicinchoninic acid (BCA) assay (Pierce Biotechnology); the peptide yield was 0.68 ± 0.08 µg mm^−2^ tissue collected.

### Cell culture and lysis

Human KG1a cells (ATCC^®^ CCL-246.1™) were maintained and propagated in RPMI 1640 medium supplemented with 10% FBS and 1% penicillin/streptomycin at 37 °C in a 5% CO_2_ atmosphere, and cells were lysed in buffer containing 4% SDS. Lysates were sonicated (Ningbo Scientz) and centrifuged at 15,000 × g for 15 min. The protein concentration of the supernatant was determined by using the BCA assay kit. Proteins (200 µg) were digested overnight with Lys-C (Wako Chemicals) and trypsin (Promega) using the filter-aided sample preparation protocol^33^. Peptides were recovered and desalted using Oasis HLB 1-cc cartridges (Waters Corp.). In brief, peptides were loaded onto a HLB cartridge, washed with 0.1% trifluoroacetic acid (TFA), and eluted with 30% ACN and 60% ACN in 0.1% TFA. Flow through from the Oasis HLB was then loaded onto a Sep Pak C18 cartridge (Waters), washed with 0.1% TFA, and then eluted with 30% ACN and 60% ACN in 0.1% TFA. Eluates from HLB and C18 cartridges were combined and lyophilized in a vacuum centrifuge for LC-MS/MS proteome analysis as described below. *E. coli* cells were incubated at 37 °C with 200 rpm shaking and harvested at mid-log phase. Cells were pelleted at 10,000 × g, and then washed three times with phosphate-buffered saline pH 7.5. Harvested cell pellets were digested using the methods mentioned above.

### High-pH reversed-phase liquid chromatography fractionation

A total of 200 µg of in-house-prepared human and *E. coli* digests and Promega yeast protein digest were each fractionated by offline reversed-phase LC (Waters Acquity UPLC Peptide BEH300 C_18_ 1.7 µm, 1 mm i.d. × 150 mm column employing an Ultimate 3000 HPLC, Thermo Fisher Scientific) operating with column compartment temperature of 60 °C and flow rate of 100 µL min^−1^. Mobile phase A consisted of 10 mM ammonium hydroxide, and mobile phase B consisted of 10 mM ammonium hydroxide/90% ACN. Sample loading and peptide separation were performed by applying a mixture of mobile phases as follows: (i) 1% mobile phase B for 4 min, (ii) 1% to 6% mobile phase B in 6 min, (iii) 6% to 30% mobile phase B in 22 min, (iv) 30% to 60% mobile phase B in 5 min, and (v) ramp to 95% mobile phase B in 3 min. The washing step at 95% mobile phase B lasted for 2 min and was followed by an equilibration step at 1% mobile phase B for 5 min. Fractions were collected between 4 and 40 min; 36 fractions were collected for the human digest and 12 fractions were collected for yeast and *E.coli* protein digests. Each fraction was evaporated to dryness and resuspended by adding 10 µL or 30 µL for human and for yeast or *E. coli*, respectively, of an aqueous solution containing 0.1% (v/v) formic acid supplemented with the iRT kit (Biognosys) at a ratio 1:10 v/v.

To generate the cancer tissue-specific spectral library, 30 µg from each patient sample digest were combined (for a total of 120 µg) and fractionated by high pH reversed-phase liquid chromatography into 96 fractions using a linear gradient of ACN (0.69% per minute) as described above. Concatenated fractions were pooled into 36 fractions, lyophilized, and resuspended in 0.1% formic acid for analysis.

### Spectral library generation by data-dependent analysis

HRMS1-DIA data sets were acquired on the Q Exactive HF MS instrument platform at the eleven laboratories. The spectral libraries were generated centrally via DDA of a fractionated mixed proteome sample and cancer tissue samples on either a Q Exactive HF or an Orbitrap Lumos mass spectrometer. The same capillary LC configuration and mobile phase gradient elution conditions as for HRMS1-DIA were applied.

Each resuspended fraction of the mixed proteome protein digest and the cancer tissue-specific spectral library was analyzed with an Easy-nLC 1200 (Thermo Fisher Scientific) coupled to Orbitrap Fusion Lumos or Q Exactive HF-X mass spectrometers (Thermo Fisher Scientific) operated with DDA methods; 2 µL of each fraction was injected. Peptide separations were carried out on an Acclaim PepMap RSLC C_18_, 2 µm, 100 Å, 150 µm i.d. x 150 mm, nanoViper EASY-Spray column (Thermo Fisher Scientific). The column temperature was maintained at 50 °C using the EASY-Spray oven. Mobile phase A consisted of HPLC-grade water with 0.1% (v/v) formic acid, and mobile phase B consisted of HPLC-grade ACN with 20% (v/v) HPLC-grade water and 0.1% (v/v) formic acid. Samples were loaded at 3 µL min^−1^ with 100% mobile phase A for 2 min. Peptide elution was performed at 1.2 µL min^−1^ using the following gradient: i) 3% to 8% mobile phase B in 4 min, ii) 8% to 25% mobile phase B in 50 min, and iii) ramp to 80% mobile phase B in 4 min. The washing step at 80% mobile phase B lasted 2 min and was followed by an equilibration step at 100% A (1.7 min at 3 µL min^−1^).

The Orbitrap Fusion Lumos mass spectrometer was configured for DDA using the full MS-data-dependent MS/MS setup and was operated in positive polarity mode. Spray voltage was set at 2 kV, funnel RF level at 40, and capillary temperature at 250 °C. Full MS survey scans were acquired at a resolution of 60,000 with an automatic gain control (AGC) target value of 4e5 and a maximum injection time of 20 ms over a scan range of *m/z* 350-1500. A data-dependent top 40 method was used during which up to 40 precursor ions were selected from each full MS scan to be fragmented through higher energy collisional dissociation (HCD). HCD MS/MS scans were acquired with a normalized collision energy of 30 at a resolution of 15,000 and with a starting mass of *m/z* 130. Precursor ions were isolated in a 1.6-Th window and accumulated to reach an AGC target value of 5e4 with a maximum injection time of 30 ms. Precursor ions with a charge state between 2 and 7 were selected for fragmentation, and the monoisotopic peak was isolated. Precursor ions and their isotopes selected for fragmentation were dynamically excluded for 30 s.

The Q Exactive HF-X mass spectrometer was configured for DDA using the full MS-data-dependent MS/MS setup and was operated in positive polarity mode. Spray voltage was set at 2 kV, funnel RF level at 40, and capillary temperature at 250 °C. Full MS survey scans were acquired at a resolution of 60,000 with an AGC target value of 3e6, a maximum injection time -of 20 ms, and a scan range of *m/z* 350-1500. A data-dependent top 20 method was used during which up to 20 precursor ions were selected from each full MS scan to be fragmented through HCD. HCD MS/MS scans were acquired with normalized collision energy 27 at a resolution of 15,000 with a starting mass of *m/z* 120. Precursor ions were isolated in a 1.6-Th window and accumulated to reach an AGC target value of 5e4 with a maximum injection time of 45 ms. Precursor ions with a charge state higher than 1 were selected for fragmentation, and the monoisotopic peak was isolated. Precursor ions and their isotopes selected for fragmentation were dynamically excluded for 40 s.

### Data independent analyses of mixed proteomes

QC standards and Samples A and B were analyzed as technical triplicates with an Easy-nLC 1200 or an Ultimate 3000 RSLC equipped with capillary flow meter coupled to a Q Exactive HF mass spectrometer (LC instrument platform at each laboratory is detailed in Supplementary Table 2) operated with DIA methods. Method settings are provided in Supplementary Notes 2 and 3.

Peptide separations were carried out on an Acclaim PepMap RSLC C_18_, 2 µm, 100 Å, 150 µm i.d. × 150 mm, nanoViper EASY-Spray column (Thermo Fisher Scientific). The column temperature was maintained at 50 °C using the EASY-Spray oven. Mobile phase A consisted of HPLC-grade water with 0.1% (v/v) formic acid, and mobile phase B consisted of HPLC-grade ACN with 20% (v/v) HPLC-grade water and 0.1% (v/v) formic acid.

For LC-MS/MS analyses performed on the Easy-nLC 1200, samples were loaded at 4 µL min^−1^ with 100% mobile phase A for 5 min. Peptide elution was performed using the following gradient: (i) 2% to 8% mobile phase B in 4 min, (ii) 8% to 32% mobile phase B in 49 min, (iii) 32% to 60 % mobile phase B in 1 min, and iv) ramp to 98% mobile phase B in 1 min at 2 µL min^−1^. The washing step at 98% mobile phase B lasted 10 min (at 2 µL min^−1^) and was followed by an equilibration step at 100% mobile phase A (6.7 min at 3 µL min^−1^). After sample injection, the autosampler was washed by three cycles of drawing/dispensing 22 µL of HPLC-grade ACN containing 20% (v/v) HPLC-grade water and 0.1% (v/v) formic acid, followed by three cycles of drawing/dispensing 22 µL of HPLC-grade water containing 0.1% (v/v) formic acid.

For LC-MS/MS analyses performed on the Ultimate 3000 RSLC, samples were loaded at 3 µL min^−1^ with 100% mobile phase A for 5 min. Peptide elution was using the following gradient: (i) 2% to 8% mobile phase B in 4 min, (ii) 8% to 32% mobile phase B in 49 min, (iii) 32% to 60% mobile phase B in 1 min, and (iv) ramp to 98% mobile phase B in 1 min at 3 µL min^−1^. After 5 min of run time, the inject valve was switched to the load position, and the autosampler procedure was triggered at 8.1 min. This allowed the injection loop to be washed and filled with a 20 µL plug of HPLC-grade ACN containing 20% (v/v) HPLC-grade water and 0.1% (v/v) formic acid. At 60 min, the injection valve was switched back to the inject position, allowing the 20 µL plug of ACN contained in the injection loop to be delivered quickly to the column for thorough washing. Concurrently (i.e., at 60 min) the pump settings were modified to deliver 100% mobile phase A in the flow path for 13 min at 3 µL min^−1^, which is sufficient time to achieve equilibration.

The Q Exactive HF mass spectrometer was configured for DIA by combining two experiment elements, corresponding to a full MS experiment and an MS/MS experiment, and was operated in positive polarity mode. Spray voltage was set in the range of 2–2.4 kV to sustain a stable spray, funnel RF level was set at 50, and capillary temperature was maintained at 250 °C. The full MS experiment included one broadband scan acquired over *m/z* 400–1210 at a resolution of 120,000 with an AGC target value of 3e6 and with maximum injection time of 50 ms. The MS/MS experiment included 18 scans/cycle (for a total of 54 scans) acquired at *R* = 30,000 with an AGC target value of 1e6 and with Auto maximum injection time. The precursor ions were isolated within a 15 Da window and fragmented by HCD acquired with normalized collision energy 28 and default charge state 3 and with a starting *m/z* of 200. Center values of isolation windows are reported in Supplementary Notes 2 and 3.

### Search of DDA data and spectral library generation

The assignment of MS/MS spectra generated from DDA analyses of the Mixed Proteome protein digests was made with Proteome Discoverer 2.2 software and Sequest HT algorithm using UniProt database filtered for *Homo sapiens* (downloaded April 2016), *Saccharomyces cerevisiae* (downloaded May 2016), or *Escherichia coli* (downloaded February 2016) taxonomies, concatenated with iRT peptide.fasta file (downloaded from the Biognosys webpage). Tolerances on precursors and fragment ions were set at +/−10 ppm and +/−0.02 Da, respectively. The searches were performed by specifying Trypsin (full) enzyme digest specificity constraints with a maximum of two missed cleavage sites allowed, Oxidation as dynamic modification, and Carbamidomethylation as static modification. The data were also searched against a decoy database, and the results were used to estimate q values using the Percolator algorithm within the Proteome Discoverer version 2.2 suite. Protein and peptide identifications were filtered at a false discovery rate (FDR) <1% with no threshold on the minimum number of peptides. Proteome discoverer result files were imported into Spectronaut Pulsar 11.0.15038.23.24843 (Asimov) software for the generation of the spectral libraries (.kit files) for each organism using default settings.

### Data independent analysis bioinformatics and protein-level roll-up

A step-by-step procedure for data processing and evaluation with Spectronaut software is reported in Supplementary Note 4. Briefly, raw files (including QC standard and Sample A and Sample B raw data) were imported into Spectronaut software and searched against pertinent spectral libraries. The data generated by the acquisition method combining MS1 scans with interspersed DIA MS2 scans were directly processed by Spectronaut without conversion or pre-definition of the actual method structure as input information in the software. Both MS1 and MS2 data were used for peptide identification while the parameters of the quantification process were solely derived from the MS1 data. The extraction of data used dynamic MS1 and MS2 mass tolerances, dynamic window for extracted ion current extraction window, and a non-linear iRT calibration strategy. The identification was carried out using a kernel density estimator and FDR cut-off of 0.01 at precursor and protein levels. The extracted quantitative data benefited from interference correction and a local cross-run normalization strategy. The data processing results were exported using two customized reports for peptide and protein identification and for further quantification processing using R scripts (Supplementary Software). The peptide customized report included EG.PrecursorId, PG.ProteinAccessions, PG.ProteinDescriptions, EG.Qvalue, PEP.Quantity, and PG.Quantity fields. The protein customized report included PG.FastaFiles, PG.ProteinGroups, and PG.Quantity fields. The scripts were applied to subsets of analyses, which were grouped together according to day and lab. Through these scripts, additional filtering steps were applied prior to the comparison of quantifications of proteins between samples A and B including the removal of peptides shared by different organisms and the removal of sub-optimal precursor ions of peptides detected under different charge states (i.e., the precursor ions not showing the highest number of retained quantitative data across the series of analyses considered). For each retained peptide, the maximum number of inter-sample combinations between the replicated analyses with quantitative data available was determined. This estimation was expanded at the protein level by summing these numbers obtained for all surrogate peptides. The proteins were considered as reliably quantifiable in the experiment and retained for further processing when at least eight combinations were available. The actual relative quantification of retained proteins was performed by calculating the geometric median of the peptide pairwise ratios obtained from all inter-sample combinations.

### Data independent analyses of ovarian cancer samples

Equivalent amounts of tissue digests were shipped to three analytical laboratories, (Lab 2, Lab 5, and Lab 9). Samples were resuspended to final concentrations of 1 µg µL^−1^ in 0.1% formic acid with iRT and analyzed in triplicate on a Q Exactive HF using the HRMS1-DIA method as described above. The Q Exactive HF mass spectrometer was therefore configured for DIA by combining two experiment elements, corresponding to a full MS experiment and an MS/MS experiment, and was operated in positive polarity mode. Spray voltage was set in the range of 2–2.4 kV to sustain a stable spray, funnel RF level was set at 50, and capillary temperature was maintained at 250 °C. The full MS experiment included one broadband scan acquired over *m/z* 400–1210 at a resolution of 120,000 with an AGC target value of 3e6 and with maximum injection time of 50 ms. The MS/MS experiment included 18 scans/cycle (for a total of 54 scans) acquired at *R* = 30,000 with an AGC target value of 1e6 and with Auto maximum injection time. The MS1 scan rate required ~1.7 s leading to an overall MS1 to MS1 scan time of ~5.2 s, which equates to the acquisition of ~8−11 MS1 data points across the median LC peptide elution peak width (17 ± 3 s) in this study. The precursor ions were isolated within a 15 Da window and fragmented by HCD acquired with normalized collision energy 28 and default charge state 3 and with a starting *m/z* of 200. Center values of isolation windows are reported in Supplementary Notes 2 and 3. QC standards were analyzed in triplicate before and after cancer tissue sample analyses and a single QC standard analysis was performed midway through the overall analysis.

Protein abundance was determined for patient sample-specific HRMS1-DIA data collected by each analytical site. The proteins retained to undergo comparative analyses were selected using the peptide filtering strategy described above and by requiring that the sum of observations of all retained peptides for each of these proteins had to satisfy a minimum number of 3 across replicated analyses at the individual patient sample level. The abundance of retained proteins was estimated from each analysis by summing the intensities of individual peptides and averaging them across triplicates at each analytical site. Log_2_ fold-change protein abundances reflective of summed protein abundance ratios were calculated relative to average protein abundances quantified across all samples for a given protein group. Differential analysis was performed using the LIMMA package (version 3.8, https://bioconductor.org/packages/release/bioc/html/limma.html) in R (version 3.5.2) with the expectation that proteins significantly altered between HGSOC and OCCC patient tissues exhibited a minimum LIMMA *p* value < 0.05 and cluster analyses was performed using ClustVis (https://biit.cs.ut.ee/clustvis/). Pathway analysis of protein alterations was performed using Ingenuity Pathway Analysis (Qiagen). Correlation analyses of proteins significantly altered between HGSOC and OCCC patient tissues was performed using Corrplot (version 0.84, https://cran.r-project.org/web/packages/corrplot/vignettes/corrplot-intro.html) in R (version 3.5.2). Spearman rho was calculated with features co-altered relative to a 112 protein and gene signature for frozen tissue stratifying high-grade serous and clear-cell ovarian cancers^29^, and box plots of relative standard deviation calculated from summed protein abundances corresponding to eighteen HGSOC/OCCC signature proteins of interest were performed using MedCalc (version 19.0.3).

### Statistics and reproducibility

All samples discussed in this manuscript (QC standards, controlled samples A and B and ovarian cancer histotypes) were measured and analyzed as technical triplicates.

### Reporting summary

Further information on research design is available in the Nature Research Reporting Summary linked to this article.

## Supplementary information

Supplementary Information

Peer Review File

Description of Additional Supplementary Files

Supplementary Data 1

Supplementary Data 2

Supplementary Data 3

Supplementary Data 4

Supplementary Data 5

Supplementary Software

Reporting Summary

## Data Availability

The mass spectrometry proteomics data (.raw files) and spectral libraries used for the data processing (.kit files) have been deposited to the ProteomeXchange Consortium via the MassIVE partner repository (https://massive.ucsd.edu) with the dataset identifier MSV000084976. Source data are provided with this paper.

## Source data

Source Data

## Code availability

The R package used for peptides to proteins rollup and quantification is provided as Supplementary Software.
