# Supplementary material for: Standardization and harmonization of distributed multi-center proteotype analysis supporting precision medicine studies
Source: Nat Commun. 2020 Oct 16;11:5248. doi: 10.1038/s41467-020-18904-9 (PMC7568553; doi:10.1038/s41467-020-18904-9)
Supplement: Supplementary file 1 — Supplementary Information [file 41467_2020_18904_MOESM1_ESM.pdf]

Supplementary Information for:

Standardization and Harmonization of Distributed Multi-Center Proteotype Analysis Supporting Precision Medicine Studies

Xuan et al.

Supplementary Table 1: The QC standard performed by four reference laboratories

	Thermo Lab Hemel	Thermo Lab New Jersey	Thermo Lab Shanghai	Thermo Lab Basel
Median LC peak width	14.4 s	14.8 s	18 s	15 s
Number of MS1 data points across LC peak	8	8	9	8
Number of MS2 data points across LC peak	3	3	3	3
Precursor IDs (1% FDR)	67507	63550	64302	63955
Protein IDs (1% FDR)	5504	5450	5470	5600
Inter-injection median CV of precursors	8.4%	6.8%	6.5%	10.3%

Supplementary Table 2: LC instrument platform at each laboratory

Laboratory	LC system
Lab_1	Ultimate 3000 RSLC
Lab_2	Easy-nLC 1200
Lab_3	Ultimate 3000 RSLC
Lab_4	Ultimate 3000 RSLC
Lab_5	Easy-nLC 1200
Lab_6	Ultimate 3000 RSLC
Lab_7	Easy-nLC 1200
Lab_8	Ultimate 3000 RSLC
Lab_9	Ultimate 3000 RSLC
Lab_10	Easy-nLC 1200
Lab_11	Ultimate 3000 RSLC

Supplementary Note 1: Standard operating procedure - Sample preparation

---

1) Material

Description	Source	Product Number
Pierce HeLa Protein Digest Standard	Thermo Scientific™	88329
MassPREP E. coli Digest Standard	Waters	186003196
Mass Spec-Compatible Yeast Digest	Promega	V7461
iRT-standard	Biognosys	Ki-3002-2
0.1% FA in Water, OPTIMA LC/MS	Fisher Chemicals	LS118-500
*Protein LoBind Tubes, 0.5 mL, PCR clean, 100 tubes	Eppendorf	30108094
*Microvials PP, 0.3ml with short thread	VWR	548-0440
*Screw cap PP blue 9mm	VWR	548-0088

*Recommended tubes and LC vials (and caps). Other low binding tubes and vials can be used as well.

2) Mixtures preparation

Stock solutions

iRT mixture (stock solution) (based on supplier instructions)
- Add 50 µL dissolution buffer (blue cap) to the iRT standard tube (red cap)
- Vortex the iRT standard tube for at least 1 minute.
- Subject tube to 5 min ultrasonic bath
- Store iRT mixture (stock solution) at 2-8°C (stable for 12 weeks)

HeLa digest stock solution (1 µg/µ - 100 µL)
- Defrost 10 vials of 20 µg of Hela digest (88329, lyophilized) at room temperature for 15 min
- Add 20 µL H_20 (+0.1% HCOOH) to each vial
- Vortex each vial for at least 1 minute

- Subject each vial to 5 min ultrasonic bath
- Vortex each vial for at least 30 s
- Keep one vial to prepare stock solution and transfer the content of 9 other vials in it (200 µL in total)

Yeast digest stock solution (1 µg/µ - 100 µL)

- Defrost 1 vial of 100 µg Yeast digest (V7461, lyophilized) at room temperature for 15 min
- Add 100 µL H_20 (+0.1% HCOOH) to the vial
- Vortex the vial for at least 1 minute
- Subject the vial to 5 min ultrasonic bath
- Vortex the vial for at least 30 s
- Aliquot the stock solution (concentration 1µg/µL) into 50 µL volumes (0.5 mL low binding tubes) and freeze for future use.

E. coli digest stock solution (1 µg/µ - 100 µL)

- Defrost 1 vial of 100 µg E. coli digest (186003196, lyophilized) at room temperature for 15 min
- Add 100 µL H_20 (+0.1% HCOOH) to the vial
- Vortex the vial for at least 1 minute
- Subject the vial to 5 min ultrasonic bath
- Vortex the vial for at least 30 s
- Aliquot the stock solution (concentration 1µg/µL) into 25 µL volumes (0.5 mL low binding tubes) and freeze for future use.

Multiple sites samples

Mix A (1 µg/µL - 80 µL)

- Transfer 52 µL from Hela digest stock solution at 1µg/µL to a LC vial
- Transfer 12 µL from Yeast digest stock solution at 1µg/µL to the LC vial
- Transfer 16 µL from E. coli digest stock solution at 1µg/µL to the LC vial
- Transfer 2.5 µL from iRT mixture (stock solution) to the LC vial
- Vortex to mix
- Put LC vial in autosampler. To be injected: 2µL/analysis (at least 30 µL needed for the study)

Mix B (1 µg/µL - 80 µL)

- Transfer 52 µL from Hela digest stock solution at 1µg/µL to a LC vial
- Transfer 24 µL from Yeast digest stock solution at 1µg/µL to the LC vial
- Transfer 4 µL from E. coli digest stock solution at 1µg/µL to the LC vial
- Transfer 2.5 µL from iRT mixture (stock solution) to the LC vial
- Vortex to mix
- Put LC vial in autosampler. To be injected: 2µL/analysis (at least 30 µL needed for the study)

QC sample (1 µg/µL – 80-96 µL)

- Transfer the remaining content (should be between 80 and 96 µL) from Hela digest stock solution at 1µg/µL to a LC vial
- Transfer 3 µL from iRT mixture (stock solution) to the LC vial
- Vortex to mix
- Put LC vial in autosampler. To be injected: 2µL/analysis (at least 44 µL needed for the study)

Blank

- Transfer 300 µL H_2O (+0.1% HCOOH) to a LC vial
- Put LC vial in autosampler. To be injected: 2µL/analysis (at least 160 µL needed for the study)

3) Samples analyses plan

Replicates	Day 1	Day 2	Day 3	Day 4	Day 5	Day 6	Day 7
Blank	1	1	1	1	1	1	1
QC sample	3	1	3	1	3	1	3
Blank	1	1	1	1	1	1	1
Sample A	3	1	3	1	3	1	3
Blank	1	1	1	1	1	1	1
Sample B	3	1	3	1	3	1	3
Blank	1	1	1	1	1	1	1
QC sample	1	1	1	1	1	1	1
Blank (for 24 h total instrument time)	4 (up to)	10 (up to)	4 (up to)	10 (up to)	4 (up to)	10 (up to)	4 (up to)

Supplementary Note 2: Standard operating procedure - DIA Analysis with Capillary-flow UltiMate 3000 RSLC

1) Hardware configuration

- Mass Spectrometer: Q Exactive HF.

- Layout of Chromatographic System: UltiMate 3000 RSLCnano with capillary flow meter operated in a one-column setup.

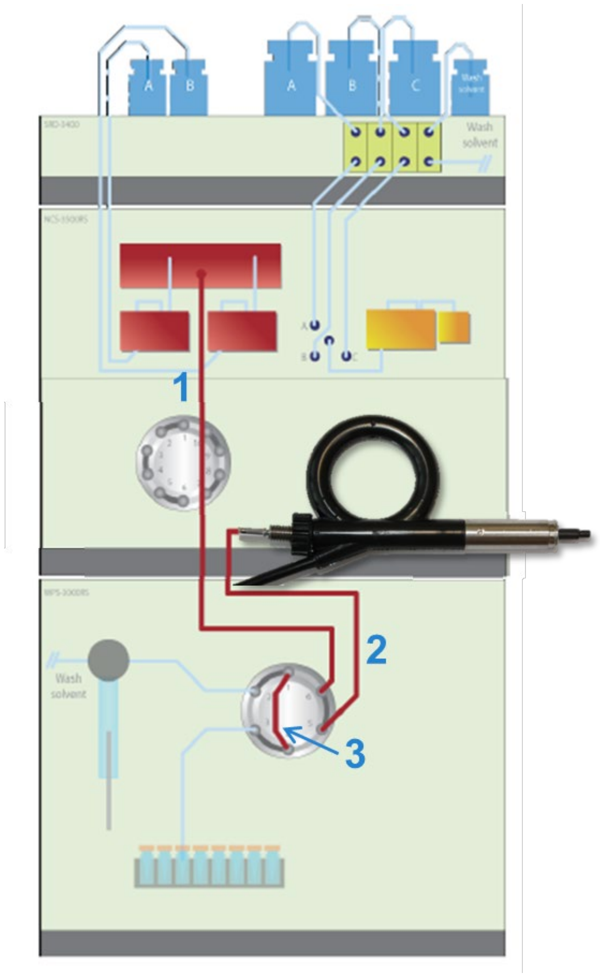

#	Part	PN
1	nanoViper capillary FS/PEEK sheathed 1/32'' I.D. x L 50 µm x 550 mm	6041.5560
2	nanoViper capillary FS/PEEK sheathed 1/32'' I.D. x L 50 µm x 750 mm	6041.5580
3	nanoViper sample loop 20 µL, FS/PEEK sheathed	6826.2420
	EasySpray column PepMap RSLC C_{18} 2 µm, 100A, 150 µm x 15 cm	ES806

2) Instrument control software configuration

- 32bit PC: Foundation 3.1, Xcalibur 3.1, SII 1.2, Exactive 2.8SP1.
- 64 bit PC: Foundation 3.1 SP3 or SPE4, Xcalibur 4 or 4.1, SII 1.3, Exactive 2.8SP1 or Exactive 2.9.

3) Samples and solvents

Description	Source	Product Number / Reference
"Blank sample" - 0.1% FA in Water	Supplementary Note 1: "Standard operating procedure - Sample preparation"	Blank
"QC sample"<br>Human 100%<br>iRT 1/30	Supplementary Note 1: "Standard operating procedure - Sample preparation"	QC – 1µg/µL
Mixed Proteomes "Sample A"<br>E. coli 20%<br>Yeast 15%<br>Human 65%<br>iRT 1/30	Supplementary Note 1: "Standard operating procedure - Sample preparation"	Mix A – 1 µg/µL
Mixed Proteomes "Sample B"<br>E. coli 5%<br>Yeast 30%<br>Human 65%<br>iRT 1/30	Supplementary Note 1: "Standard operating procedure - Sample preparation"	Mix B – 1 µg/µL
0.1% FA in Water, OPTIMA LC/MS<br>- LC pump - solvent A	Fisher Chemicals	LS118-500
0.1% FA in 80 % Acetonitrile, OPTIMA LC/MS<br>- LC pump - solvent B<br>- Wash solvent	Fisher Chemicals	LS122-500
Pierce™ LTQ Velos ESI Positive Ion Calibration Solution	Thermo Fisher Scientific	88323
*Microvials PP, 0.3ml with short thread	VWR	548-0440
*Screw cap PP blue 9mm	VWR	548-0088

*Recommended tubes and LC vials (and caps). Other low binding tubes and vials can be used as well.

4) LC and MS preparation and maintenance

Before launching the series of analyses, the LC-MS platform must be prepared through appropriate maintenance operations.

A) Liquid chromatography system

1. Prepare new solvents and subject them to ultrasonic bath for 15 min to remove dissolved gases.
2. Purge “Bothblocks” of NC pump for 30 min.
3. Purge “Flowmeter” of NC pump for 30 min.
4. Perform “Pressure Transducer Test” to verify that the offset of pressure transducers is correct and calibrate them if necessary.
5. Perform “Viscosity measurement” to verify the pump works correctly. The viscosity measured for channel A should be around 100% (+/- 5%). The viscosity measured for channel B should be around 60% (+/- 5%). Apply new viscosity values.
6. Perform syringe priming of autosampler using 10 cycles. Perform needle and fluidics washing with 50 µL of wash solvent.

B) Mass spectrometer

1. For testing if mass spectrometer is operating properly, infuse fresh calibration solution (product number 88323) into H-ESI source using syringe pump. Refer to manual “Q Exactive HF QuickStart Guide” for instructions about parameter settings to be used in Q Exactive HF Tune software for tuning and calibration (section “Getting Ions from Infusion Experiments”). Spray stability must be ≤ 10% (TIC Variation) to allow proper test and calibration (next steps).
2. Run “Isolation Transmission Endurance Test” (in “Extra Evaluation”). Cleaning of quadrupole is required for transmission score below 0.8 (while 1.0 is the optimal value).
3. Calibrate “Trapping Gas Control”. Delta pressure of the instrument should be below 5 bars.
4. Perform “Mass Calibration (pos)”.
5. Run all “Positive Ion Evaluation” procedure. Calibrate all parameters that did not pass the evaluation.

5) LC method

- Solvent A: 0.1 % FA in Water.
- Solvent B: 0.1 % FA in 80 % Acetonitrile.
- Wash solvent: 0.1 % FA in 80 % Acetonitrile.
- Temperature (EASY- SPRAY source): 50°C.
- Injection mode: µLPickup. Transport vial(s) prepared by adding 300 µL of 0.1 % FA in water in Microvials (volume is sufficient for 18 analyses). Alternatively, transport vial(s) can be prepared by adding 5 mL of 0.1 % FA in water in 10 mL vials (PN 6820.0023).Transport vial must be changed (microvial) or clean/renewed (10 mL vials) every day or after a maximum of 18 injections. Position(s) in method have to match physical location.

- Gradient

Time [min]	Flow [µL/min]	% B	Curve
- 13.000	3.000	2.0	5
5.000	3.000	2.0	5
9.000	1.200	8.0	5
58.000	1.200	32.0	5
59.000	3.000	60.0	5

60.000	3.000	98.0	5

- Commands added manually

Time [min]	"Command"
- 13.000	"Sampler.InjectValveToInject"
6.000	"Sampler. InjectValveToLoad"
8.100	"Sampler.Wash"
60.000	"Sampler.InjectValveToInject"

- Complete LC method and screenshots are included in "9) Appendix – 1"

6) MS parameters

- Tune Parameters

Parameter	Value
Spray voltage [kV]	2.00 (adjust +/-0.2 according to spray stability)
Capillary temperature [°C]	250
S-Lens RF level	50

- MS Method

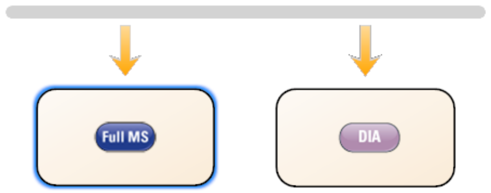

**Parameter**	**Value**
Global settings	
use lock masses	off
Lock mass injection	-
Chrom. peak width (FWHM)	15 s
Time	
Method duration	60 min
Customized Tolerances (+/-)	

Inclusion	-
Lock Masses	-
Exclusion	-
Neutral lost	-
Mass Tag	-
Dynamic Exclusion	-
Full MS	
Runtime	0 to 60 min
Polarity	Positive
In-source CID	0.0 ev
Microscans	1
Resolution	120,000
AGC target	3e6
Maximum IT	50 ms
Number of scan ranges	1
Scan range	400 – 1210
Spectrum type	Profile
DIA	
Runtime	0 to 60 min
Polarity	Positive
In-source CID	0.0 ev
Default charge state	3
Microscan	1
Resolution	30,000
AGC target	1e6
Maximum IT	Auto
Loop count	18

MSX count	1
Isolation window	15 m/z
Isolation offset	0.0 m/z
Fixed first mass	200 m/z
NCE/ stepped NCE	nce : 28
Spectrum data type	Profile

- Full inclusion list for DIA scans is included in “10) Appendix – 2”

7) Samples analyses plan

- Samples analysis plan introduced in Supplementary Note 1 is reminded below.
- The .raw data files must be named under following rule “Lab_W-Day_X-Sample_Y-Rep_Z”, where “W” is the lab number (*e.g.*, “1” or “2”), “X” is the day number (1-7), “Y” is the sample name (“Blank”, “QC”, “A”, or “B”), and “Z” is the replicate number (1-3).
- Some examples of .raw data file names are included below:
    - “Lab_1-Day_1-Sample_Blank-Rep_2”
    - “Lab_1-Day_5-Sample_QC-Rep_1”
    - “Lab_2-Day_3-Sample_A-Rep_3”

Replicates	Day 1	Day 2	Day 3	Day 4	Day 5	Day 6	Day 7
Blank	1	1	1	1	1	1	1
QC sample	3	1	3	1	3	1	3
Blank	1	1	1	1	1	1	1
Sample A	3	1	3	1	3	1	3
Blank	1	1	1	1	1	1	1
Sample B	3	1	3	1	3	1	3
Blank	1	1	1	1	1	1	1
QC sample	1	1	1	1	1	1	1
Blank (for 24 h total instrument time)	4 (up to)	10 (up to)	4 (up to)	10 (up to)	4 (up to)	10 (up to)	4 (up to)

8) General consideration for sample analyses and related data evaluation

- The data processing and evaluation procedure is described in details in Supplementary Note 4.
- In day 1, following initial blank analysis and before triplicated analyses of QC, it is recommended to carry-out a first analysis of QC sample in order to passivate brand new chromatography column. This initial analysis of QC sample must not be included in QC evaluation procedure.
- Each day, the evaluation of "blank" analysis must be performed before starting analyses of "QC sample". In case the evaluation criteria are not fulfilled, stop LC-MS/MS analyses sequence and troubleshoot possible issues (refer to troubleshooting guides of instruments). Repeat failed analysis once the issue has been fixed.
- In days 1, 3, 5, and 7, the evaluation of "QC sample" analyses in triplicate must be performed before starting analyses of "Sample A". In case the QC criteria are not fulfilled, stop LC-MS/MS analyses sequence and troubleshoot possible issues (refer to troubleshooting guide of instruments). Repeat failed analyses once the issue has been fixed.
- In general, the evaluation of data should be carried out in the course of the study to detect major instrument dysfunction as soon as possible and stop LC-MS/MS analyses sequence in order to avoid wasting samples.

9) Appendix – 1: Complete LC method and screenshots

Complete LC method script :

- {Initial Time} Instrument Setup
- PumpModule.LoadingPump.%A.Equate "%A 0.1% FA"
- PumpModule.LoadingPump.%B.Equate "%B 0.1% FA ACN"
- PumpModule.LoadingPump.%C.Equate "%C"
- PumpModule.LoadingPump.Pressure.LowerLimit 0 [bar]
- PumpModule.LoadingPump.Pressure.UpperLimit 500 [bar]
- PumpModule.LoadingPump.MaximumFlowRampUp 998 [µl/min²]
- PumpModule.LoadingPump.MaximumFlowRampDown 998 [µl/min²]
- PumpModule.NC_Pump.%A.Equate "%A 0.1% FA"
- PumpModule.NC_Pump.%B.Equate "%B 0.1% FA in 80% ACN"
- PumpModule.NC_Pump.Pressure.LowerLimit 0 [bar]
- PumpModule.NC_Pump.Pressure.UpperLimit 900 [bar]
- PumpModule.NC_Pump.MaximumFlowRampUp 99.000 [µl/min²]
- PumpModule.NC_Pump.MaximumFlowRampDown 99.000 [µl/min²]
- ColumnOven.TempCtrl Off
- Sampler.LowDispersionMode Off
- Sampler.WashSpeed 4.000 [µl/s]
- Sampler.WashVolume 50.000 [µl]
- Sampler.PunctureDepth 6.000 [mm]
- Sampler.SampleHeight 1.000 [mm]
- Sampler.WasteSpeed 4.000 [µl/s]
- Sampler.DispenseDelay 2.000 [s]
- Sampler.DispSpeed 2.000 [µl/s]
- Sampler.DrawSpeed 0.200 [µl/s]
- Sampler.DrawDelay 5.000 [s]
- Sampler.RinseBetweenReinjections Yes
- Sampler.FlushVolume 6.000 [µl]
- Sampler.TransVialPunctureDepth 6.000 [mm]
- Sampler.TransLiquidHeight 3.000 [mm]
- Sampler.TransportVialCapacity 99999
- Sampler.LastTransportVial RA8
- Sampler.FirstTransportVial RA8
- Sampler.InjectMode ulPickUp
- Sampler.LoopWashFactor 2.000
- Sampler.PumpDevice "NC_Pump"
- Sampler.TempCtrl On
- Sampler.Temperature.Nominal 5.0 [°C]
- Sampler.ReadyTempDelta None
- Sampler.Temperature.LowerLimit 4.0 [°C]
- Sampler.Temperature.UpperLimit 45.0 [°C]
- -13.000 Equilibration Duration = 13.000 [min]
- Sampler.InjectValveToInject
- PumpModule.LoadingPump.Flow.Nominal 0.000 [µl/min]
- PumpModule.LoadingPump.%B.Value 0.0 [%]
- PumpModule.LoadingPump.%C.Value 0.0 [%]
- PumpModule.LoadingPump.Curve 5
- PumpModule.NC_Pump.Flow.Nominal 3.000 [µl/min]
- PumpModule.NC_Pump.%B.Value 2.0 [%]
- PumpModule.NC_Pump.Curve 5
- 0.000 Inject Preparation
- Wait PumpModule.LoadingPump.Ready And PumpModule.NC_Pump.Ready And ColumnOven.Ready And Sampler.Ready

- 0.000 Inject
- Sampler.Inject
- 0.000 Start Run
- ColumnOven.ColumnOven_Temp.AcqOn
- PumpModule.LoadingPump.LoadingPump_Pressure.AcqOn
- PumpModule.NC_Pump.NC_Pump_Pressure.AcqOn
- 0.000 Run Duration = 60.000 [min]
- PumpModule.LoadingPump.Flow.Nominal 0.000 [µl/min]
- PumpModule.LoadingPump.%B.Value 0.0 [%]
- PumpModule.LoadingPump.%C.Value 0.0 [%]
- PumpModule.LoadingPump.Curve 5
- 5.000
- PumpModule.NC_Pump.Flow.Nominal 3.000 [µl/min]
- PumpModule.NC_Pump.%B.Value 2.0 [%]
- PumpModule.NC_Pump.Curve 5
- 6.000
- Sampler.InjectValveToLoad

- 8.100
- Sampler.Wash
- 9.000
- PumpModule.NC_Pump.Flow.Nominal 1.200 [µl/min]
- PumpModule.NC_Pump.%B.Value 8.0 [%]
- PumpModule.NC_Pump.Curve 5
- 58.000
- PumpModule.NC_Pump.Flow.Nominal 1.200 [µl/min]
- PumpModule.NC_Pump.%B.Value 32.0 [%]
- PumpModule.NC_Pump.Curve 5
- 59.000
- PumpModule.NC_Pump.Flow.Nominal 3.000 [µl/min]
- PumpModule.NC_Pump.%B.Value 60.0 [%]
- PumpModule.NC_Pump.Curve 5
- 60.000
- Sampler.InjectValveToInject
- PumpModule.NC_Pump.Flow.Nominal 3.000 [µl/min]
- PumpModule.NC_Pump.%B.Value 98.0 [%]
- PumpModule.NC_Pump.Curve 5
- 60.000 Stop Run
- ColumnOven.ColumnOven_Temp.AcqOff
- PumpModule.LoadingPump.LoadingPump_Pressure.AcqOff
- PumpModule.NC_Pump.NC_Pump_Pressure.AcqOff
- End

Complete LC method screenshots :

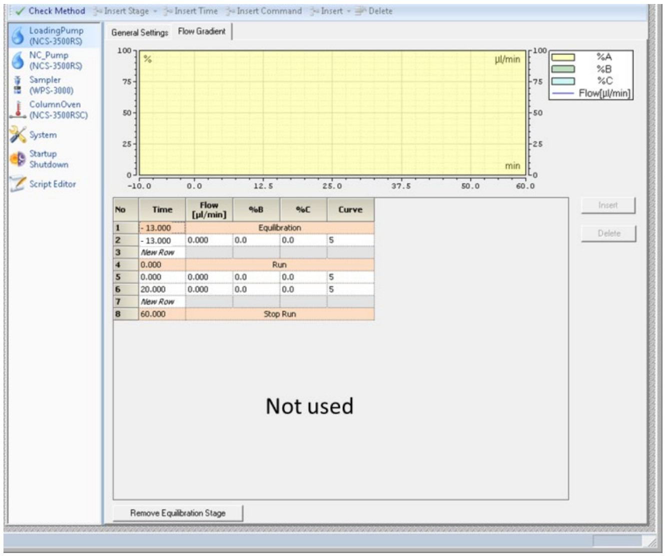

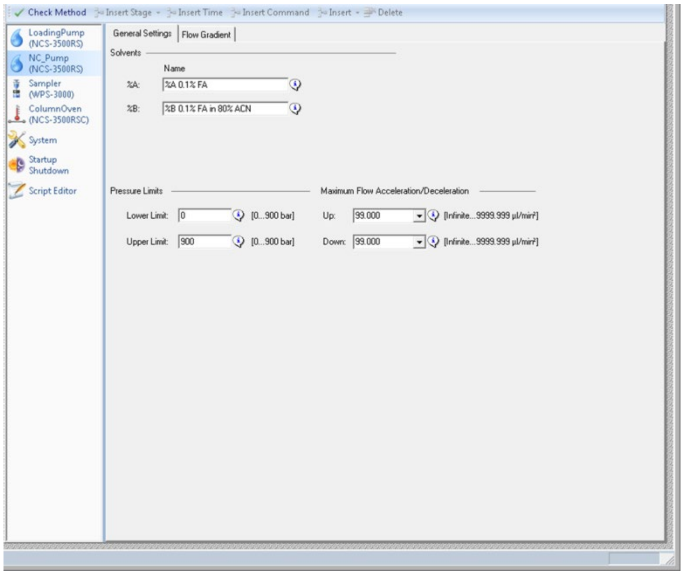
Check Method
Insert Stage
Insert Time
Insert Command
Insert
Delete
LoadingPump (NCS-3500RS)
NC_Pump (NCS-3500RS)
Sampler (WPS-3000)
ColumnOven (NCS-3500RSC)
System
Startup Shutdown
Script Editor
General Settings
Flow Gradient
Solvents
Name
%A: %A 0.1% FA
%B: %B 0.1% FA in 80% ACN
Pressure Limits
Lower Limit: 0 [0...900 bar]
Upper Limit: 900 [0...900 bar]
Maximum Flow Acceleration/Deceleration
Up: 99.000 [Infinite...9999.999 µl/min²]
Down: 99.000 [Infinite...9999.999 µl/min²]

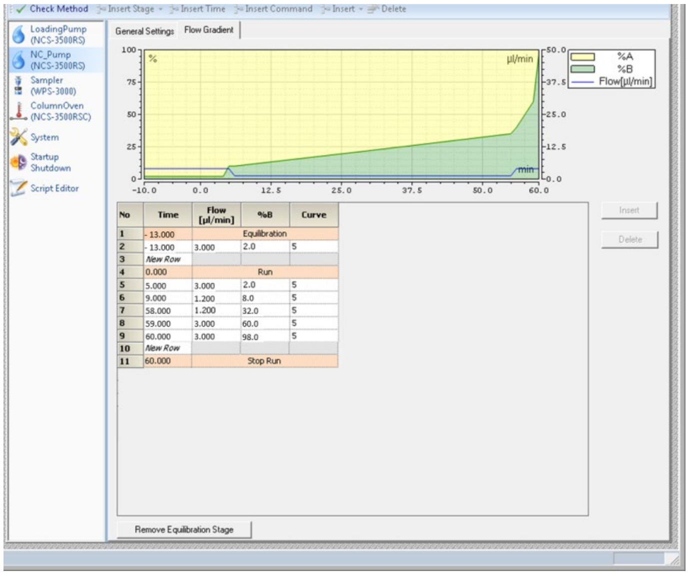
Check Method
Insert Stage
Insert Time
Insert Command
Insert
Delete
LoadingPump (NCS-3500RS)
NC_Pump (NCS-3500RS)
Sampler (WPS-3000)
ColumnOven (NCS-3500RSC)
System
Startup Shutdown
Script Editor
General Settings
Flow Gradient
%
µl/min
min
%A
%B
Flow[µl/min]
No | Time | Flow [µl/min] | %B | Curve
1 | -13.000 | Equilibration
2 | -13.000 | 3.000 | 2.0 | 5
3 | New Row
4 | 0.000 | Run
5 | 5.000 | 3.000 | 2.0 | 5
6 | 9.000 | 1.200 | 8.0 | 5
7 | 58.000 | 1.200 | 32.0 | 5
8 | 59.000 | 3.000 | 60.0 | 5
9 | 60.000 | 3.000 | 98.0 | 5
10 | New Row
11 | 60.000 | Stop Run
Insert
Delete
Remove Equilibration Stage

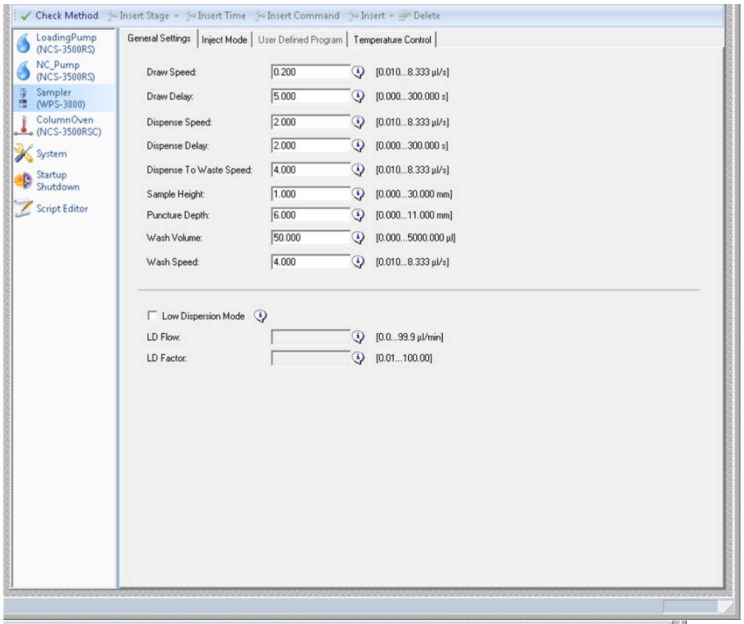

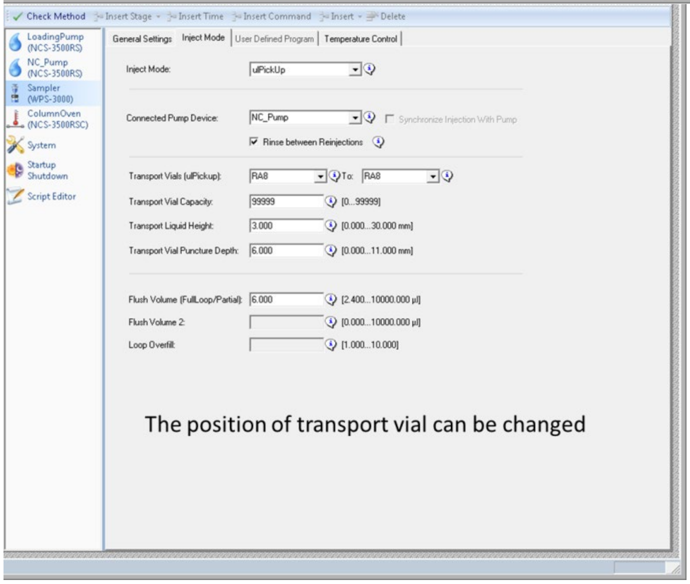

The position of transport vial can be changed

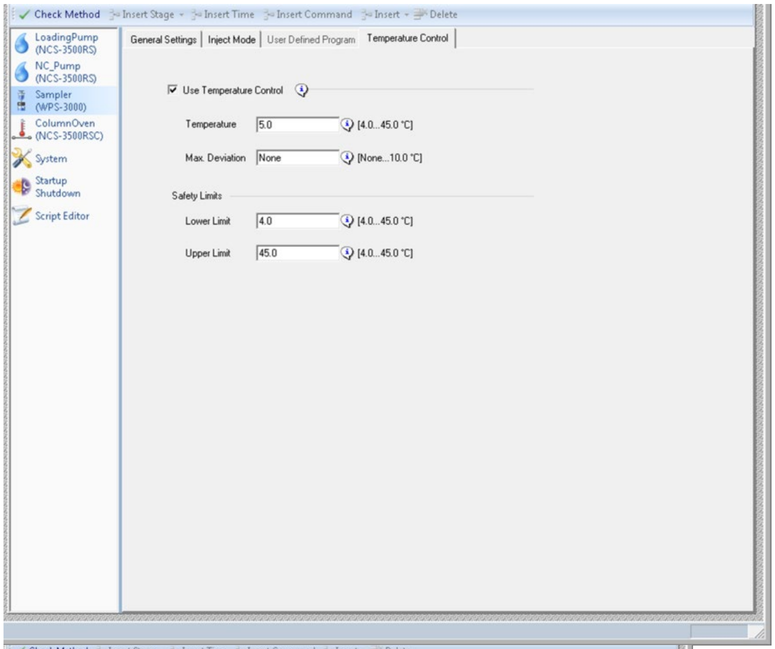
Check Method
Insert Stage
Insert Time
Insert Command
Insert
Delete
LoadingPump (NCS-3500RS)
NC_Pump (NCS-3500RS)
Sampler (WPS-3000)
ColumnOven (NCS-3500RSC)
System
Startup Shutdown
Script Editor
General Settings
Inject Mode
User Defined Program
Temperature Control
Use Temperature Control
Temperature 5.0 [4.0...45.0 °C]
Max. Deviation None [None...10.0 °C]
Safety Limits
Lower Limit 4.0 [4.0...45.0 °C]
Upper Limit 45.0 [4.0...45.0 °C]

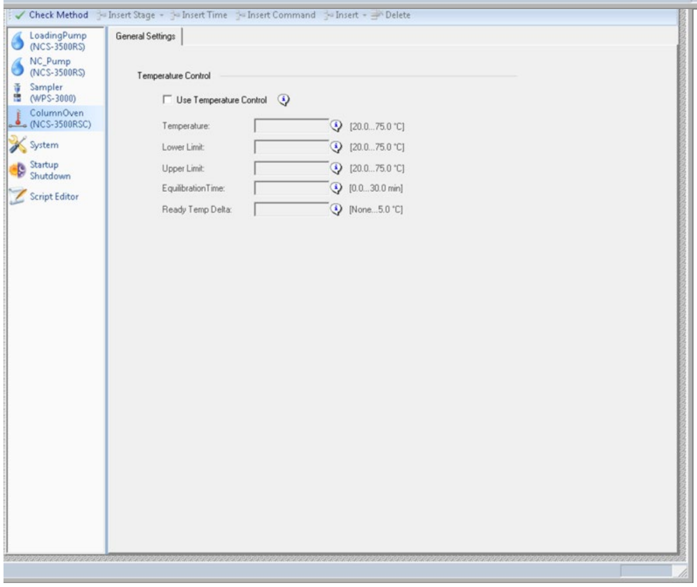
Check Method
Insert Stage
Insert Time
Insert Command
Insert
Delete
LoadingPump (NCS-3500RS)
NC_Pump (NCS-3500RS)
Sampler (WPS-3000)
ColumnOven (NCS-3500RSC)
System
Startup Shutdown
Script Editor
General Settings
Temperature Control
Use Temperature Control
Temperature: [20.0...75.0 °C]
Lower Limit: [20.0...75.0 °C]
Upper Limit: [20.0...75.0 °C]
EquilibrationTime: [0.0...30.0 min]
Ready Temp Delta: [None...5.0 °C]

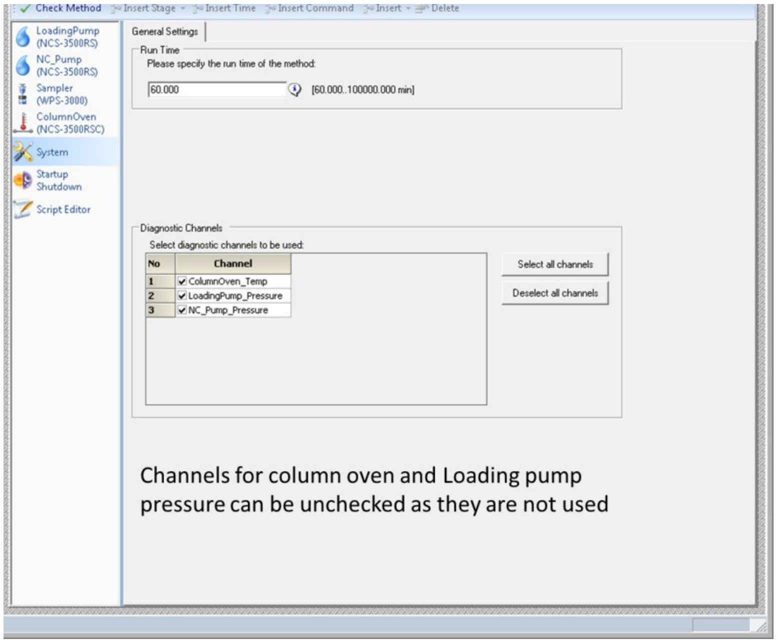
Check Method
Insert Stage
Insert Time
Insert Command
Insert
Delete
LoadingPump (NCS-3500RS)
NC_Pump (NCS-3500RS)
Sampler (WPS-3000)
ColumnOven (NCS-3500RSC)
System
Startup Shutdown
Script Editor
General Settings
Run Time
Please specify the run time of the method:
60.000
[60.000..100000.000 min]
Diagnostic Channels
Select diagnostic channels to be used:
No
Channel
1 ColumnOven_Temp
2 LoadingPump_Pressure
3 NC_Pump_Pressure
Select all channels
Deselect all channels
Channels for column oven and Loading pump pressure can be unchecked as they are not used

10) Appendix – 2: Full inclusion list for DIA scans

Mass [m/z]	Formula [M]	Formula type	Species	CS [z]	Polarity	Start [min]	End [min]	(N) CE	(N)CE type	MSX ID	Comment
407.5					Positive						
422.5					Positive						
437.5					Positive						
452.5					Positive						
467.5					Positive						
482.5					Positive						
497.5					Positive						
512.5					Positive						
527.5					Positive						
542.5					Positive						
557.5					Positive						
572.5					Positive						
587.5					Positive						
602.5					Positive						
617.5					Positive						
632.5					Positive						
647.5					Positive						
662.5					Positive						
677.5					Positive						
692.5					Positive						
707.5					Positive						
722.5					Positive						
737.5					Positive						

752.5					Positi ve						
767.5					Positi ve						
782.5					Positi ve						
797.5					Positi ve						
812.5					Positi ve						
827.5					Positi ve						
842.5					Positi ve						
857.5					Positi ve						
872.5					Positi ve						
887.5					Positi ve						
902.5					Positi ve						
917.5					Positi ve						
932.5					Positi ve						
947.5					Positi ve						
962.5					Positi ve						
977.5					Positi ve						
992.5					Positi ve						
1007.5					Positi ve						
1022.5					Positi ve						
1037.5					Positi ve						
1052.5					Positi ve						
1067.5					Positi ve						
1082.5					Positi ve						
1097.5					Positi ve						
1112.5					Positi ve						

1127.5					Positi ve						
1142.5					Positi ve						
1157.5					Positi ve						
1172.5					Positi ve						
1187.5					Positi ve						
1202.5					Positi ve						

Supplementary Note 3: Standard operating procedure - DIA Analysis with Capillary-flow Easy nLC 1200

1) Hardware configuration

- Mass Spectrometer: Q Exactive HF.

- Chromatographic System: Easy nLC 1200 operated in a one-column setup:

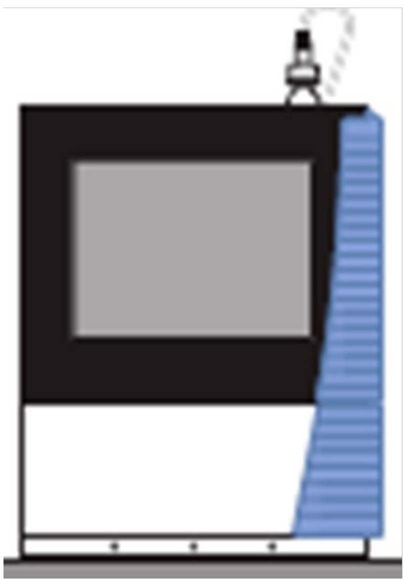

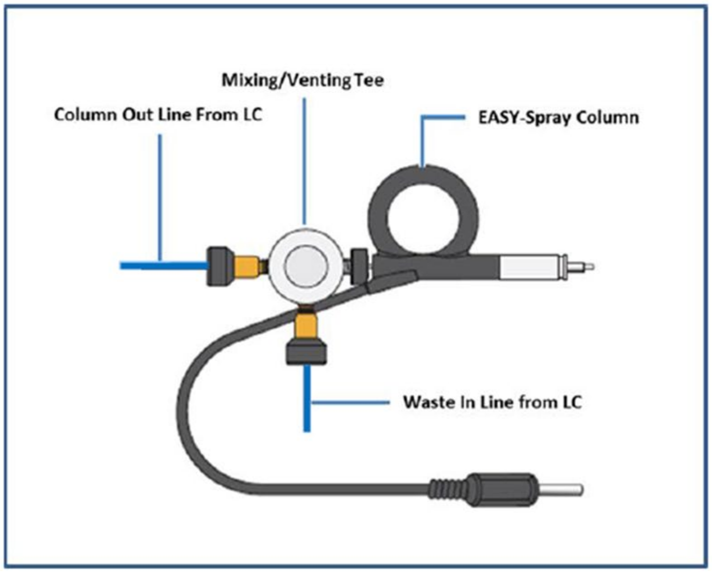

Part	PN
nanoViper sample loop 20 µL, FS/PEEK sheathed	6826.2420
EASY-Spray column PepMap RSLC C_{18} 2 µm, 100A, 150 µm x 15 cm	ES806
Column out line from LC: nanoViper, 20 µm ID, 550 mm length	6041.5261 (LC560)

2) Instrument control software configuration

- 32bit PC: Foundation 3.1, Xcalibur 3.1, Exactive 2.8SP1, EASY-nLC 1200 system: LC Devices 3.00.
- 64 bit PC: Foundation 3.1 SP3 or SPE4, Xcalibur 4 or 4.1, Exactive 2.8SP1 or Exactive 2.9, EASY-nLC 1200 system: LC Devices 3.00.

3) Samples and solvents

**Description**	**Source**	**Product Number / Reference**
"Blank sample" - 0.1% FA in Water	Supplementary Note 1: "Standard operating procedure - Sample preparation"	Blank
"QC sample"<br>Human 100%<br>iRT 1/30	Supplementary Note 1: "Standard operating procedure - Sample preparation	QC – 1µg/µL
Mixed Proteomes "Sample A"<br>E. coli 20%<br>Yeast 15%<br>Human 65%<br>iRT 1/30	Supplementary Note 1: "Standard operating procedure - Sample preparation	Mix A – 1 µg/µL
Mixed Proteomes "Sample B"<br>E. coli 5%<br>Yeast 30%<br>Human 65%<br>iRT 1/30	Supplementary Note 1: "Standard operating procedure - Sample preparation	Mix B – 1 µg/µL
0.1% FA in Water, OPTIMA LC/MS<br>- LC pump - solvent A<br>- Wash solvent – bottle 3	Fisher Chemicals	LS118-500
0.1% FA in 80 % Acetonitrile, OPTIMA LC/MS<br>- LC pump - solvent B<br>- Wash solvent – bottle 1	Fisher Chemicals	LS122-500
Pierce™ LTQ Velos ESI Positive Ion Calibration Solution	Thermo Fisher Scientific	88323
*Microvials PP, 0.3ml with short thread	VWR	548-0440
*Screw cap PP blue 9mm	VWR	548-0088

*Recommended tubes and LC vials (and caps). Other low binding tubes and vials are fine as well.

4) LC and MS preparation and maintenance

Before launching the series of analyses, the LC-MS platform must be prepared through appropriate maintenance operations.

C) Liquid chromatography system

1. Prepare new solvents and subject them to ultrasonic bath for 15 min to remove dissolved gases.
2. Purge solvent of pump A, pump B, and pump S using 5 iterations.
3. Flush air of pump A, pump B, and pump S until the required threshold volume of 10 µL is reached.
4. Calibrate flow sensors for A and B solvents if different solvent composition was used previously.

D) Mass spectrometer

1. For testing if mass spectrometer is operating properly, infuse fresh calibration solution (product number 88323) into H-ESI source using syringe pump. Refer to manual "Q Exactive HF QuickStart Guide" for instructions about parameter settings to be used in Q Exactive HF Tune software for tuning and calibration (section "Getting Ions from Infusion Experiments"). Spray stability must be ≤ 10% (TIC Variation) to allow proper test and calibration (next steps).
2. Run "Isolation Transmission Endurance Test" (in "Extra Evaluation"). Cleaning of quadrupole is required for transmission score below 0.8 (while 1.0 is the optimal value).
3. Calibrate "Trapping Gas Control". Delta pressure of the instrument should be below 5 bars.
4. Perform "Mass Calibration (pos)".
5. Run all "Positive Ion Evaluation" procedure. Calibrate all parameters that did not pass the evaluation.

5) LC method

- Solvent A: 0.1 % FA in Water.
- Solvent B: 0.1 % FA in 80 % Acetonitrile.
- Wash solvent: 0.1 % FA in Water (bottle 3); 0.1 % FA in 80 % Acetonitrile (bottle 1).
- Temperature (EASY- Spray source): 50°C.
- Temperature autosampler: 5°C.

.

- Gradient

Time [min]	Duration[min]	Flow rate [µL/min]	%B
0.000	N/A	1 200	2.0
4.000	4.000	1 200	8.0
53.000	49.000	1 200	32.0
54.000	1.000	1 200	60.0
55.000	1.000	2 000	98.0
65.000	10.000	2 000	98.0

- Additional settings

	Volume [µL]	Flow [µL/min]	Max. pressure [Bar]
Sample pickup	2	10.00	-

Sample loading	20	4.00	1000.00
Analytical column equilibration	20	3.00	1000.00

- Screenshots of the complete method are included in “9) Appendix – 1”

6) MS parameters

- Tune Parameters

Parameter	Value
Spray voltage [kV]	2.00 (adjust +/-0.2 according to spray stability)
Capillary temperature [°C]	250
S-Lens RF level	50

- MS Method

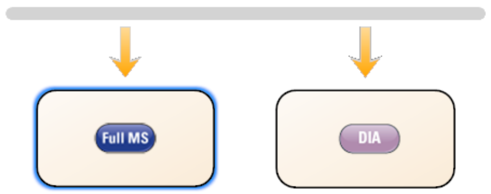

**Parameter**	**Value**
Global settings	
use lock masses	off
Lock mass injection	-
Chrom. peak width (FWHM)	15 s
Time	
Method duration	60 min
Customized Tolerances (+/-)	
Inclusion	-
Lock Masses	-
Exclusion	-
Neutral lost	-

Mass Tag	-
Dynamic Exclusion	-
Full MS	
Runtime	0 to 60 min
Polarity	Positive
In-source CID	0.0 ev
Microscans	1
Resolution	120,000
AGC target	3e6
Maximum IT	50 ms
Number of scan ranges	1
Scan range	400 – 1210
Spectrum type	Profile
DIA	
Runtime	0 to 60 min
Polarity	Positive
In-source CID	0.0 ev
Default charge state	3
Microscan	1
Resolution	30,000
AGC target	1e6
Maximum IT	Auto
Loop count	18
MSX count	1
Isolation window	15 m/z
Isolation offset	0.0 m/z
Fixed first mass	200 m/z

NCE/ stepped NCE	nce : 28
Spectrum data type	Profile

- Full inclusion list for DIA scans is included in “10) Appendix – 2”

7) Samples analyses plan

- Samples analysis plan introduced in Supplementary Note 1 is reminded below.
- The .raw data files must be named under following rule "Lab_W-Day_X-Sample_Y-Rep_Z", where "W" is the lab number (*e.g.*, "1" or "2"), "X" is the day number (1-7), "Y" is the sample name ("Blank", "QC", "A", or "B"), and "Z" is the replicate number (1-3).
- Some examples of .raw data file names are included below:
    - "Lab_1-Day_1-Sample_Blank-Rep_2"
    - "Lab_1-Day_5-Sample_QC-Rep_1"
    - "Lab_2-Day_3-Sample_A-Rep_3"

Replicates	Day 1	Day 2	Day 3	Day 4	Day 5	Day 6	Day 7
Blank	1	1	1	1	1	1	1
QC sample	3	1	3	1	3	1	3
Blank	1	1	1	1	1	1	1
Sample A	3	1	3	1	3	1	3
Blank	1	1	1	1	1	1	1
Sample B	3	1	3	1	3	1	3
Blank	1	1	1	1	1	1	1
QC sample	1	1	1	1	1	1	1
Blank (for 24 h total instrument time)	4 (up to)	10 (up to)	4 (up to)	10 (up to)	4 (up to)	10 (up to)	4 (up to)

8) General consideration for sample analyses and related data evaluation

- The data processing and evaluation procedure is described in details in Supplementary Note 4.
- In day 1, following initial blank analysis and before triplicated analyses of QC, it is recommended to carry-out a first analysis of QC sample in order to passivate brand new chromatography column. This initial analysis of QC sample must not be included in QC evaluation procedure.
- Each day, the evaluation of "blank" analysis must be performed before starting analyses of "QC sample". In case the evaluation criteria are not fulfilled, stop LC-MS/MS analyses sequence and troubleshoot possible issues (refer to troubleshooting guides of instruments). Repeat failed analysis once the issue has been fixed.
- In days 1, 3, 5, and 7, the evaluation of "QC sample" analyses in triplicate must be performed before starting analyses of "Sample A". In case the QC criteria are not fulfilled, stop LC-MS/MS analyses sequence

and troubleshoot possible issues (refer to troubleshooting guide of instruments). Repeat failed analyses once the issue has been fixed.

- In general, the evaluation of data should be carried out in the course of the study to detect major instrument dysfunction as soon as possible and stop LC-MS/MS analyses sequence in order to avoid wasting samples.

9) Appendix – 1: Complete LC method screenshots

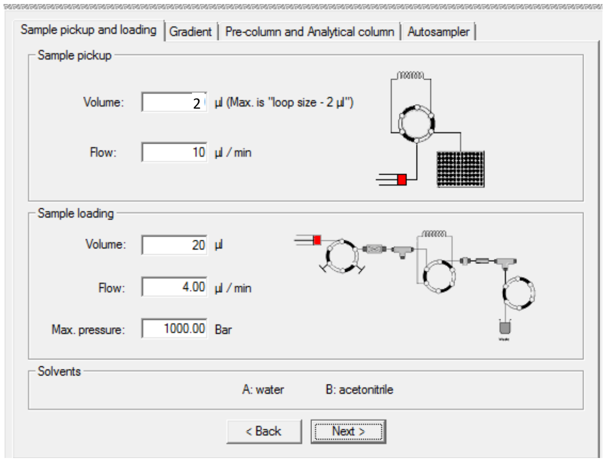

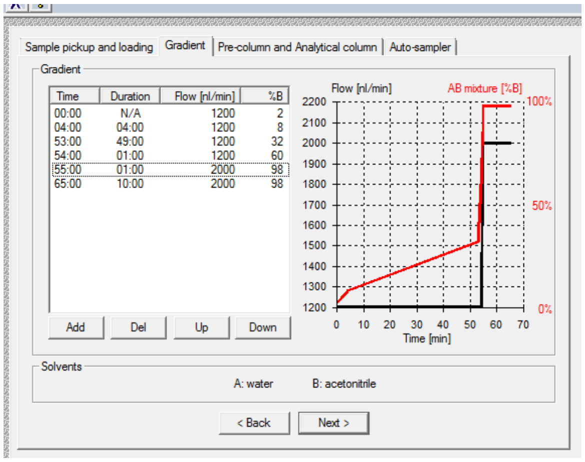

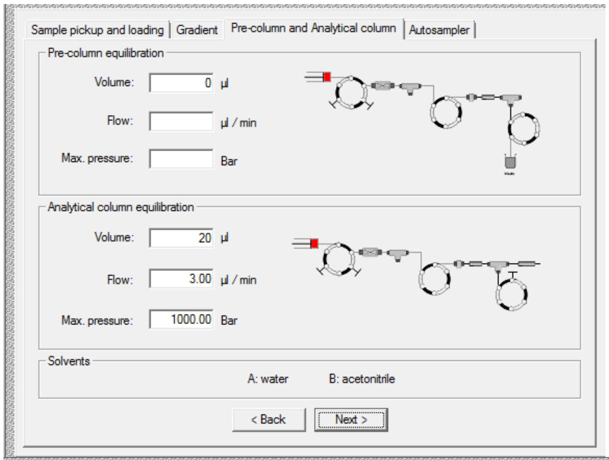
Sample pickup and loading
Gradient
Pre-column and Analytical column
Autosampler
Pre-column equilibration
Volume:
0
µl
Flow:
µl / min
Max. pressure:
Bar
Analytical column equilibration
Volume:
20
µl
Flow:
3.00
µl / min
Max. pressure:
1000.00
Bar
Solvents
A: water
B: acetonitrile
< Back
Next >

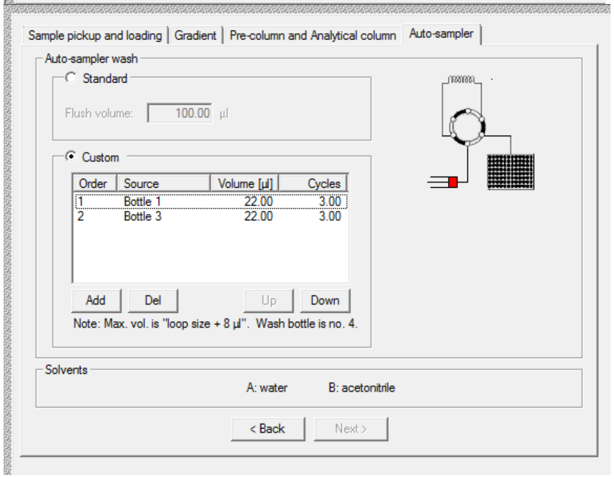
Sample pickup and loading
Gradient
Pre-column and Analytical column
Auto-sampler
Auto-sampler wash
Standard
Flush volume:
100.00
µl
Custom
Order
Source
Volume [µl]
Cycles
1
Bottle 1
22.00
3.00
2
Bottle 3
22.00
3.00
Add
Del
Up
Down
Note: Max. vol. is "loop size + 8 µl". Wash bottle is no. 4.
Solvents
A: water
B: acetonitrile
< Back
Next >

10) Appendix – 2: Full inclusion list for DIA scans

Mass [m/z]	Formula [M]	Formula type	Species	CS [z]	Polarity	Start [min]	End [min]	(N) CE	(N)CE type	MSX ID	Comment
407.5					Positive						
422.5					Positive						
437.5					Positive						
452.5					Positive						
467.5					Positive						
482.5					Positive						
497.5					Positive						
512.5					Positive						
527.5					Positive						
542.5					Positive						
557.5					Positive						
572.5					Positive						
587.5					Positive						
602.5					Positive						
617.5					Positive						
632.5					Positive						
647.5					Positive						
662.5					Positive						
677.5					Positive						
692.5					Positive						
707.5					Positive						
722.5					Positive						
737.5					Positive						

752.5					Positi ve						
767.5					Positi ve						
782.5					Positi ve						
797.5					Positi ve						
812.5					Positi ve						
827.5					Positi ve						
842.5					Positi ve						
857.5					Positi ve						
872.5					Positi ve						
887.5					Positi ve						
902.5					Positi ve						
917.5					Positi ve						
932.5					Positi ve						
947.5					Positi ve						
962.5					Positi ve						
977.5					Positi ve						
992.5					Positi ve						
1007.5					Positi ve						
1022.5					Positi ve						
1037.5					Positi ve						
1052.5					Positi ve						
1067.5					Positi ve						
1082.5					Positi ve						
1097.5					Positi ve						
1112.5					Positi ve						

1127.5					Positive						
1142.5					Positive						
1157.5					Positive						
1172.5					Positive						
1187.5					Positive						
1202.5					Positive						

Supplementary Note 4: Standard operating procedure - DIA Analyses and Data Evaluation

I) General Considerations

- The samples analyses plan is provided in Section 3 of Supplementary Note 1.
- Sections V to VI in the document below describe the data processing / evaluation procedure for "Day 1" experiment. The procedure must be repeated for "Day 3", "Day 5, and "Day 7".
- Section VII describes the centralized data processing of controlled samples A and B analyses performed across the different lab and days retained.

II) Data processing software

- Biognosys Spectronaut Pulsar, version 11 and higher.
- Xcalibur, version 3.1 or higher.

III) Preliminary operations

1. Copy on processing computer spectral library files "ES806_KG_36F.kit" (Human), "yeast_ES806_12F.kit" (Yeast), and Ecoli_ES806_12F.kit (E. coli).
2. Copy on processing computer protein databases "swissprot_homo_201604.fasta" (Human), "Saccharomyces cerevisiae_201602" (Yeast), and "Uniprot-Ecoli_K12_201709.fasta" (E. coli).
3. Copy on processing computer report templates for proteins "quanBenchmark_protein.rs" and peptides "quanBenchmark_peptides.rs".

IV) Preparation in Spectronaut

1. Start the program "Spectronaut".
2. Select the tab: Prepare.
3. Press the link: Import Spectral Library.
4. In popup window, select Human spectral library file.
5. Press the button: Open.
6. Repeat steps IV – 3, 4, and 5 for Yeast spectral library, and then E. coli spectral library.
7. Select the tab: Databases.
8. Select the subtab: Protein Databases.
9. Press the link: Import.
10. In popup window, select Human protein database.
11. In popup window, select Parsing Rule: Uniprot FASTA.
12. In popup window, press the link: Import.
13. Repeat steps IV – 9, 10, 11, and 12 for Yeast protein database, and then E. coli protein database.
14. Select the tab: Report.
15. Press the link: Import Schema.
16. In popup window, select protein report template.
17. Press the button: Open.
18. Repeat steps IV – 15, 16, and 17 for peptide report template.

V) Data processing of QC sample analyses with Spectronaut

1. Start the program "Spectronaut".
2. Select the tab: Review.
3. Press the link: Load Raw from File
    a. In popup window, select the three QC analyses replicate.
    b. In popup window, press the button: Open
4. In window "Experiment Setup":
    a. Enter as Experiment name "Lab_W-Day_X-Sample_QC" (where "W" is the lab number [*e.g.*, "1" or "2"] and "X" is the day number [1, 3, 5, or 7]).
    b. Select the Tab: FASTA Files
    c. Click in checkbox of Human Protein Database
    d. Press the Link: Configure Conditions
    e. In window "Condition Editors", Enter "Sample_QC" in 3 cells of column "Condition" (each sample QC raw file row). Press the link: Apply
    f. Press the Link: Spectral Library"
    g. In window "Import Spectral Library", select tab "From Prepare Perspective". Select Human Spectral Library. Press link: Load.
    h. Select the Tab: Analysis Settings.
    i. Select Schema "BGS Factory Settings (default") if not already selected.
    j. Verify that all parameters of the different subsections of "BGS Factory Settings" are set at default values (refer to screenshot below, section VIII). Check more specifically that in subsection "Quantification", parameter "Quantify MS-Level" is set at "MS1".
    k. Press the link: Start.
5. At the end of data processing, righ-click in the left panel (tab including experiment name) and select "Save as". In popup window, enter "Lab_W-Day_X-Sample_QC-SpectronautAnalysis" (where "W" is the lab number [*e.g.*, "1" or "2] and "X" is the day number [1, 3, 5, or 7]) and press button: Save.
6. Select Tab: Report
7. In left panel "Schemas", select QuanBenchmark_proteins
8. Press the link: Export Report. In popup window, enter "Lab_W-Day_X-Sample_QC-ReportProteins" (where "W" is the lab number [*e.g.*, "1" or "2"] and "X" is the day number [1, 3, 5, or 7]) and press button: Save.
9. In left panel "Schemas", select QuanBenchmark_peptides
10. Press the link: Export Report. In popup window, enter "Lab_W-Day_X-Sample_QC-ReportPeptides" (where "W" is the lab number [*e.g.*, "1" or "2"] and "X" is the day number [1, 3, 5, or 7]) and press button: Save.

VI) On-site evaluation of QC sample analyses with Spectronaut

1. In Spectronaut, open Spectronaut file "Lab_W-Day_X-Sample_QC-SpectronautAnalysis" if not opened yet.
    a. Select the tab: Review
    b. Press the link: Load Spectronaut Experiment.
    c. In popup window, select file "Lab_W-Day_X-Sample_QC-SpectronautAnalysis"
    d. In popup window, press the button: Open.
2. In Spectronaut, select the tab: Review.
    a. In main window, select "Analysis Summary" in drop-down menu "Lower panel".

   b. Extract values indicated in lower panel for “Median Peak Width”, “Data Points per Peak (MS1)”, and “Data points per Peak (MS2).
3. In Spectronaut, select the tab: Post Analysis.
   a. In left panel, in subsection “Analysis Overview”, select “Coefficients of Variation”. Precursor CV distribution graph is displayed, including median value. By right clicking in the graph and selecting “CV Base” -> “Peptide” or “Protein Group”, Peptide or Protein Group CV graph can be displayed.
   b. Extract the different median values.
4. In Spectronaut, in the tab: Post Analysis / left panel / subsection “Analysis Overview”, select “CVs below X”.
   a. Graph “Precursor CVs below X” is displayed. By right clicking in the graph and selecting “Show Point Values”, direct access to numerical values are provided by moving the cursor over the different columns of the histogram (numbers of precursors “identified” / “identified with CV<20%” / “identified with CV<10%”). By right clicking in the graph and selecting “CV Base” -> “Peptide” or “Protein Group”, graphs “Peptide CVs below X” or “Protein Group CVs below X” can be displayed.
   b. Extract the numbers of precursors/peptides/proteins “identified”, “identified with CV<20%”, and “identified with CV<10%”.

VII) Centralized data processing of Samples A and B with Spectronaut

The following procedure was only applied at one lab were all the data were centralized.

1. Start the program “Spectronaut”.
2. Select the tab: Review.
3. Press the link: Load Raw from File
   a. In popup window, select the three Sample A analyses and three Sample B analyses for each lab and day retained.
   b. In popup window, press the button: Open
4. In window “Experiment Setup”:
   a. Enter as Experiment name “MultiSite-Sample_A-B”.
   b. Select the Tab: FASTA Files
   c. Click in checkbox of Human Protein Database, Yeast Protein Database, and E. coli Protein Database.
   d. Press the Link: Configure Conditions
   e. In window “Condition Editors”, Enter “Lab_W-Day_X-Sample_Y” (where “W” is the lab number [*e.g.*, “1” or “2”], “X” is the day number [1, 3, 5, or 7], and “Y” is the sample name [A or B]) in the pertinent cells of column “Condition”. Press the link: Apply.
   f. Press the Link: Spectral Library”
   g. In window “Import Spectral Library”, select tab “From Prepare Perspective”. Select Human Spectral Library. Press link: Load.
   h. Repeat step VII 4 – g for Yeast Spectral library, and then E. coli Spectral Library.
   i. Select the Tab: Analysis Settings.
   j. Select Schema “BGS Factory Settings (default”) if not already selected.
   k. Verify that all parameters of the different subsections of “BGS Factory Settings” are set at default values (refer to screenshot below, section VIII). Check more specifically that in subsection “Quantification”, parameter “Quantify MS-Level” is set at “MS1”.
   l. Press the link: Start.

5. At the end of data processing, righ-click in the left panel (tab including experiment name) and select “Save as”. In popup window, enter “MultiSite-Sample_A-B” and press button: Save.
6. Select Tab: Report
7. In left panel “Schemas”, select QuanBenchmark_proteins
8. Press the link: Export Report. In popup window, enter “ID_Controlled-Samples_MultiSite_ReportProteins” and press button: Save.
9. In left panel “Schemas”, select QuanBenchmark_peptides
10. Press the link: Export Report. In popup window, “ID_Controlled-Samples_MultiSite_ReportPeptides” and press button: Save.

VIII) Screenshots required values for parameters in Tab “Analysis Settings” / Schema “BGS Factory Settings” / Subsection “Quantifications” (unless specified differently)

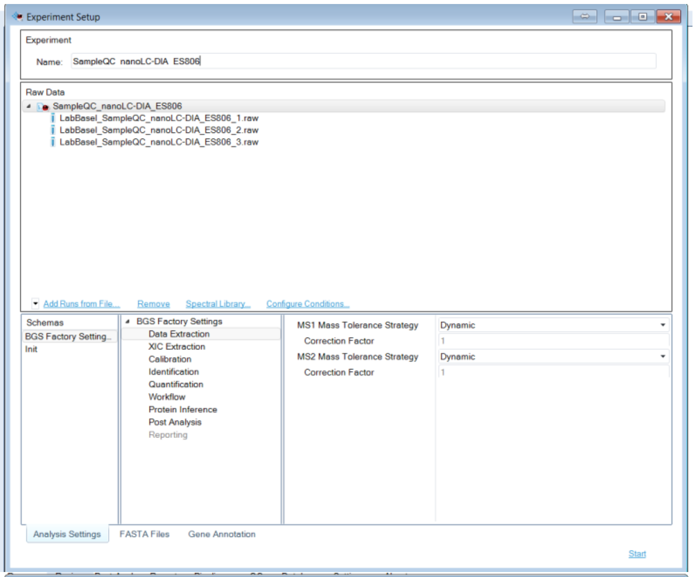

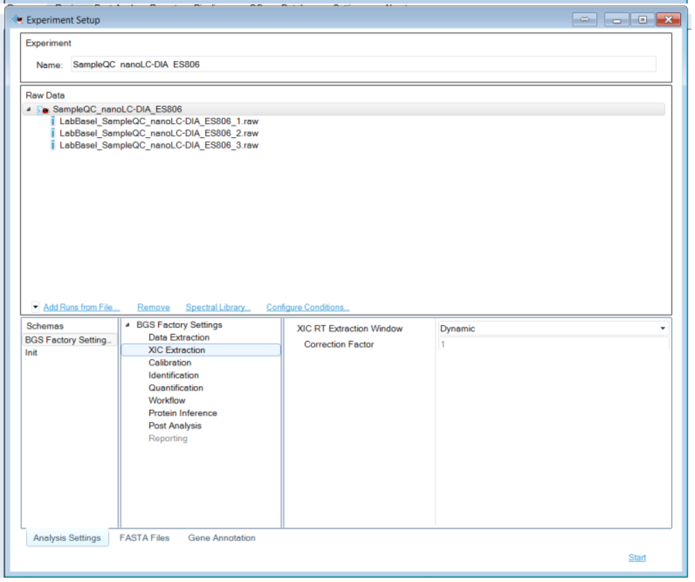

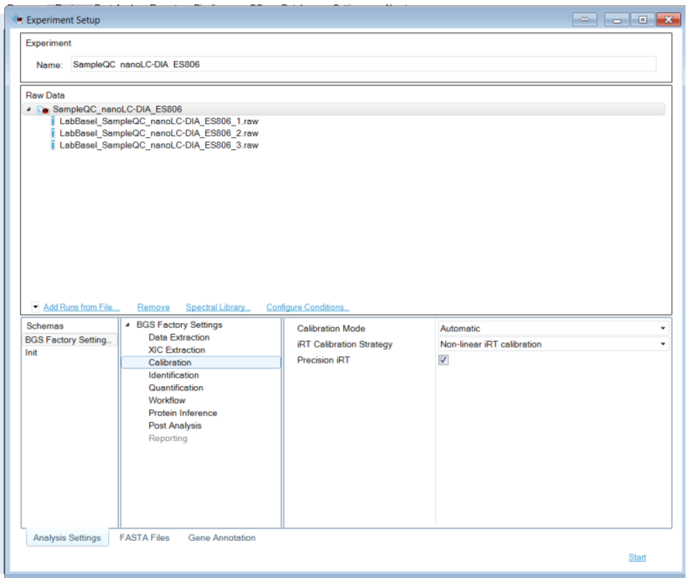
Experiment Setup
Experiment
Name: SampleQC nanoLC-DIA ES806
Raw Data
SampleQC_nanoLC-DIA_ES806
LabBasel_SampleQC_nanoLC-DIA_ES806_1.raw
LabBasel_SampleQC_nanoLC-DIA_ES806_2.raw
LabBasel_SampleQC_nanoLC-DIA_ES806_3.raw
Add Runs from File...
Remove
Spectral Library...
Configure Conditions...
Schemas
BGS Factory Setting...
Init
BGS Factory Settings
Data Extraction
XIC Extraction
Calibration
Identification
Quantification
Workflow
Protein Inference
Post Analysis
Reporting
Calibration Mode
Automatic
iRT Calibration Strategy
Non-linear iRT calibration
Precision iRT
Analysis Settings
FASTA Files
Gene Annotation
Start

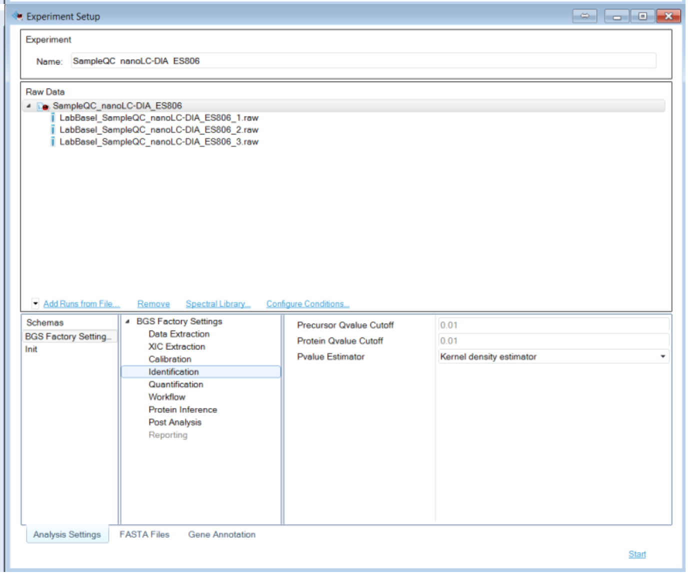
Experiment Setup
Experiment
Name: SampleQC nanoLC-DIA ES806
Raw Data
SampleQC_nanoLC-DIA_ES806
LabBasel_SampleQC_nanoLC-DIA_ES806_1.raw
LabBasel_SampleQC_nanoLC-DIA_ES806_2.raw
LabBasel_SampleQC_nanoLC-DIA_ES806_3.raw
Add Runs from File...
Remove
Spectral Library...
Configure Conditions...
Schemas
BGS Factory Setting...
Init
BGS Factory Settings
Data Extraction
XIC Extraction
Calibration
Identification
Quantification
Workflow
Protein Inference
Post Analysis
Reporting
Precursor Qvalue Cutoff
0.01
Protein Qvalue Cutoff
0.01
Pvalue Estimator
Kernel density estimator
Analysis Settings
FASTA Files
Gene Annotation
Start

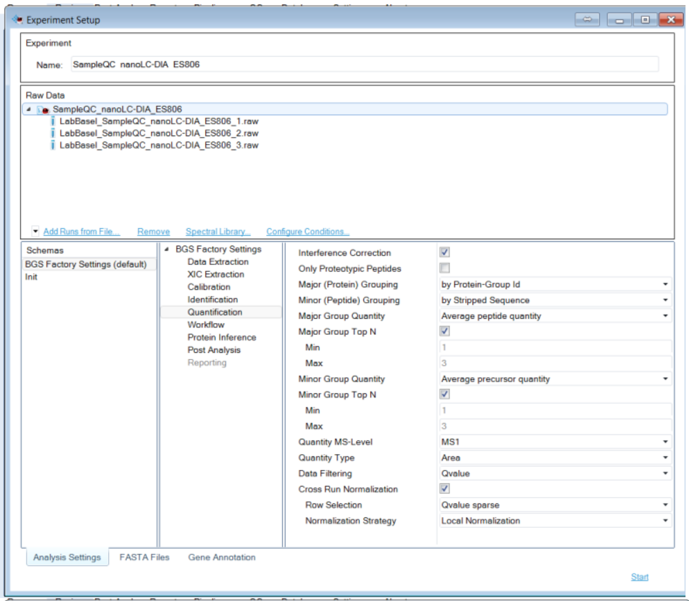
Experiment Setup
Experiment
Name: SampleQC nanoLC-DIA ES806
Raw Data
SampleQC_nanoLC-DIA_ES806
LabBasel_SampleQC_nanoLC-DIA_ES806_1.raw
LabBasel_SampleQC_nanoLC-DIA_ES806_2.raw
LabBasel_SampleQC_nanoLC-DIA_ES806_3.raw
Add Runs from File... Remove Spectral Library... Configure Conditions...
Schemas
BGS Factory Settings (default)
Init
BGS Factory Settings
Data Extraction
XIC Extraction
Calibration
Identification
Quantification
Workflow
Protein Inference
Post Analysis
Reporting
Interference Correction
Only Proteotypic Peptides
Major (Protein) Grouping: by Protein-Group Id
Minor (Peptide) Grouping: by Stripped Sequence
Major Group Quantity: Average peptide quantity
Major Group Top N
Min 1
Max 3
Minor Group Quantity: Average precursor quantity
Minor Group Top N
Min 1
Max 3
Quantity MS-Level: MS1
Quantity Type: Area
Data Filtering: Qvalue
Cross Run Normalization
Row Selection: Qvalue sparse
Normalization Strategy: Local Normalization
Analysis Settings FASTA Files Gene Annotation
Start

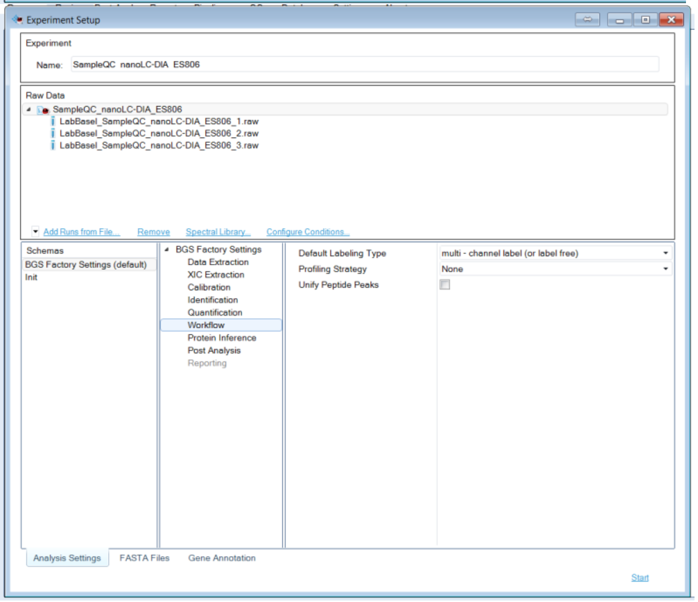
Experiment Setup
Experiment
Name: SampleQC nanoLC-DIA ES806
Raw Data
SampleQC_nanoLC-DIA_ES806
LabBasel_SampleQC_nanoLC-DIA_ES806_1.raw
LabBasel_SampleQC_nanoLC-DIA_ES806_2.raw
LabBasel_SampleQC_nanoLC-DIA_ES806_3.raw
Add Runs from File... Remove Spectral Library... Configure Conditions...
Schemas
BGS Factory Settings (default)
Init
BGS Factory Settings
Data Extraction
XIC Extraction
Calibration
Identification
Quantification
Workflow
Protein Inference
Post Analysis
Reporting
Default Labeling Type: multi - channel label (or label free)
Profiling Strategy: None
Unify Peptide Peaks
Analysis Settings FASTA Files Gene Annotation
Start

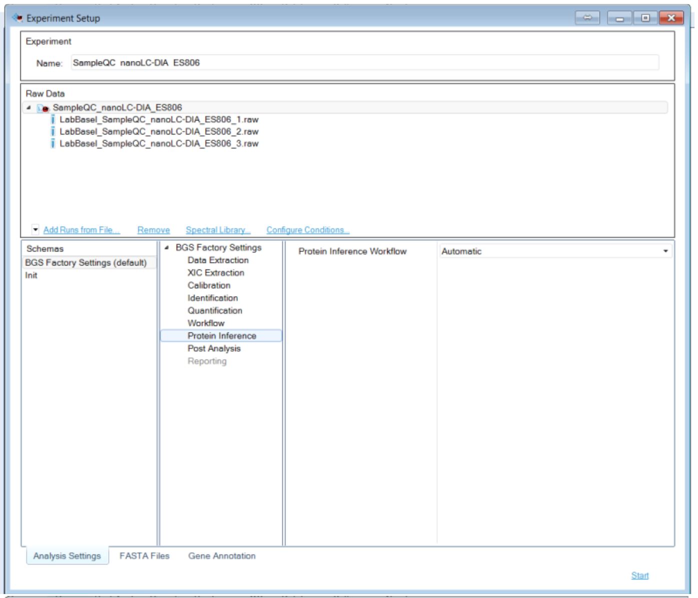
Experiment Setup
Experiment
Name: SampleQC nanoLC-DIA ES806
Raw Data
SampleQC_nanoLC-DIA_ES806
LabBasel_SampleQC_nanoLC-DIA_ES806_1.raw
LabBasel_SampleQC_nanoLC-DIA_ES806_2.raw
LabBasel_SampleQC_nanoLC-DIA_ES806_3.raw
Add Runs from File...
Remove
Spectral Library...
Configure Conditions...
Schemas
BGS Factory Settings (default)
Init
BGS Factory Settings
Data Extraction
XIC Extraction
Calibration
Identification
Quantification
Workflow
Protein Inference
Post Analysis
Reporting
Protein Inference Workflow
Automatic
Analysis Settings
FASTA Files
Gene Annotation
Start

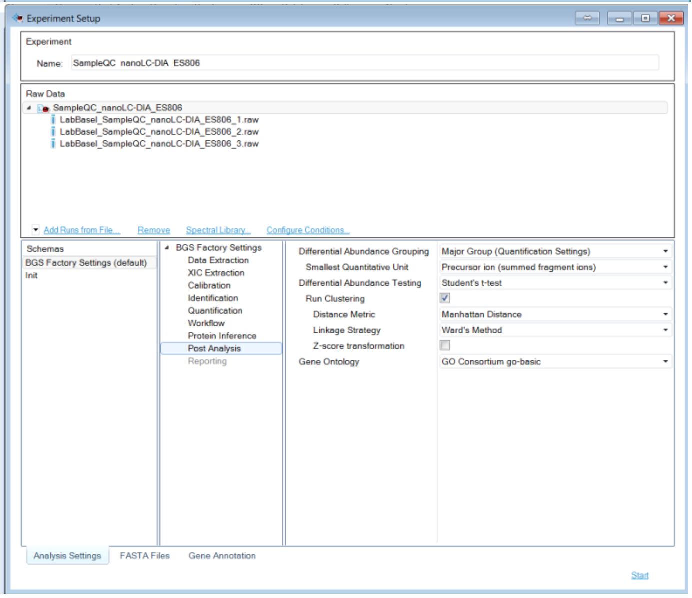
Experiment Setup
Experiment
Name: SampleQC nanoLC-DIA ES806
Raw Data
SampleQC_nanoLC-DIA_ES806
LabBasel_SampleQC_nanoLC-DIA_ES806_1.raw
LabBasel_SampleQC_nanoLC-DIA_ES806_2.raw
LabBasel_SampleQC_nanoLC-DIA_ES806_3.raw
Add Runs from File...
Remove
Spectral Library...
Configure Conditions...
Schemas
BGS Factory Settings (default)
Init
BGS Factory Settings
Data Extraction
XIC Extraction
Calibration
Identification
Quantification
Workflow
Protein Inference
Post Analysis
Reporting
Differential Abundance Grouping
Major Group (Quantification Settings)
Smallest Quantitative Unit
Precursor ion (summed fragment ions)
Differential Abundance Testing
Student's t-test
Run Clustering
Distance Metric
Manhattan Distance
Linkage Strategy
Ward's Method
Z-score transformation
Gene Ontology
GO Consortium go-basic
Analysis Settings
FASTA Files
Gene Annotation
Start
