# Supplementary material for: Standardization and harmonization of distributed multi-center proteotype analysis supporting precision medicine studies
Source: Nat Commun. 2020 Oct 16;11:5248. doi: 10.1038/s41467-020-18904-9 (PMC7568553; doi:10.1038/s41467-020-18904-9)
Supplement: Supplementary file 3 — Description of Additional Supplementary Files [file 41467_2020_18904_MOESM3_ESM.docx]

**Description of Supplementary Files**

**Supplementary Data 1:** QC-DIA benchmark proteins quantified by analytical site.

**Supplementary Data** **2:** MultiSite proteins quantification results of controlled samples (Log2 ratio protein abundance (Sample A / Sample B).

**Supplementary Data 3:** Tumor tissue proteins quantified by analytical site.

**Supplementary Data 4:** Significantly altered/ histotype correlated protein alterations.

**Supplementary Data 5:** The relative standard deviation (RSD) of 18 HGSOC and OCCC signature proteins across three analytical sites for each patient tissue sample.

**Supplementary Software:** R package / script for peptides to proteins rollup and quantification
