# Supplementary material for: Standardization and harmonization of distributed multi-center proteotype analysis supporting precision medicine studies
Source: Nat Commun. 2020 Oct 16;11:5248. doi: 10.1038/s41467-020-18904-9 (PMC7568553; doi:10.1038/s41467-020-18904-9)
Supplement: Supplementary file 9 — Supplementary Software [file 41467_2020_18904_MOESM9_ESM.zip › moonshot/html/00Index.html]

R: Support package for moonshot project

# Support package for moonshot project

---

## Documentation for package ‘moonshot’ version 0.1.3

- DESCRIPTION file.

## Help Pages

|  |  |
| --- | --- |
| buildProteinSpeciesDictionary | buildProteinSpeciesDictionary |
| calFDR | calFDR |
| calPerformanceCurves | calPerformanceCurves\_ |
| calPerformanceCurves\_ | calPerformanceCurves\_ |
| consolidateIntensities | consolidateIntensities |
| filterFromCommonProteinList | filterFromCommonProteinList |
| get\_sample\_vector | get\_sample\_vector given a sample names vector v, it classifies names by samples (grouping technical replicates together) |
| medianLogPeptidesMedianRepl | calculate the median of the log ratios of peptides, then calculate the median of technical replicates for each study variable |
| proteinCount | proteinCount |
| readSpectronautFiles | Read Spectronaut Files |
| readSpectronautFiles\_ | Read Spectronaut Files |
| rollupAndFDR | Roll-up and FDR |
| setConfig | setConfig |
| speciesScatterPlot | speciesScatterPlot |
