# Supplementary material for: Standardization and harmonization of distributed multi-center proteotype analysis supporting precision medicine studies
Source: Nat Commun. 2020 Oct 16;11:5248. doi: 10.1038/s41467-020-18904-9 (PMC7568553; doi:10.1038/s41467-020-18904-9)
Supplement: Supplementary file 9 — Supplementary Software [file 41467_2020_18904_MOESM9_ESM.zip › moonshot/html/buildProteinSpeciesDictionary.html]

R: buildProteinSpeciesDictionary

|  |  |
| --- | --- |
| buildProteinSpeciesDictionary {moonshot} | R Documentation |

## buildProteinSpeciesDictionary

### Description

builds a unique protein - species dictionary for all data sets.

### Usage

```
buildProteinSpeciesDictionary(peptideDatasets)
```

### Arguments

|  |  |
| --- | --- |
| `pepDs` | list of data.frames (output from moonshot::readSpectronautFiles() |

### Value

dictionary of key:protein, value:species

---

[Package *moonshot* version 0.1.3 Index]
