# Supplementary material for: Standardization and harmonization of distributed multi-center proteotype analysis supporting precision medicine studies
Source: Nat Commun. 2020 Oct 16;11:5248. doi: 10.1038/s41467-020-18904-9 (PMC7568553; doi:10.1038/s41467-020-18904-9)
Supplement: Supplementary file 9 — Supplementary Software [file 41467_2020_18904_MOESM9_ESM.zip › moonshot/html/calFDR.html]

R: calFDR

|  |  |
| --- | --- |
| calFDR {moonshot} | R Documentation |

## calFDR

### Description

Calculate p-value FDR by using a background method

### Usage

```
calFDR(values, w, slicing = NULL, showPlot = F, saveImage = F,
  addSufix = "", eps = NA, sampleBackground = NA,
  distanceMethod = "euclidean", clusterMethod = "DBSCAN",
  pointsDiscardedForBackground = NULL)
```

### Arguments

|  |  |
| --- | --- |
| `values` | vector with values to estimate p-values and FDR |
| `w` | vector with weights for the vector values |
| `slicing` | number of slicing points (neighbors) in order to calculate the p-value |
| `showPlot` | show a plot of the calculated data |
| `saveImage` | save image of the plot into a file (filename provided in this parameter). This makes showPlot = TRUE |
| `addSufix` | adds a sufix to the image file name |
| `eps` | use eps distance for clustering. If NA provided then eps distance will be automatically estimated. |
| `sampleBackground` | ratio of data used for estimating the background. If NA all points will be used. |
| `distanceMethod` | method for calculating distance among values. Default: euclidean |
| `clusterMethod` | method for clustering. Options: c("DBSCAN", "OPTICS") |
| `pointsDiscardedForBackground` | bool vector of the same size as values indicating which points should be discarded in order to estimate the background |

### Value

data.frame with ids, ranks, p-values, and FDR

### Author(s)

Pedro Navarro

---

[Package *moonshot* version 0.1.3 Index]
