# Supplementary material for: Standardization and harmonization of distributed multi-center proteotype analysis supporting precision medicine studies
Source: Nat Commun. 2020 Oct 16;11:5248. doi: 10.1038/s41467-020-18904-9 (PMC7568553; doi:10.1038/s41467-020-18904-9)
Supplement: Supplementary file 9 — Supplementary Software [file 41467_2020_18904_MOESM9_ESM.zip › moonshot/html/calPerformanceCurves.html]

R: calPerformanceCurves\_

|  |  |
| --- | --- |
| calPerformanceCurves {moonshot} | R Documentation |

## calPerformanceCurves\_

### Description

A wrapper for plotting different performance curves from PD tables

### Usage

```
calPerformanceCurves(condition, ratiosFdrsMethods, ...)
```

### Arguments

|  |  |
| --- | --- |
| `condition` | condition name as expressed in PD |
| `ratiosFDRsMethods` | PD tables (actually other external tables should work as well) containing the data for the analysis. When more than one table is given, performance curves are plotted for each table. |

---

[Package *moonshot* version 0.1.3 Index]
