# Supplementary material for: Standardization and harmonization of distributed multi-center proteotype analysis supporting precision medicine studies
Source: Nat Commun. 2020 Oct 16;11:5248. doi: 10.1038/s41467-020-18904-9 (PMC7568553; doi:10.1038/s41467-020-18904-9)
Supplement: Supplementary file 9 — Supplementary Software [file 41467_2020_18904_MOESM9_ESM.zip › moonshot/html/calPerformanceCurves_.html]

R: calPerformanceCurves\_

|  |  |
| --- | --- |
| calPerformanceCurves\_ {moonshot} | R Documentation |

## calPerformanceCurves\_

### Description

A wrapper for plotting different performance curves from PD tables

### Usage

```
calPerformanceCurves_(condition, ratiosFdrsMethods = c(), classifierTag,
  m_labels, pvalueTags, methodNames = names(ratiosFdrsMethods),
  pngBaseName = NA, figIncreaseFactor = 1, legendFontFactor = 1,
  main = NA, recallLim = c(0, 1), specificityLim = c(0, 1),
  sensitivityLim = c(0, 1), precisionLim = c(0, 1), fprLim = c(0, 1),
  tprLim = c(0, 1),
  colorPalette = (grDevices::colorRampPalette(brewer.pal(9,
  "Set1")[-3]))(length(ratiosFdrsMethods)), perfCurves = "ALL",
  removeLastPoint = FALSE)
```

### Arguments

|  |  |
| --- | --- |
| `condition` | condition name as expressed in PD |
| `classifierTag` | tag to find the column containing the classification |
| `m_labels` | vector containing the classification labels (example: c(FALSE, TRUE)). The second element is considered the True label. |
| `pvalueTags` | tag to find the column of the classification score |
| `methodNames` | names will be displayed at the legend |
| `pngBaseName` | when not NA, plots are stored in png files (using the string pngBaseName as file base name) |
| `figIncreaseFactor` | when exporting to png, cex factor to modify plot width |
| `legendFontFactor` | when exporting to png, cex factor to modify legend font |
| `main` | figure main name. If not specified a generic name for each performance curve plot. |
| `recallLim` | limit for recall. Default: c(0, 1). |
| `specificityLim` | limit for specificity. Default: c(0, 1). |
| `sensitivityLim` | limit for sensitivity. Default: c(0, 1). |
| `precisionLim` | limit for precision. Default: c(0, 1). |
| `fprLim` | limit for FPR. Default: c(0, 1). |
| `tprLim` | limit for TPR. Default: c(0, 1). |
| `colorPalette` | color palette used for curves. |
| `perfCurves` | Performance curves to be displayed. Default: ALL |
| `removeLastPoint` | Remove the last point of the curve. Useful when a set of TN has been introduced with the lowest score. |
| `ratiosFDRsMethods` | PD tables (actually other external tables should work as well) containing the data for the analysis. When more than one table is given, performance curves are plotted for each table. |

---

[Package *moonshot* version 0.1.3 Index]
