# Supplementary material for: Standardization and harmonization of distributed multi-center proteotype analysis supporting precision medicine studies
Source: Nat Commun. 2020 Oct 16;11:5248. doi: 10.1038/s41467-020-18904-9 (PMC7568553; doi:10.1038/s41467-020-18904-9)
Supplement: Supplementary file 9 — Supplementary Software [file 41467_2020_18904_MOESM9_ESM.zip › moonshot/html/consolidateIntensities.html]

R: consolidateIntensities

|  |  |
| --- | --- |
| consolidateIntensities {moonshot} | R Documentation |

## consolidateIntensities

### Description

It fits intensity values according to a set of descriptive ratios between intensities by doing an optimization of minimum squares

### Usage

```
consolidateIntensities(prtable, fnscale = 0.01)
```

### Arguments

|  |  |
| --- | --- |
| `prtable` | A protein table that follows this structure: ProteinID, InitialIntensityA, InitialIntensityB, ..., nPeptides, log2Ratio(B/A), log2Ratio(C/A), ... |
| `fnscale` | function scale for optim |

### Value

data.frame with consolidated intensities

---

[Package *moonshot* version 0.1.3 Index]
