# Supplementary material for: Standardization and harmonization of distributed multi-center proteotype analysis supporting precision medicine studies
Source: Nat Commun. 2020 Oct 16;11:5248. doi: 10.1038/s41467-020-18904-9 (PMC7568553; doi:10.1038/s41467-020-18904-9)
Supplement: Supplementary file 9 — Supplementary Software [file 41467_2020_18904_MOESM9_ESM.zip › moonshot/html/filterFromCommonProteinList.html]

R: filterFromCommonProteinList

|  |  |
| --- | --- |
| filterFromCommonProteinList {moonshot} | R Documentation |

## filterFromCommonProteinList

### Description

filter protein results by using a list of common proteins

### Usage

```
filterFromCommonProteinList(results, proteinSpeciesDict)
```

### Arguments

|  |  |
| --- | --- |
| `results` | protein results (the output from moonshot::rollupAndFDR(peptideDatasets)) |
| `proteinSpeciesDict` | use ms.proteinSpeciesDict |

### Value

a merged data.frame by ProteinID, Species, and Marked

---

[Package *moonshot* version 0.1.3 Index]
