# Supplementary material for: Standardization and harmonization of distributed multi-center proteotype analysis supporting precision medicine studies
Source: Nat Commun. 2020 Oct 16;11:5248. doi: 10.1038/s41467-020-18904-9 (PMC7568553; doi:10.1038/s41467-020-18904-9)
Supplement: Supplementary file 9 — Supplementary Software [file 41467_2020_18904_MOESM9_ESM.zip › moonshot/html/get_sample_vector.html]

R: get\_sample\_vector given a sample names vector v, it...

|  |  |
| --- | --- |
| get\_sample\_vector {moonshot} | R Documentation |

## get\_sample\_vector given a sample names vector v, it classifies names by samples (grouping technical replicates together)

### Description

get\_sample\_vector
given a sample names vector v, it classifies names by samples (grouping technical replicates together)

### Usage

```
get_sample_vector(v, discardFirstColumns = 0)
```

### Arguments

|  |  |
| --- | --- |
| `v` | sample names vector |
| `discardFirstColumns` | skip first column names |

### Value

list of samples

---

[Package *moonshot* version 0.1.3 Index]
