# Supplementary material for: Standardization and harmonization of distributed multi-center proteotype analysis supporting precision medicine studies
Source: Nat Commun. 2020 Oct 16;11:5248. doi: 10.1038/s41467-020-18904-9 (PMC7568553; doi:10.1038/s41467-020-18904-9)
Supplement: Supplementary file 9 — Supplementary Software [file 41467_2020_18904_MOESM9_ESM.zip › moonshot/html/medianLogPeptidesMedianRepl.html]

R: calculate the median of the log ratios of peptides, then...

|  |  |
| --- | --- |
| medianLogPeptidesMedianRepl {moonshot} | R Documentation |

## calculate the median of the log ratios of peptides, then calculate the median of technical replicates for each study variable

### Description

medianLogPeptidesMedianRepl

### Usage

```
medianLogPeptidesMedianRepl(df, minNumberLogRatios = 1, reportVariance = F,
  useDOF = T, verbose = F)
```

### Arguments

|  |  |
| --- | --- |
| `df` | rolled-up protein table with abundances and ratios |
| `minNumberLogRatios` | minimum number of log-ratios required in order to calculate the median (it filters out proteins quantitated by low number of features) |
| `reportVariance` | boolean report variance in the output data.frame |
| `useDOF` | boolean take degrees of freedom into account |
| `verbose` | boolean verbosity of the function messages |

---

[Package *moonshot* version 0.1.3 Index]
