# Supplementary material for: Standardization and harmonization of distributed multi-center proteotype analysis supporting precision medicine studies
Source: Nat Commun. 2020 Oct 16;11:5248. doi: 10.1038/s41467-020-18904-9 (PMC7568553; doi:10.1038/s41467-020-18904-9)
Supplement: Supplementary file 9 — Supplementary Software [file 41467_2020_18904_MOESM9_ESM.zip › moonshot/html/proteinCount.html]

R: proteinCount

|  |  |
| --- | --- |
| proteinCount {moonshot} | R Documentation |

## proteinCount

### Description

count the number of proteins (by species) in all datasets

### Usage

```
proteinCount(results)
```

### Arguments

|  |  |
| --- | --- |
| `list` | of datasets |

### Value

data.frame with protein counts by species

---

[Package *moonshot* version 0.1.3 Index]
