# Supplementary material for: Standardization and harmonization of distributed multi-center proteotype analysis supporting precision medicine studies
Source: Nat Commun. 2020 Oct 16;11:5248. doi: 10.1038/s41467-020-18904-9 (PMC7568553; doi:10.1038/s41467-020-18904-9)
Supplement: Supplementary file 9 — Supplementary Software [file 41467_2020_18904_MOESM9_ESM.zip › moonshot/html/readSpectronautFiles.html]

R: Read Spectronaut Files

|  |  |
| --- | --- |
| readSpectronautFiles {moonshot} | R Documentation |

## Read Spectronaut Files

### Description

Reads Spectronaut files extracting quantitative values for parameter given samples.

### Usage

```
readSpectronautFiles()
```

### Value

list of data frames with digested spectronaut results

### Author(s)

Pedro Navarro

---

[Package *moonshot* version 0.1.3 Index]
