# Supplementary material for: Standardization and harmonization of distributed multi-center proteotype analysis supporting precision medicine studies
Source: Nat Commun. 2020 Oct 16;11:5248. doi: 10.1038/s41467-020-18904-9 (PMC7568553; doi:10.1038/s41467-020-18904-9)
Supplement: Supplementary file 9 — Supplementary Software [file 41467_2020_18904_MOESM9_ESM.zip › moonshot/html/readSpectronautFiles_.html]

R: Read Spectronaut Files

|  |  |
| --- | --- |
| readSpectronautFiles\_ {moonshot} | R Documentation |

## Read Spectronaut Files

### Description

Reads Spectronaut files extracting quantitative values for parameter given samples.

### Usage

```
readSpectronautFiles_(fileName, sampleNames, speciesFasta, precursorIdTag,
  proteinIdTag, quanTag, fastaTag, qValueTag, filteredStr, addLab = T,
  LCMethods = NA, removeInterSpecies = T)
```

### Arguments

|  |  |
| --- | --- |
| `fileName` | full path to Spectronaut file. |
| `sampleNames` | vector with the names of the samples (which will be parsed at the headers) |
| `speciesFasta` | names of the species used in the FASTA file |
| `precursorIdTag` | tag to identify the precursor mass in headers |
| `proteinIdTag` | tag to identify the protein Id in headers |
| `quanTag` | tag to identify quantitative values in headers |
| `fastaTag` | tag to identify the FASTA column (species) in headers |
| `qValueTag` | tag to identify the qValues in headers |
| `addLab` | add Laboratory field (parsed from fileName as "Lab(.\*?)\_") to output data frame |
| `LCMethods` | vector to add liquid chromatography methods to output data frame |
| `removeInterSpecies` | remove inter-species shared peptides |

### Value

data.frame with extracted peptide quantification values from Spectronaut

### Author(s)

Pedro Navarro

---

[Package *moonshot* version 0.1.3 Index]
