# Supplementary material for: Standardization and harmonization of distributed multi-center proteotype analysis supporting precision medicine studies
Source: Nat Commun. 2020 Oct 16;11:5248. doi: 10.1038/s41467-020-18904-9 (PMC7568553; doi:10.1038/s41467-020-18904-9)
Supplement: Supplementary file 9 — Supplementary Software [file 41467_2020_18904_MOESM9_ESM.zip › moonshot/html/rollupAndFDR.html]

R: Roll-up and FDR

|  |  |
| --- | --- |
| rollupAndFDR {moonshot} | R Documentation |

## Roll-up and FDR

### Description

Rolls up peptides to proteins and estimates p-values and FDR for each protein

### Usage

```
rollupAndFDR(peptideDatasets)
```

### Arguments

|  |  |
| --- | --- |
| `peptideDatasets` | list of data.frames (the output from moonshot::readSpectronautFiles()) |

### Value

a list of data frames of proteins with p-values and FDR

---

[Package *moonshot* version 0.1.3 Index]
