# Supplementary material for: Standardization and harmonization of distributed multi-center proteotype analysis supporting precision medicine studies
Source: Nat Commun. 2020 Oct 16;11:5248. doi: 10.1038/s41467-020-18904-9 (PMC7568553; doi:10.1038/s41467-020-18904-9)
Supplement: Supplementary file 9 — Supplementary Software [file 41467_2020_18904_MOESM9_ESM.zip › moonshot/html/setConfig.html]

R: setConfig

|  |  |
| --- | --- |
| setConfig {moonshot} | R Documentation |

## setConfig

### Description

set configuration for moonshot package writing a config object ms.Config in global environment

### Usage

```
setConfig(dataFolder = NA, Laboratories = NA, LCMethods = NA,
  minNumOfPeptides = 1, speciesFasta = c(HUMAN = "homo", YEAST =
  "Saccharomyces", ECOLI = "Ecoli"), speciesReference = "HUMAN",
  sampleNames = c("Sample_A", "Sample_B"),
  peptideQuanTag = "PEP\\.Quantity$", proteinQuanTag = "PG\\.Quantity$",
  proteinIdTag = "PG.ProteinAccessions", precursorIdTag = "EG.PrecursorId",
  fastaColumn = "PG.FASTAName", qValueTag = "Qvalue$",
  filteredStr = "Filtered", classifierTag = "Marked",
  classifierLabels = c(TRUE, FALSE), pvalueTags = "AdjPValue",
  removeInterSpecies = T, minNumberLogRatios = 8,
  numberOfNeighborsForPvalues = 300, fileExtension = ".xls")
```

### Arguments

|  |  |
| --- | --- |
| `dataFolder` | physical path to data folder |
| `Laboratories` | vector containing laboratory names to be analyzed. Data folder must contain folders with these names. |
| `LCMethods` | vector containing liquid chromatography methods to be analyzed. Laboratory folders must contain folders with these names. |
| `minNumOfPeptides` | minimum number of peptides/protein. |
| `speciesFasta` | names of the species used in the FASTA file |
| `speciesReference` | species name to be used as reference (1:1 ratio). In performance curves this species represents the negative results. |
| `sampleNames` | vector with the names of the samples (which will be parsed at the headers) |
| `peptideQuanTag` | tag to identify peptide quantitative values in headers |
| `proteinQuanTag` | tag to identify protein quantitative values in headers |
| `proteinIdTag` | tag to identify protein accessions in headers |
| `precursorIdTag` | tag to identify the precursor mass in headers |
| `fastaColumn` | FASTA column (species) in headers |
| `qValueTag` | tag to identify the qValues in headers |
| `filteredStr` | string used in Spectronaut to indicate that the row has been filtered |
| `classifierTag` | tag for the classifier. |
| `classifierLabels` | Sorted list of classifier levels. Important to sort in the right order! |
| `pvalueTags` | tag to identify p-values in headers |
| `removeInterSpecies` | remove inter-species shared peptides |
| `minNumberLogRatios` | minimum number of log ratios in order to consider a protein correctly quantified in roll-up step. |
| `numberOfNeighborsForPvalues` | number of neighbors considered when calculating p-values (background approach) |
| `fileExtension` | extension used by input files |

### Value

a configuration object "ms.Config" in the global environment

---

[Package *moonshot* version 0.1.3 Index]
