# Supplementary material for: Standardization and harmonization of distributed multi-center proteotype analysis supporting precision medicine studies
Source: Nat Commun. 2020 Oct 16;11:5248. doi: 10.1038/s41467-020-18904-9 (PMC7568553; doi:10.1038/s41467-020-18904-9)
Supplement: Supplementary file 9 — Supplementary Software [file 41467_2020_18904_MOESM9_ESM.zip › moonshot/html/speciesScatterPlot.html]

R: speciesScatterPlot

|  |  |
| --- | --- |
| speciesScatterPlot {moonshot} | R Documentation |

## speciesScatterPlot

### Description

scatter plot of a (protein) dataset separating species by color

### Usage

```
speciesScatterPlot(dataset, ylimit = NULL, noLegend = F)
```

### Arguments

|  |  |
| --- | --- |
| `dataset` | (protein) dataset to be plotted |
| `ylimit` | vector c(y\_min,y\_max) for ylim |
| `noLegend` | boolean display legend? |

### Value

a ggplot2 object

---

[Package *moonshot* version 0.1.3 Index]
