# Supplementary material for: Standardization and harmonization of distributed multi-center proteotype analysis supporting precision medicine studies
Source: Nat Commun. 2020 Oct 16;11:5248. doi: 10.1038/s41467-020-18904-9 (PMC7568553; doi:10.1038/s41467-020-18904-9)
Supplement: Supplementary file 10 — Reporting Summary [file 41467_2020_18904_MOESM10_ESM.pdf]

nature research

Corresponding author(s): Yue Xuan, Bernd Wollscheid, Thomas P. Conrads

Last updated by author(s): Aug 26, 2020

Reporting Summary

Nature Research wishes to improve the reproducibility of the work that we publish. This form provides structure for consistency and transparency in reporting. For further information on Nature Research policies, see Authors & Referees and the Editorial Policy Checklist.

Statistics

For all statistical analyses, confirm that the following items are present in the figure legend, table legend, main text, or Methods section.

n/a	Confirmed	
☐	☒	The exact sample size (*n*) for each experimental group/condition, given as a discrete number and unit of measurement
☐	☒	A statement on whether measurements were taken from distinct samples or whether the same sample was measured repeatedly
☐	☒	The statistical test(s) used AND whether they are one- or two-sided<br>*Only common tests should be described solely by name; describe more complex techniques in the Methods section.*
☐	☒	A description of all covariates tested
☐	☒	A description of any assumptions or corrections, such as tests of normality and adjustment for multiple comparisons
☐	☒	A full description of the statistical parameters including central tendency (e.g. means) or other basic estimates (e.g. regression coefficient) AND variation (e.g. standard deviation) or associated estimates of uncertainty (e.g. confidence intervals)
☐	☒	For null hypothesis testing, the test statistic (e.g. *F*, *t*, *r*) with confidence intervals, effect sizes, degrees of freedom and *P* value noted<br>*Give P values as exact values whenever suitable.*
☒	☐	For Bayesian analysis, information on the choice of priors and Markov chain Monte Carlo settings
☐	☒	For hierarchical and complex designs, identification of the appropriate level for tests and full reporting of outcomes
☐	☒	Estimates of effect sizes (e.g. Cohen's *d*, Pearson's *r*), indicating how they were calculated

Our web collection on statistics for biologists contains articles on many of the points above.

Software and code

Policy information about availability of computer code

Data collection	The analyses of protein digest fractions were performed using an Easy-nLC 1200 (Thermo Fisher Scientific) coupled to Orbitrap Fusion Lumos or Q Exactive HF-X mass spectrometers (Thermo Fisher Scientific) operated with DDA methods. QC samples, controlled samples A and B, and ovarian cancer tissues were analyzed with an Easy-nLC 1200 or an Ultimate 3000 RSLCnano equipped with capillary flow meter coupled to a Q Exactive HF mass spectrometer (ThermoFisher Scientific) operated with DIA methods. Orbitrap Tribrid MS Series Instrument Control Software Version for Lumos is version 3.0. Control software for Ultimate 3000 RSLCnano setup: for a 32bit PC: Foundation 3.1, Xcalibur 3.1, SII 1.2, Exactive 2.8SP1; for a 64 bit PC: Foundation 3.1 SP3 or SPE4, Xcalibur 4 or 4.1, SII 1.3, Exactive 2.8SP1 or Exactive 2.9. Control software for Easy-nLC 1200 setup: for a 32bit PC: Foundation 3.1, Xcalibur 3.1, Exactive 2.8SP1, LC Devices 3.00; for a 64 bit PC: Foundation 3.1 SP3 or SPE4, Xcalibur 4 or 4.1, Exactive 2.8SP1 or Exactive 2.9, LC Devices 3.00.
Data analysis	The assignment of MS/MS spectra generated from DDA analyses of protein digests was made with Proteome Discoverer 2.2 software and Sequest HT (version 1.1.0.178). Proteome discoverer result files were imported into Spectronaut Pulsar 11.0.15038.23.24843 (Asimov) software for the generation of the spectral libraries (.kit files) for each organism using default settings. The processing of DIA analyses was performed using Spectronaut Pulsar 11.0.15038.23.24843 (Asimov) software.<br>Differential analysis of processed histotype-specific HRMS1-DIA data performed using the LIMMA package (version 3.8) in R (version 3.5.2), and cluster analyses was performed using ClustVis (version .0.0.0.900) (https://biit.cs.ut.ee/clustvis/)

For manuscripts utilizing custom algorithms or software that are central to the research but not yet described in published literature, software must be made available to editors/reviewers. We strongly encourage code deposition in a community repository (e.g. GitHub). See the Nature Research guidelines for submitting code & software for further information.

Data

Policy information about availability of data

All manuscripts must include a data availability statement. This statement should provide the following information, where applicable:

- Accession codes, unique identifiers, or web links for publicly available datasets
- A list of figures that have associated raw data
- A description of any restrictions on data availability

The UniProt database released https://www.uniprot.org/downloads for "Homo sapiens" (downloaded April 2016), "Saccharomyces cerevisiae" (downloaded May 2016), or "Escherichia coli" (downloaded February 2016) taxonomies, concatenated with iRT peptide .fasta file (downloaded from the Biognosys webpage) are publicly available (https://www.biognosys.com/media.ashx/irtfusion.fasta).
The mass spectrometry proteomics data (.raw files) and spectral libraries used for data processing (.kit files) have are via the MassIVE with the dataset identifier MSV000084976.

Field-specific reporting

Please select the one below that is the best fit for your research. If you are not sure, read the appropriate sections before making your selection.

☒ Life sciences ☐ Behavioural & social sciences ☐ Ecological, evolutionary & environmental sciences

For a reference copy of the document with all sections, see nature.com/documents/nr-reporting-summary-flat.pdf

Life sciences study design

All studies must disclose on these points even when the disclosure is negative.

Sample size	In this study, 7 samples were analyzed in a multi-center study. QC Sample is HeLa lysate. Sample A and Sample B are mixtures of HeLa, yeast, and E. coli lysates with a known ratio. QC Sample, Sample A and Sample B were analyzed at eleven sites in a 24/7 mode for 7 consecutive days. On Days 1, 3, 5, and 7, all samples were run in three technical replicates, which are a typical number of replicates applied for proteome profiling. In each of the proteome profiling experiments, ~ 8000 proteins from Sample A and B, as well as > 5000 proteins from HELA sample, were identified per single DIA experiment. This provides enough statistical power when comparing protein expresses among different replicates. In total, 12 technical replicates per sample (QC, Sample A, Sample B) per lab (3 technical replicates per day on Day 1, 3, 5, and 7), 120 technical replicates per sample (Sample A and Sample B from 10 labs), and 132 technical replicates of QC sample (from 11 labs) were analyzed in order to assess the reproducibility of the standardized HRMS1-DIA workflow. Additionally, tissue specimens from four patients of two ovarian cancer histotypes (high grade serous and clear cell) were measured at 3 different sites. Each sample had three technical replicates for a total of 36 raw files (9 technical replicates per sample and 4 samples). In order to assess different protein expressions among different samples and reproducibility of the workflow, the high number of technical replicates of all 7 samples provide sufficient statistical power to calculate the standard deviation of quantitation and to perform the differentiation and cluster analysis.
Data exclusions	The baseline for the system suitability test was generated from the analyses of the QC standard performed by four reference laboratories in continuous operation mode over several days with LC-MS platforms operating at different levels of performance. These data enabled the establishment of reference metrics and associated acceptance criteria for platform qualification from three replicate analyses of the QC standard. Reference metrics included median LC elution peak width, number of MS1 and MS2 data points across the LC elution peak, total precursor ions and protein groups identified, and inter-injection median CV on the precursor ion signals (Supplemental Table 1). The median LC peak width indicates the chromatographic separation performance; the number of MS1 and MS2 data points across the LC elution peak allows to check both the method setup and the chromatographic performance; inter-injection median CV on the precursor ion signal evaluates the reproducibilities of the entire LC-MS setup; the total number of precursor ions and protein groups identified are indicators whether the overall performance was acceptable. These metrics enabled real-time monitoring of platform status, covering both chromatographic and mass spectrometric performance characteristics. These QC acceptance criteria were also applied to identify possible issues decrementing analytical performance and credentialing the return to operational status upon completion of interventional maintenance.<br>For laboratory 5 on the day 7, only 4423 proteins were identified, which is > 10% less than the criteria. All chromatography-related criteria were in the acceptance criteria range, which indicated an issue of the mass spectrometry. By performing the mass spectrometry maintenance check, we found that the issue was related to the HCD cell. Therefore, we took the results obtained for day 9 (after maintenance of HCD cell) to substitute those of day 7. Due to laboratory 10 facing major challenges mainly resulting from a poor chromatographic separation, which could not be resolved under the time constraints of the study, laboratory 10 did not participate in controlled sample analyses. The QC data from this laboratory were not excluded from the analysis.
Replication	Reproducibility was one of the central measures of this multi-centric study and is illustrated in figures 4 and 5. The co-quantified proteins across different days and different sites are evaluated to present the overall proteome profiling reproducibilities. The experimentally determined abundance changes of the systematically quantified proteins (every day by every laboratory) between controlled samples A and B were compared to the theoretical ratio of the protein mix to evaluate the quantitation accuracy among all the labs and all the days. The distribution of the coefficients of variation obtained on the determined protein abundance changes for the three organisms across the various evaluation days (CV in %) was plotted for each laboratory in order to assess the quantitation precision at each lab.
Randomization	For the development of the QC-benchmarked HRMS1-DIA workflow, no randomization was applied as we only analyzed two different mixtures of a standard sample. For the analysis of the clinical tumor specimen, we went without randomization to better mimic a typical clinical scenario, in which a sample has to be processed and analyzed upon arrival for patient diagnostics.

Blinding	For data evaluation purposes, no blinding was applied during the development of the QC-benchmarked HRMS1-DIA workflow. The tumor tissue samples were measured blinded without the annotation of histotype or clinical data.

Reporting for specific materials, systems and methods

We require information from authors about some types of materials, experimental systems and methods used in many studies. Here, indicate whether each material, system or method listed is relevant to your study. If you are not sure if a list item applies to your research, read the appropriate section before selecting a response.

Materials & experimental systems

n/a	Involved in the study	
☒	☐	Antibodies
☐	☒	Eukaryotic cell lines
☒	☐	Palaeontology
☒	☐	Animals and other organisms
☐	☒	Human research participants
☒	☐	Clinical data

Methods

n/a	Involved in the study	
☒	☐	ChIP-seq
☒	☐	Flow cytometry
☒	☐	MRI-based neuroimaging

Eukaryotic cell lines

Policy information about cell lines

Cell line source(s)	For spectral library generation, human cell line KG1a (ATCC® CCL-246.1™) was used provided by was provided by Prof. Feng Guan from Northwest University, China.
Authentication	None of the cell lines used in this study were authenticated.
Mycoplasma contamination	Cell lines were not tested for mycoplasm.
Commonly misidentified lines (See ICLAC register)	We did not make use of commonly misidentified cell lines.

Human research participants

Policy information about studies involving human research participants

Population characteristics	Archival formalin-fixed paraffin embedded clear cell ovarian cancer (OCCC) and high grade serous ovarian cancer (HGSOC) tissues were obtained under an IRB-approved protocol from INOVA Fairfax Hospital (Falls Church, VA, USA). The ovarian cancer patients were selected solely based on histology to have two representative cases with pathologic confirmation of diagnosis as being chemo-naive high grade serous ovarian cancer (n=2) and clear cell ovarian cancer (n=2) from primary debulking surgery. These were intended only to fulfill the needs of the small scale pilot analysis, which was solely focused on histotype without consideration of any other clinical co-variates.
Recruitment	Cases were selected based on pathologic review to confirm disease histotype of interest. These were intended only to fulfill the needs of the small scale pilot analysis, which was solely focused on ovarian cancer histotype without consideration of any other clinical co-variates. No self-selection bias outside of histotype should be present to impact the histotype specific proteomic results presented.
Ethics oversight	Western IRB (http://www.wirb.com/Pages/default.aspx)

Note that full information on the approval of the study protocol must also be provided in the manuscript.
