# Supplementary material for: Standardization and harmonization of distributed multi-center proteotype analysis supporting precision medicine studies
Source: Nat Commun. 2020 Oct 16;11:5248. doi: 10.1038/s41467-020-18904-9 (PMC7568553; doi:10.1038/s41467-020-18904-9)
Supplement: Supplementary file 2 — Peer Review File [file 41467_2020_18904_MOESM2_ESM.pdf]

Reviewer #1 (Remarks to the Author):

The manuscript titled "Standardization and Harmonization of Distributed Multi-National Proteotype Analysis supporting Precision Medicine Studies" by Xuan et al. describes an analysis workflow that enabled data generation across 11 labs worldwide, and that evaluated the quantitative data generated across the laboratories of the international Cancer Moonshot consortium. The paper is highly relevant as multi-national proteomic studies are important for future clinical cohort measurements (biomarkers etc) – and certain system suitability measurements for reproducibility have to be in place and recorded in easy to follow protocols, as well as data processing pipelines have to be set into place. There is novelty in this approach – and this work will be of interest to many other labs interested in establishing protocols for measuring clinical samples in the future – or in general for any lab measuring clinical samples improving existing MS protocols. This work is relevant as it also points out the power of having multi-lab studies and multi-national studies.
The study based on data-independent acquisitions (DIA) is performed in a thorough way with central data processing. Initially, labs are acquiring QC samples, and if passing QC metrics labs subsequently acquired what was called samples A and B, mixtures of human, yeast and E. coli lysates. Finally, 3 selected labs in a proof of principle study are acquiring some FFPE ovarian cancer tissue samples.

Comments:
1) What was the distribution of QE HF and Lumos instruments used -- this was a bit unclear, same with capillary vs nano flow.. - what impact did that have on data results.
2) The data accessibility was indicated at MassIVE with ID number MSV000084976, I was not able to look at that as it was password protected. I was not able to look at the data upload. A login should be provided for reviewers. In the manuscript it says: "The mass spectrometry proteomics data (.raw files) and spectral libraries used for the data processing (.kit files) have been deposited to the ProteomeXchange Consortium via the MassIVE partner repository with the dataset identifier MSV000084976". It would be important to review the file uploads for DIA files uploaded and also the DDA files that were used to build libraries. This will be particularly interesting for the FFPE pilot study.
3) It is a bit frustrating that in the methods some parts are described in great depths, such as DDA acquisitions and chromatographic settings, while all that is said for LC-MSMS in DIA mode basically is referring to Supplementary Protocols 2 and 3. The manuscript is about DIA and its use for multi-national proteotype analysis – the methods should at least summarize some details for DIA-MS acquisition in the main text methods of the manuscript, if not having rather comprehensive method describe about DIA in the main text methods.
4) The manuscript should more in depth justify or explain the high resolution MS1 based DIA approach of having 4 MS1 scans interspersed with DIA mass ranges in between. Why using the high resolution MS1 for Quant is better than doing the MS2 quantification information (likely as the scan cycle for MS2 is 5.2 seconds – so one would not get enough points across the eluting peaks). A brief recap of this would be beneficial.
5) The supplementary protocol that describes the data processing using Spectronaut, it does not appear that any specific parameters had to be defined to describe the DIA isolation scheme and the MS1 – MS2 scan setup (or did I miss that). So it might be worth to specifically mention if it is not necessary to define some of those things (if Spectronaut just reads that information in automatically from the raw files) or if it needed any extra information for that.
6) It is a bit unclear which sites and labs used Q Exactive HF vs Lumos instruments … - this may have been said somewhere. Did this influence the results / performance .. Also, some instruments seemed to have nano- or capillary- flow, did that have an influence on the results.
7) Were there challenges for labs to pass the QC (other than what the authors mention), if yes, which of the metrics that the authors mention were necessary for passing the QC were the most challenging ?
8) In figure 1 the authors list the QC criteria: they mention 'MS1 data sampling rate' and 'MS2 data sampling rate' – maybe the authors could reconsider the choice of words. The word 'sampling' suggests typically more an aspect of acquisition. The scans are all there in this DIA workflow – as I understand this is more an extraction during data processing of the comprehensive DIA acquisitions.

Maybe a better description could be chosen.
9) The authors should mention in the methods if a 2 peptide minimum was applied at least for the DDA acquisitions for spectral library building ? Please add those details to the methods section (or if already mentioned more obviously).
10) The authors mention for samples A and B '4565 of these protein groups were not only quantified by all sites but were also quantified on each acquisition day' – others that were more uniquely identified – what was a likely reason for that – was it abundance driven ? Meaning the less abundant proteins were more likely to be seen by all labs ?
11) Figure 4b is not very well displayed and very hard to read – it might be interesting to think about a better visualization of this figure which currently just shows nothing on the x-axis for most of the data points along the x-axis.
12) In figure 5 the authors display an 'error' for comparing sample B to A as they have different compositions of lysates from human, yeast and E coli. Why did the authors choose to show that rather than the ratio distribution. Maybe this was as for human (which are most of the proteins) the ratio A:B is actually 1:1, for yeast the ratio is 1:2 and for E coli the ratio is 4:1. For E. coli, this is particularly difficult to see in figure 5 as the y axis is very high going up to 200 % error. It should be zoomed in more for E. coli.
13) The quantitative assessment of sample A to sample B is really not as fair as the majority of all proteins are not changing at all – Human is 65% of the lysate in both samples in A and B – so for human the error is really a deviation from a ratio of 1 – in a way this is more like another assessment of CV from different samples … This should maybe made more clear in the discussion of figure 5 that the human ratio is 1 – and supposedly the human proteins should not change in abundance between samples A and B --
14) The authors mention on page 14 "The highest deviation from theoretical values was observed in data collected by Lab 1, which may have resulted from some inaccuracy in sample preparation." This remains a bit unclear. Did that lab still have good QC metrics with the interspersed QC ?
15) The DDA acquisitions for library building for the FFPE cancer analysis were those just acquired at one site ? and I assume that then all 3 sites that acquired the data for the cancer Pilot study. Did that make a different in DIA performance comparing the site that did acquire the DDA files for the library vs the other 2 sites where they had to use a library generated by another lab. Maybe/likely the retention time alignments were so good that things were very comparable. This would be very interesting to assess.
16) In general the FFPE part of the manuscript is very interesting. I would like to get more details on the sample preparation – the authors say they used thin 10 um tissue sections, what were length and width of the tissue sections. Also what was the yield in ug protein per FFPE micro-dissection.
17) The authors mention "For tissue-specific spectral library generation, 120 µg of total peptide digests were combined and fractionated by high pH reverse-phase liquid chromatography" was it a total of 120 ug or 4x 120 ug each that was used from each sample.
18) How were the 3 labs selected that acquired the FFPE samples ?
19) While this is said very clearly in the methods section I would also suggest to point out in the main text that the FFPE study really is a Pilot with only 2 samples per each of the ovarian cancer conditions – but if the authors do indeed show this data set (and they do mention it is proof of concept) – what are some of the biologically interesting pathways or proteins that emerged ?
20) Currently, this multi-lab study uses instrument platforms from the same vendor (here Thermo QE HF and Lumos) - - how do the authors envision or do the authors envision that multi-platform consortia may be possible also in the future – what steps may need to be taken for that – it would just be interesting to get some opinions from the authors on this. Or do they think that is not possible ?
21) Please provide a link to the software mentioned in the methods "Differential analysis was performed using the LIMMA package (version 3.8) in R (version 3.5.2)" please elaborate briefly

Minor comments
- Correct in the supplementary protocols E. Coli to E. coli ple

Reviewer #2 (Remarks to the Author):

This manuscript performed a proteomic data quality assessment of a complex peptide mixture using a Orbitrap-based DIA method obtained from multiple centers. The data are good, and the paper is well written. It is encouraging to see that DIA data generated by Orbitraps are robust across labs.

However, what remains unclear to the reviewer is the novelty of this manuscript considering this journal has published a rigorous study of similar objective, experimental design and data (ref27, Collins, et al, 2017). Of course, the obvious difference is that Collins et al used SWATH-MS, a specific DIA implemented in TripleTOF-MS, while this manuscript is based on much more widely adopted MS platform.

The author stated in the end of page 7 that "..... a comprehensive label-free quantitation DIA-MS workflow with a much higher throughput to address the needs of large cohort clinical sample profiling". This is a strong claim which might be the core novelty of this manuscript. Then the question is 1) how much higher is the throughput of this method compared to Collins, et al? 2) what's the unique benefit for large cohort sample profiling?

The last section of the results is to address the clinical benefit. However, The samples analyzed in this study were from 2 HGSOC and 2 OCCC. This small sample size does not support the large cohort sample profiling.

If there are other key novelties the reviewer has missed, probably the authors can clarify those.

In addition to clarification of this key novelty issue, this manuscript might benefit from improving the following technical details:

1) Figure 5, "Analytical precision and accuracy of protein quantification.... ": this data shown in this figure do not actually analyzed both analytical precision and accuracy. If the authors have rigorously analyzed both analytical precision and accuracy, they should show the data.

2) Figure 6: what's the reproducibility of the 5712 proteins quantified across tumor samples and across labs? Fig6C: this figure is not as informative as a rigorous quantitative analysis of the data acquired from three participating labs, because the so-called "18 significant protein alternations and 112 gene signature" were based on comparison of two HGSOC versus two OCCC samples. Fig6D: how about the rest of the proteomes quantified in the three labs in addition to the 18 proteins?

3) legend of Fig6C: a citation is missing.

Point-by-point response - NCOMMS-20-10123

Reviewer #1 (Remarks to the Author):

The manuscript titled “Standardization and Harmonization of Distributed Multi-National Proteotype Analysis supporting Precision Medicine Studies” by Xuan et al. describes an analysis workflow that enabled data generation across 11 labs worldwide, and that evaluated the quantitative data generated across the laboratories of the international Cancer Moonshot consortium. The paper is highly relevant as multi-national proteomic studies are important for future clinical cohort measurements (biomarkers etc) – and certain system suitability measurements for reproducibility have to be in place and recorded in easy to follow protocols, as well as data processing pipelines have to be set into place. There is novelty in this approach – and this work will be of interest to many other labs interested in establishing protocols for measuring clinical samples in the future – or in general for any lab measuring clinical samples improving existing MS protocols. This work is relevant as it also points out the power of having multi-lab studies and multi-national studies.

- We thank reviewer 1 for this encouraging feedback.

The study based on data-independent acquisitions (DIA) is performed in a thorough way with central data processing. Initially, labs are acquiring QC samples, and if passing QC metrics labs subsequently acquired what was called samples A and B, mixtures of human, yeast and E. coli lysates. Finally, 3 selected labs in a proof of principle study are acquiring some FFPE ovarian cancer tissue samples.

Comments:
1) What was the distribution of QE HF and Lumos instruments used -- this was a bit unclear, same with capillary vs nano flow.. - what impact did that have on data results.

- We thank the reviewer for pointing out the ambiguity. At each laboratory, the DIA data sets have been generated by using Q Exactive HF instruments while the DDA analyses used to generate the spectral libraries have been performed on Orbitrap Fusion Lumos or Q Exactive HF-X platforms. The only difference in generating DIA dataset was related to the use of different liquid chromatographic systems (Easy-nLC 1200 or Ultimate 3000 RSLC), both operated in capillary flow mode, which did not translate into obvious different performance levels. The manuscript has been modified to address the ambiguity.
    - Modification page 8: Changes in the manuscript: Page 8 “Primary DIA data acquisition was performed on either the Easy-nLC 1200 or the Ultimate 3000 RSLC nano liquid chromatography system coupled to Q Exactive HF mass spectrometers (Thermo Fisher)”
    - Modification page 9 & 10: “The same capillary LC configuration and mobile phase gradient conditions were utilized for the HRMS1-DIA analysis and the DDA analysis.”
    - In addition, the description of the LC systems used at each laboratory has been added in new “Supplementary Table 6”.

2) The data accessibility was indicated at MassIVE with ID number MSV000084976, I was not able to look at that as it was password protected. I was not able to look at the data upload. A login should be provided for reviewers. In the manuscript it says: “The mass spectrometry proteomics data (.raw files) and spectral libraries used for the data processing (.kit files) have been deposited to the ProteomeXchange Consortium via the MassIVE partner repository with the dataset identifier MSV000084976”. It would be important to review the file uploads for DIA files uploaded and also the DDA files that were used to build libraries. This will be particularly interesting for the FFPE pilot study.

- The reviewer credentials to access the study data have been provided in the reporting summary. The reviewer account password for the MassIVE data set “MSV000084976” is “Moonshot”. Upon acceptance, the data will be publicly released.

3) It is a bit frustrating that in the methods some parts are described in great depths, such as DDA acquisitions and chromatographic settings, while all that is said for LC-MSMS in DIA mode basically is referring to Supplementary Protocols 2 and 3. The manuscript is about DIA and its use for multi-national proteotype analysis – the methods should at least summarize some details for DIA-MS acquisition in the main text methods of the manuscript, if not having rather comprehensive method describe about DIA in the main text methods.

- We thank the reviewer for the suggestion. We have added in the manuscript a description of the DIA method/settings used for the analyses of ovarian cancer samples (page 36) in addition to the description already made for QC and controlled samples analyses (page 33).

4) The manuscript should more in depth justify or explain the high resolution MS1 based DIA approach of having 4 MS1 scans interspersed with DIA mass ranges in between. Why using the high resolution MS1 for Quant is better than doing the MS2 quantification information (likely as the scan cycle for MS2 is 5.2 seconds – so one would not get enough points across the eluting peaks). A brief recap of this would be beneficial.

- We thank the reviewer for the suggestion. The related manuscript section has been substantially re-worked to more in-depth explain the HR-MS1 DIA method used here.
    - Modification page 8/9: "The HRMS1-DIA method used here features an original structure, involving multiple MS1 scans interspersed with 18 DIA MS/MS scans per duty cycle (in total 54 DIA MS/MS scans) (**Figure 1A**). Quantification was based on precursor ion signals measured through high-resolution full MS scans with 120k resolution setting; the MS2 scans with 30k resolution setting was utilized for peptide identification only. High resolution MS1 based peptide quantitation strategies, such as in regular DDA, or pSMART implementation of DIA, have demonstrated excellent quantitative performance[17] and improved quantitation precision and dynamic range. In the HRMS1-DIA method employed, the MS1 scan repetition rate (approximately every 1.7s) was set independently of the MS2 cycle time such that a sufficient number of data points were acquired over peptide chromatographic elution profiles for a proper determination of peptide MS1 peak areas and therefore their precise quantification. By contrast, the parameters of MS2 acquisition were set in a way to maximize peptide detection efficiency, through highly sensitive and selective measurement, rather than the peptide elution profile description by high sampling rate. Therefore, the overall MS2 cycle time was constrained by the need that each parent ion was sampled approximately three times within the duration of a typical chromatographic peak for identification purpose. The associated overall DIA MS2 cycle time of approximately 5.2s resulted from the appropriate settings of the Orbitrap resolution, of the maximum precursor ion injection time, and of the precursor isolation window width. Briefly, the relatively high Orbitrap resolving power of 30k together with the moderate precursor isolation window width of 15 *m/z* units directly enhance measurement selectivity while the maximum ion injection time, synchronized with the Orbitrap transient time of 64ms such as to allow fully parallel ion collection and detection, maintain high measurement sensitivity."

5) The supplementary protocol that describes the data processing using Spectronaut, it does not appear that any specific parameters had to be defined to describe the DIA isolation scheme and the MS1 – MS2 scan setup (or did I miss that). So it might be worth to specifically mention if it is not necessary to define some of those things (if Spectronaut just reads that information in automatically from the raw files) or if it needed any extra information for that.

- The "Methods" section has been modified to include an additional paragraph related to the data processing using Spectronaut to clarify the point.
    - Modification page 36: "The data generated by the acquisition method combining MS1 scans with interspersed DIA MS2 scans are directly processed by Spectronaut without conversion or pre-definition of the actual method structure as input information in the software. Both MS1 and MS2 data are used for peptide identification while the parameters of the quantification process are set to rely on MS1 data."

6) It is a bit unclear which sites and labs used Q Exactive HF vs Lumos instruments … - this may have been said somewhere. Did this influence the results / performance .. Also, some instruments seemed to have nano- or capillary-flow, did that have an influence on the results.

- The response to this point is already covered in response to point #1 above. We include it again below. At each laboratory the DIA data sets have been generated by using Q Exactive HF instruments while the DDA analyses used to generate the spectral libraries have been performed on Orbitrap Fusion Lumos or Q Exactive HF-X platforms. The only difference in generating DIA dataset was related to the use of different liquid chromatographic systems (Easy-nLC 1200 or Ultimate 3000 RSLC), both operated in capillary flow mode, which did not translate into obvious different performance levels. The manuscript has been modified to address the ambiguity.

  - Changes in the manuscript, page 8 “Primary DIA data acquisition was performed on either the Easy-nLC 1200 or the Ultimate 3000 RSLC nano liquid chromatography system coupled to Q Exactive HF mass spectrometers (Thermo Fisher)”
  - Modification page 9&10 “The same capillary LC configuration and mobile phase gradient conditions were utilized for the HRMS1-DIA analysis and the DDA analysis.”

In addition, the description of the LC systems used at each laboratory has been added in new “Supplementary Table 6”.

7) Were there challenges for labs to pass the QC (other than what the authors mention), if yes, which of the metrics that the authors mention were necessary for passing the QC were the most challenging ?

- It is mentioned in the manuscript that the QC was not passed at Lab 5 on day 7, which was due to contamination and could be solved in a fast manner. Some difficulties to pass the QC were also experienced at Lab 10, mainly due to a dysfunction of the LC system, which we were not able to solve at the time right away and therefore the measurements were discontinued for the “Controlled Sample” tests. These cases represented the major challenges at all labs in this study. The related section in the manuscript has been slightly expanded.
  - Modification page 12: “The QC acceptance criteria were systematically satisfied for analyses performed by nine of the eleven laboratories, translating into the identification of 5028 to 5993 protein groups with 1% FDR (**Figure 2**). One laboratory (Lab 10) faced significant analytical challenges, primarily due to poor chromatographic separations. Another participating laboratory (Lab 5) experienced technical issues on Day 7, translating into lower overall performance; specifically, only 4423 protein groups were identified. As the median LC peak elution width, the number of data points across the median LC peak elution at both MS1 and MS2 levels, and the inter-injection median CV on precursor ion signals were within the established criteria, the performance issues were not related to the chromatographic separation. Further investigation revealed the need for maintenance of the higher-energy collision dissociation cell of the mass spectrometer. After necessary maintenance on Day 8, the operation performance was validated on Day 9. These challenges at Lab 5 and Lab 10 represent the only technical challenges encountered in the study.”

8) In figure 1 the authors list the QC criteria: they mention ‘MS1 data sampling rate’ and ‘MS2 data sampling rate’ – maybe the authors could reconsider the choice of words. The word ‘sampling’ suggests typically more an aspect of acquisition. The scans are all there in this DIA workflow – as I understand this is more an extraction during data processing of the comprehensive DIA acquisitions. Maybe a better description could be chosen.

- We thank the reviewer for this suggestion. The inaccuracy in terminology has been addressed by substituting “data sampling rate” with “number of MS1 and MS2 data points across the LC elution peak ” page 10, “number of data points across the median LC peak” on page 12, in Table 1, and in Figure 1b.

9) The authors should mention in the methods if a 2 peptide minimum was applied at least for the DDA acquisitions for spectral library building? Please add those details to the methods section (or if already mentioned more obviously).

- The missing piece of information has been added page 36 “Protein and peptide identifications were filtered at a false discovery rate (FDR) < 1% with no threshold on the minimum number of peptides. “

10) The authors mention for samples A and B ‘4565 of these protein groups were not only quantified by all sites but were also quantified on each acquisition day’ – others that were more uniquely identified – what was a likely reason for that – was it abundance driven ? Meaning the less abundant proteins were more likely to be seen by all labs ?

- Proteins quantified by all sites, but not all days were quantified by two-fold less peptides on average than the 4,565 proteins quantified by all sites and all days. Thus, lower abundant proteins were less likely to be quantified as consistently as more abundant proteins.

11) Figure 4b is not very well displayed and very hard to read – it might be interesting to think about a better visualization of this figure which currently just shows nothing on the x-axis for most of the data points along the x-axis.

- We modified the Fig. 4B by zooming 10-fold to amplify the low intersection bars in the graph.

12) In figure 5 the authors display an ‘error’ for comparing sample B to A as they have different compositions of lysates from human, yeast and E coli. Why did the authors choose to show that rather than the ratio distribution. Maybe this was as for human (which are most of the proteins) the ratio A:B is actually 1:1, for yeast the ratio is 1:2 and for E coli the ratio is 4:1. For E. coli, this is particularly difficult to see in figure 5 as the y axis is very high going up to 200 % error. It should be zoomed in more for E. coli.

- We thank the reviewer for the valuable comment. The choice to report a “% error” in quantification accuracy rather than the experimental ratio was made in order to provide a more uniform piece of information across the different proteomes, which were prepared in different abundance ratios between sample A and B. However, we understand the terminology used here might be misleading, and therefore decided to substitute in Figure 5 - upper panel the term “error” as y-axis title of these plots with “Relative deviation from theoretical ratio A:B” (with A:B being replaced by the value of the actual organism). In addition the displaying of the plot associated with E. coli was adjusted (y-axis zoomed in) to improve the visualization, and therefore the same scaling was expanded to other plots (human and yeast) to maintain the consistency. The legend of the figure has also been adjusted accordingly.

13) The quantitative assessment of sample A to sample B is really not as fair as the majority of all proteins are not changing at all – Human is 65% of the lysate in both samples in A and B – so for human the error is really a deviation from a ratio of 1 – in a way this is more like another assessment of CV from different samples … This should maybe made more clear in the discussion of figure 5 that the human ratio is 1 – and supposedly the human proteins should not change in abundance between samples A and B --

- The point is partially covered above (point #12 reviewer 1). Substituting in Figure 5 - upper panel (and associated legend) the term “Error” with “Relative deviation from theoretical ratio A:B” makes more explicit the performance metrics evaluated here while indicating directly in the plots the theoretical ratio A:B which served as reference value (*e.g.*, 1:1 for human proteins). In addition, the section in the main text discussing the figure 5 has been modified accordingly.
    - Modification page 13/14: “In-depth evaluation of quantitative performance relied on the experimentally determined abundance differences between control samples A and B. The results demonstrated high quantification accuracy compared to theoretical abundance differences as reflected by the low deviation of experimental values from the theoretical values; the median values were generally lower than 10% RSD for human and yeast proteins (for a theoretical 1:1 and 2:1 abundance ratio, respectively) and typically lower than 20% RSD for E. coli proteins (for 4:1 abundance ratio, Figure 5, upper panel).”

14) The authors mention on page 14 “The highest deviation from theoretical values was observed in data collected by Lab 1, which may have resulted from some inaccuracy in sample preparation.” This remains a bit unclear. Did that lab still have good QC metrics with the interspersed QC ?

- This corresponding section has been substantially modified to improve clarity, and demonstrate the high likelihood of a higher deviation in quantification results generated by Lab 1, which we hypothesize resulted from a process error related to sample preparation performed at this Lab.
    - Modification pages 14:”Deviations from theoretical values can arise from multiple factors associated either with variations in sample preparation or LC-MS performance (including LC retention time drift, platform-to-platform variation, or MS ionization efficiency). Due to the low relative stoichiometry of E. coli protein digest (i.e., 5% in sample B) added in the highly complex matrix (human and yeast proteome digests), a higher relative deviation is likely to be associated with the E. coli protein digest quantitative data than with the human or yeast. Indeed, one of the primary objectives of this study was to determine the efficacy of deploying standardized protocols to minimize these deviations among different laboratories. The low standard deviations from the theoretical values in this study demonstrates the high efficacy of the standardization approach. The highest deviation from theoretical values was observed in data collected by Lab 1, where the QC analysis passed the acceptance criteria and showed no evidence of reduced chromatographic or mass spectrometric performance. Therefore, the relatively higher deviation observed in data collected by Lab 1 compared to the other labs may have resulted from the samples themselves, and therefore from process error related to sample preparation at this lab.”

15) The DDA acquisitions for library building for the FFPE cancer analysis were those just acquired at one site ? and I assume that then all 3 sites that acquired the data for the cancer Pilot study. Did that make a different in DIA

performance comparing the site that did acquire the DDA files for the library vs the other 2 sites where they had to use a library generated by another lab. Maybe/likely the retention time alignments were so good that things were very comparable. This would be very interesting to assess.

- Response: In response to Reviewer #1, Comment# 15, all "DDA acquisitions for library building for the FFPE cancer analysis" were acquired at one analytical site and used to support central data analysis from all three sites. In regard to impact on DIA performance across analytical sites, we assessed the performance of proteins co-quantified in patient tissue samples across analytical sites and found that reproducibility of proteins exhibited a RSD = 9.0%.

16) In general the FFPE part of the manuscript is very interesting. I would like to get more details on the sample preparation – the authors say they used thin 10 um tissue sections, what were length and width of the tissue sections. Also what was the yield in ug protein per FFPE micro-dissection.

- Response: In response to Reviewer #1, Comment# 16, as tissue section length and width can vary from tumor block to tumor block, we commonly report total tissue area collected by laser microdissection analyses, please refer to this precedent from our team for more details: https://www.ncbi.nlm.nih.gov/pubmed/21932769. For the FFPE study reported herein, we collected 87.03 ± 1.93 mm^2 of tissue per patient tumor sample and total peptide yield was 0.685 ± 0.08 µg/ mm^2 tissue collected. We have also included these details in the methods section of the manuscript.

17) The authors mention "For tissue-specific spectral library generation, 120 µg of total peptide digests were combined and fractionated by high pH reverse-phase liquid chromatography" was it a total of 120 ug or 4x 120 ug each that was used from each sample.

- Response: In response to Reviewer #1, Comment # 17, we have clarified this statement in the manuscript: "To generate the cancer tissue-specific spectral library, 30 µg from each patient sample digest were combined (for a total of 120 µg) and fractionated by high pH reversed-phase liquid chromatography into 96 fractions using a linear gradient of ACN (0.69% per minute) as described above."

18) How were the 3 labs selected that acquired the FFPE samples ?

- Response: In response to Reviewer #1, Comment # 18, the three analytical sites were selected for the FFPE tissue study as they were available to perform these additional pilot analyses.

19) While this is said very clearly in the methods section I would also suggest to point out in the main text that the FFPE study really is a Pilot with only 2 samples per each of the ovarian cancer conditions – but if the authors do indeed show this data set (and they do mention it is proof of concept) – what are some of the biologically interesting pathways or proteins that emerged ?

- Response: In response to Reviewer #1, Comment # 19, we have pointed out our FFPE study is "a pilot" and further included the following revised manuscript text to discuss "biologically interesting pathways", i.e. "Pathway analyses of these alterations revealed activation of peroxisome proliferator-activated receptor alpha (PPAR-alpha)/ Retinoid X receptor alpha (RXR-alpha) as well as cyclin-dependent kinase 5 (CDK5) signaling, but inhibition of metabolic pathways, namely Oxidative Phosphorylation, in OCCC versus HGSOC patient tumor tissues (Supplemental Table 5). Notably, (PPAR-alpha) has been identified as a possible therapeutic target for the treatment of clear cell renal cell carcinoma (ccRCC), a parallel disease pathology to OCCC and one in which CDK5 signaling has also been identified as an important regulatory pathway."

20) Currently, this multi-lab study uses instrument platforms from the same vendor (here Thermo QE HF and Lumos) -- how do the authors envision or do the authors envision that multi-platform consortia may be possible also in the future – what steps may need to be taken for that – it would just be interesting to get some opinions from the authors on this. Or do they think that is not possible ?

- Well, good question & not easy to answer. At the ICPC meeting at HUPO in Adelaide last year, Mike MacCoss initiated a discussion along these lines. Basically, we discussed the possibility of having a common set of samples that we could put through our respective protocols to demonstrate that labs across the world could get the same quantitative values regardless of the method they used -- pending the results were reported

relative to a common reference/constant. We/the community would need to agree on such a reference/constant, prepare reference material, share and adopt the constant internationally.
- It's interesting in this respect to think about the kilogram and other "constants". As of May 20th, 2019, World Metrology Day, the new definition of the kilogram based on Planck's constant has officially been adopted.
  - Here's an article from "The Verge" which I (B.W.) enjoyed reading related to "constants".
    - https://www.theverge.com/2018/11/13/18087002/kilogram-new-definition-kg-metric-unit-ipk-measurement

21) Please provide a link to the software mentioned in the methods "Differential analysis was performed using the LIMMA package (version 3.8) in R (version 3.5.2)" please elaborate briefly

- Response: In response to Reviewer #1, Comment # 21, we have revised our Materials and Methods section to include the following details about LIMMA package (version 3.8), i.e. "Differential analysis was performed using the LIMMA package (version 3.8, https://bioconductor.org/packages/release/bioc/html/limma.html) in R (version 3.5.2) with the expectation that proteins significantly altered between HGOSC and OCCC patient tissues exhibited an adjusted p-value < 0.01, and cluster analyses was performed using ClustVis (https://biit.cs.ut.ee/clustvis/)."

Minor comments
- Correct in the supplementary protocols E. Coli to E. coli ple

- In the supplementary protocols, "E. Coli" has been modified into "E. coli".

Reviewer #2 (Remarks to the Author):

This manuscript performed a proteomic data quality assessment of a complex peptide mixture using a Orbitrap-based DIA method obtained from multiple centers. The data are good, and the paper is well written. It is encouraging to see that DIA data generated by Orbitraps are robust across labs.

- We thank Reviewer #2 for this positive feedback.

However, what remains unclear to the reviewer is the novelty of this manuscript considering this journal has published a rigorous study of similar objective, experimental design and data (ref27, Collins, et al, 2017). Of course, the obvious difference is that Collins et al used SWATH-MS, a specific DIA implemented in TripleTOF-MS, while this manuscript is based on much more widely adopted MS platform.

- Reviewer 2 is right with his statement that we performed a study of similar objective as Collins et al. in 2017. However, we disagree on his/her conclusion that the HRMS1-DIA pipeline we present here lacks novelty. In contrast to the study by Collins and colleagues we not only applied a DIA method benchmarked at different sites but rather provide an entire streamlined and easy to follow HRMS1-DIA protocol ranging from LC setup to data evaluation. Our workflow contains quality control measures at every level, which are easy to implement and enable any lab measuring clinical samples improving existing MS protocols. Also, the DIA method used in our study features an original structure, involving multiple MS1 scans interspersed with 18 DIA MS/MS scans per scan cycle (in total 54 DIA MS/MS scans) (page 8 /9). High-resolution MS1-based peptide quantitation strategies -as used in conventional DDA or pSMART implementation of DIA- have demonstrated excellent quantitative performance, which we could also show here in our multi-centric study. In addition, a capillary LC setup with a 150um ID analytical LC column was applied for all LC-MS analysis, which can use high flow rate for sample loading and column equilibration (supplementary protocol 2 and 3), enhancing the overall throughput.

The author stated in the end of page 7 that "..... a comprehensive label-free quantitation DIA-MS workflow with a much higher throughput to address the needs of large cohort clinical sample profiling". This is a strong claim which might be the core novelty of this manuscript. Then the question is 1) how much higher is the throughput of this method compared to Collins, et al? 2) what's the unique benefit for large cohort sample profiling?

- According to the reviewer's criticism, we adjusted our statements on page 7. The sentence now reads "a high-throughput (e.g. 100 proteins quantified/min of analysis time), comprehensive, as well as quality-controlled label-free quantitation DIA-MS workflow to address the needs of conducting large clinical cohort studies distributed across multiple laboratories"

The last section of the results is to address the clinical benefit. However, The samples analyzed in this study were from 2 HGSOC and 2 OCCC. This small sample size does not support the large cohort sample profiling.

- As indicated in the text we have pointed out our FFPE study is/was designed as "a pilot" and that the profiling of a large sample cohort is outside of the scope of the current manuscript, however would now be in principle feasible in the context of distributed data generation strategies.

If there are other key novelties the reviewer has missed, probably the authors can clarify those.

- We thank the reviewer for the suggestion. The related manuscript section has been substantially re-worked to more in-depth explain the HR-MS1 DIA method, which is certainly a key novelty introduced here.
    - Modification page 8/9: "The HRMS1-DIA method used here features an original structure, involving multiple MS1 scans interspersed with 18 DIA MS/MS scans per duty cycle (in total 54 DIA MS/MS scans) (**Figure 1A**). Quantification was based on precursor ion signals measured through high-resolution full MS scans with 120k resolution setting; the MS2 scans with 30k resolution setting was utilized for peptide identification only. High resolution MS1 based peptide quantitation strategies, such as in regular DDA, or pSMART implementation of DIA, have demonstrated excellent quantitative performance[17] and improved quantitation precision and dynamic range. In the HRMS1-DIA method employed, the MS1 scan repetition rate (approximately every 1.7s) was set independently of the MS2 cycle time such that a sufficient number of data points were acquired over peptide chromatographic elution profiles for a proper determination of peptide MS1 peak areas and therefore their precise quantification. By contrast, the parameters of MS2 acquisition were set in a way to maximize peptide detection efficiency, through highly sensitive and selective measurement, rather than the peptide elution profile description by high sampling rate. Therefore, the overall MS2 cycle time was constrained by the need that each parent ion was sampled approximately three times within the duration of a typical chromatographic peak for identification purpose. The associated overall DIA MS2 cycle time of approximately 5.2s resulted from the appropriate settings of the Orbitrap resolution, of the maximum precursor ion injection time, and of the precursor isolation window width. Briefly, the relatively high Orbitrap resolving power of 30k together with the moderate precursor isolation window width of 15 *m/z* units directly enhance measurement selectivity while the maximum ion injection time, synchronized with the Orbitrap transient time of 64ms such as to allow fully parallel ion collection and detection, maintain high measurement sensitivity."

In addition to clarification of this key novelty issue, this manuscript might benefit from improving the following technical details:

1) Figure 5, "Analytical precision and accuracy of protein quantification.... ": this data shown in this figure do not actually analyzed both analytical precision and accuracy. If the authors have rigorously analyzed both analytical precision and accuracy, they should show the data.

- We thank the reviewer for the valuable comment, which partially overlaps with comments #12 and 13 of reviewer #1. We agree the terminology used here is not fully accurate. Therefore, we have modified the title of Figure 5 by substituting "Analytical precision and accuracy of protein quantification from the analyses of controlled samples" with "Performance metrics for the quantification of proteins from the analyses of controlled samples". In addition, we substituted in Figure 5 - upper panel the term "error" as y-axis title of these plots with "Relative deviation from theoretical ratio A:B" (with A:B being replaced by the value of the actual organism) in order to provide more self-explanatory description of the metrics actually evaluated.

2) Figure 6: what's the reproducibility of the 5712 proteins quantified across tumor samples and across labs? Fig6C: this figure is not as informative as a rigorous quantitative analysis of the data acquired from three participating labs, because the so-called "18 significant protein alternations and 112 gene signature" were based on comparison of two HGSOC versus two OCCC samples. Fig6D: how about the rest of the proteomes quantified in the three labs in addition to the 18 proteins?

- Response: In response to Reviewer #2, Comment # 2:
  - Part A, we have included the following revised manuscript text to address comments regarding the reproducibility of “proteins quantified across tumor samples and across labs”, i.e. “…3,808 ± 343 (9.0% RSD) were co-quantified in individual patient tumor samples that further exhibited high quantitative correlation across analytical sites (Spearman Rho=0.62 ± 0.08, p<0.0001).” (page 15).

  - Part B, in response that Figure 6C “is not as informative as a rigorous quantitative analysis of the data acquired from three participating labs”, we agree this does not provide sufficient detail to assess quantitative performance and we address this as well as the additional comment “how about the rest of the proteomes quantified in the three labs in addition to the 18 proteins?”, in the following revised manuscript text (page 15/16), “…394 significantly altered proteins (LIMMA adjusted P-value <0.01) between HGSOC and OCCC patient tissues (Supplementary Table 4; Figure 6B) and these features exhibited high quantitative correlation in individual patient samples across analytical sites (Spearman Rho = 0.92 ± 0.02) and disease histotypes (Spearman Rho = 0.63 ± 0.04) (Figure 6C).” This latter data also refers to the addition of a correlation plot (revised Figure 6C) to Figure 6 detailing the quantitative performance of protein alterations between HGSOC and OCCC tissues across patient samples and analytical sites.

3) legend of Fig6C: a citation is missing.

- In response to Reviewer #2, Comment #23, we have added the citation for Hughes et al, i.e. https://www.ncbi.nlm.nih.gov/pubmed/27713570 to the Figure legend of Figure 6C.

REVIEWERS' COMMENTS:

Reviewer #1 (Remarks to the Author):

The authors addressed all requested revisions. I recommend to accept the manuscript for publication.
